# Synthesis, Modification, and Biological Evaluation of a Library of Novel Water‐Soluble Thiopyridone‐Based Organometallic Complexes and Their Unexpected (Biological) Behavior

**DOI:** 10.1002/chem.201905546

**Published:** 2020-04-06

**Authors:** Sophia Harringer, Barbara Happl, Marius Ozenil, Caroline Kast, Michaela Hejl, Debora Wernitznig, Anton A. Legin, Andreas Schweikert, Natalie Gajic, Alexander Roller, Gunda Koellensperger, Michael A. Jakupec, Wolfgang Kandioller, Bernhard K. Keppler

**Affiliations:** ^1^ Institute of Inorganic Chemistry, Faculty of Chemistry University of Vienna Waehringer Strasse 42 1090 Vienna Austria; ^2^ Ludwig Boltzmann Institute Applied Diagnostics General Hospital of Vienna Waehringer Guertel 18–20 1090 Vienna Austria; ^3^ Department of Biomedical Imaging and Image-guided Therapy Division of Nuclear Medicine Medical University of Vienna Spitalgasse 23 1090 Vienna Austria; ^4^ Department of Analytical Chemistry, Faculty of Chemistry University of Vienna Waehringer Strasse 38 1090 Vienna Austria; ^5^ Research Cluster “Translational Cancer Therapy Research” Waehringer Strasse 42 1090 Vienna Austria

**Keywords:** cancer, half-sandwich complexes, metallodrugs, organometallic, thiopyridones

## Abstract

A series of 16 dinuclear thiopyridone‐based organometallics with excellent water solubility, increased stability and remarkable cytotoxicity were synthesized and characterized. The complexes of this work formed dimeric species featuring a double positive charge in polar protic solvents, accounting for their outstanding solubility in aqueous solution. Most of them displayed higher antiproliferative activity than their parental thiomaltol complex, with unexpected cytotoxicity trends depending on the employed metal center, ligand modification, and cell line. Insights into their behavior in biological systems were gathered by means of amino‐acid interaction studies, cytotoxicity tests in 3D spheroid models, laser ablation, cellular accumulation measurements, as well as cell cycle experiments.

## Graphical abstract

**A chemical concerto**: Thiopyridone‐based piano‐stool complexes undergo dimerization in polar protic solvents. The resulting dimers (featuring a double positive charge) were studied for their effects in cancer cells.

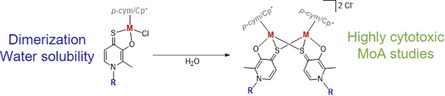

## Introduction

**Figure 1:**
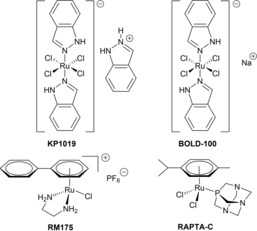
Representatives of clinically studied ruthenium(III)‐based compounds, and most extensively investigated ruthenium(II) piano‐stool complexes.

Much effort has been expended to develop alternatives to classic platinum‐based single agent or combination therapy due to reoccurring clinical limitations, such as severe side effects or ineffectiveness caused by drug resistance.^1^, ^2^, ^3^ Ruthenium‐containing compounds are amongst the most promising candidates, as several ruthenium complexes show redox behavior under physiological conditions, accumulate preferably in tumor tissue and feature more coordination sites allowing additional modifications compared to platinum(II) coordination compounds.^4^ This field of research was tremendously stimulated when the exceptional anticancer properties of (indazolium *trans*‐[tetrachlorido‐bis(1*H*‐indazole)ruthenate(III)]) (KP1019; Figure 1) were discovered.^3^, ^5^, ^6^ This compound showed preclinical activity against autochthonous colon cancer in a rat model with an efficacy of up to 95 % reduction of tumor volume at a well tolerable dose causing no more than 6 % weight loss.^3^ In later studies this lead compound was replaced by its sodium analogue BOLD‐100 (formerly IT‐139 and NKP‐1339; Figure 1) due to superior solubility of the latter, which then showed activity in patients with advanced solid tumors, such as non‐small‐cell lung cancer, colorectal carcinoma and gastrointestinal neuroendocrine tumors, in a clinical phase I/II study.^7^ Reversible adduct formation with blood proteins is thought to foster accumulation in tumor tissue. Therefore, the proposed mode of action is based on binding to non‐classic targets and the exploitation of the enhanced permeability and retention (EPR) effect.^4^, ^8^ Due to its fast growth rate, tumor tissue lacks proper vascular architecture, with enhanced permeability allowing for sufficient nutrient supply, whereas a lack of lymphatic drainage leads to enhanced retention of macromolecules inside the tumor.^9^ Furthermore, BOLD‐100 was suggested to interact with the cell's protein machinery, and the endoplasmic reticulum's (ER) chaperone GRP78, a glucose‐regulated protein.^3^ This protein is highly expressed in cancer cells to regulate responses to ER stress, which is constitutively elevated in cancer cells.^10^

The development of organometallic complexes brought to light another class of ruthenium compounds, which contain the metal in its +II oxidation state, stabilized via the introduction of a facially coordinated arene ligand.^7^, ^11^ These so‐called “piano‐stool” complexes feature an arene ligand, resembling the stool's seat, and three coordination sites available for mono‐, bi‐ or tridentate ligands, mimicking the legs.^12^ Two representatives of this class showed promising results in preclinical investigations, namely RAPTA‐C and RM175 (Figure 1).^13^ While RAPTA‐C′s antiangiogenic and antimetastatic activity is supposed to be pH‐dependent, RM175 reduces tumor growth in vivo based on DNA interactions.^14^, ^15^ These findings specifically highlight the importance of the coordination sphere for pharmacokinetic and pharmacodynamic properties, which can be fine‐tuned via proper ligand modification.^16^ As complex stability is another fundamental aspect for sufficient anticancer activity or lack thereof, a stable coordination site might prevent premature reaction with biomolecules and subsequent inactivation. This has been demonstrated by the replacement of *O,O*‐chelating pyrone complexes by their *S,O*‐derivatives yielding IC_50_ values in the low to sub‐micromolar range.^17^, ^18^ On the other hand, ruthenium(II) pyridone complexes showed increased stability in aqueous solution, which can be explained by their stronger pseudo aromatic character enhancing complex stability by at least 1.5 log units.^19^ In this work, we prove that the combination of thiomaltol and pyridone motifs in a new class of organometallic thiopyridone complexes leads to increased bioactivity as well as enhanced stability in a biologically relevant pH range of 5.8–7.9. Furthermore, the impact of metal center and ligand variation on the physicochemical and biological properties was investigated. Apart from MTT assays in monolayer cultures of human cancer cells, additional studies have been carried out in order to gather insights into the behavior of these compounds in biological systems (e.g., incubation with possible target molecules, cellular accumulation, cell cycle analysis, and cytotoxicity in multicellular tumor spheroid models).

## Results and Discussion

**Scheme 1:**
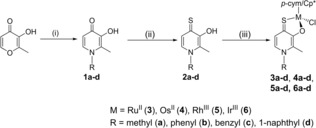
Overview of synthesized thiopyridone‐based piano‐stool complexes. (i) methylamine, water and reflux overnight (**1 a**), or aniline, benzylamine, or 1‐naphthylamine, 0.38 m HCl and microwave irradiation (*t*=30 min, *T*=165 °C) (**1 b**–**d**); (ii) Lawesson's reagent, abs. toluene, Schlenk technique, reflux 4–16 h; (iii) [(*p*‐cym)/(Cp*)MCl_2_]_2_, NaOMe, MeOH abs., 40 °C, 1–3.5 h.

The compound library was designed in order to optimize pharmacokinetic and pharmacodynamic properties via variation of the metal center, and substitution of the thiopyridone scaffold (Scheme 1). Generally, the ligands were chosen to cover a broad range of characteristics, such as lipophilicity and steric volume. In this synthetic route, commercially available maltol was converted to four different pyridones by treatment with the respective primary amines. Synthesis was performed according to literature for 3‐hydroxy‐1,2‐dimethyl‐pyridin‐4(1*H*)‐one, 3‐hydroxy‐2‐methyl‐1‐phenylpyridin‐4(1*H*)‐one, and 1‐benzyl‐3‐hydroxy‐2‐methylpyridin‐4(1*H*)‐one.^20^ However, a twentyfold reaction time was applied in order to improve yields. These optimized reaction conditions also enabled a one‐step synthesis of 3‐hydroxy‐2‐methyl‐1‐(naphthalene‐1‐yl)pyridine‐4(1*H*)‐one with yields of up to 74 %. These results are superior to previously published literature, where a three‐step reaction protocol with an overall yield of 43 % has been described.^21^ Subsequently the synthesized pyridones were thionated by use of Lawesson's reagent under inert conditions yielding the desired *S,O*‐ligands in moderate to good yields (33–83 %). Complexation was carried out according to established literature procedures under inert conditions by means of Schlenk technique to avoid undesired side reactions.^22^, ^23^ The respective thiopyridone was deprotonated with sodium methoxide in dry methanol and the dimeric metal precursor of choice was added to the solution. The applied reaction time is strongly dependent on the coordination motif. While standard procedures for pyrone complexes with an *O,O*‐coordination motif require at least several hours or even overnight syntheses,^24^ thiopyrone complexes featuring an *S,O*‐coordination motif react much faster with reported reaction times of 3.5 h at most. Thus, the mixtures were stirred at room temperature for 1–3.5 h, depending on the utilized ligand. Pure complexes were obtained after work up and purification by crystallization/precipitation from dichloromethane/diethyl ether, or dichloromethane/*n*‐hexane in moderate to excellent yields (33–83 %). In total a library consisting of 16 new thiopyridone‐based organometallics has been synthesized bearing 3‐hydroxy‐1,2‐dimethylpyridine‐4(1*H*)‐thione (**a**), 3‐hydroxy‐2‐methyl‐1‐phenylpyridine‐4(1*H*)‐thione (**b**), 3‐hydroxy‐2‐methyl‐1‐benzylpyridine‐4(1*H*)‐thione (**c**), or 3‐hydroxy‐2‐methyl‐1‐(naphthalene‐1‐yl)pyridine‐4(1*H*)‐thione (**d**) as chelating ligands.

**Figure 2:**
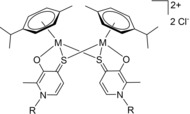
General structure of the proposed dimers featuring a double positive charge under polar protic conditions.

The obtained complexes were characterized by ^1^H, ^13^C and 2D NMR, ESI‐MS measurements, elemental analysis, and single crystal X‐ray crystallography, when possible. ^1^H, ^13^C and 2D NMR of all complexes recorded in deuterated dimethyl sulfoxide [D_6_]DMSO showed peaks according to the expected monomeric species (Figures S7–S38). Successful complexation could be confirmed by downfield shifts of the ligand's proton signals (−0.03 ppm) and vanishing of the signal for the hydroxy group. Four signals could be expected for the aromatic protons of the *p*‐cymene arene due to the presence of a chiral metal center. However, in accordance with literature, only two doublets could be observed for the aromatic η^6^‐*p*‐cymene protons.^25^, ^26^, ^27^ None of the phenylic protons of the 3‐hydroxy‐2‐methyl‐1‐phenylpyridine‐4(1*H*)‐thione (**1 b**) complexes become chemically equivalent due to the proximity and resulting steric hindrance caused by the neighboring methyl group. The spectrum changes drastically when using a 3‐hydroxy‐2‐methyl‐1‐benzylpyridine‐4(1*H*)‐thione (**1 c**) ligand instead, where benzylic protons become chemically equivalent and form a doublet and a triplet signal. This can be explained by the bridging CH_2_ group, which enables more degrees of freedom for this ligand system compared to its aniline congener. The complexes prepared in this work showed exceptionally good aqueous solubility (up to 10 mg mL^−1^), thus ^1^H‐, ^13^C‐, and 2D‐NMR sets of ruthenium **3 a**–**c** and osmium compounds **4 a**–**c** were recorded in deuterium oxide (Figures S39–S50). However, Rh and Ir complexes exhibited limited water solubility. Therefore, only the respective NMRs of Rh methyl and benzyl complexes (**5 a**,**c**) and Ir methyl compound **6 a** were recorded in D_2_O (Figures S51–S56). ^1^H NMR spectra of organoruthenium(II) and ‐osmium(II) complexes show two η^6^‐*p*‐cymene methyl signals and four aromatic signals compared to one methyl signal and two aromatic signals in [D_6_]DMSO. Herein, we propose the formation of a dimeric complex under polar protic conditions (denoted with * at the respective compound abbreviation henceforth). In ^1^H NMR spectra the protons of the methyl groups Hg form two doublets with an integral of 6H. Additionally, a DACH effect can be observed for the methyl signals as well as the aromatic signals, accounting for the deviation from the expected 1:1 signal intensity. Furthermore, the four sharply resolved aromatic signals in polar protic solvents compared to two doublets in polar aprotic solvents can be explained by the presence of a dimeric species. As the dimeric, thiolato‐bridged complex is expected to be more rigid it can prevent signal broadening, which is usually caused by dynamic effects according to literature.^26^ In this case, the proximity of the two *p*‐cymene arenes hinders their rotation, reducing symmetry and leading to signal separation. Theoretically, 2^2^=4 stereoisomers should be observed for these dimers, as a result of the two stereogenic metal centers. However, the total number is reduced to three, because the (*R*,*S*) and (*S*,*R*) configurations are identical due to the molecular symmetry. The thiopyridone ligands face in the same direction in the (*R*,*R*) and (*S*,*S*) conformation, enabling π‐stacking. Consequently, these diastereomers should be thermodynamically more stable compared to the (*R*,*S*)≡(*S*,*R*) form, which was also supported by means of crystal structure analysis (see X‐ray discussion). NMR spectra show only one set of signals for complexes **3 a–d** and **4 a–d** in [D_6_]DMSO, but at least two different configurations for 3 d* and 4 d* can be observed for spectra measured in D_2_O (Figure 2). There are several possible explanations for this phenomenon. First of all, it is possible that the formation of the dimeric complex is not complete and some complex is still present in its monomeric form. Secondly, it is possible that the bulky naphthyl ligand favors the (*R*,*S*)≡(*S*,*R*) configuration. Finally, the proximity of the sterically demanding ligands could lead to the formation of rotamers in the (*R*,*R*) and (*S*,*S*) conformation, due to limited rotation of the naphthyl moieties.

**Figure 3:**
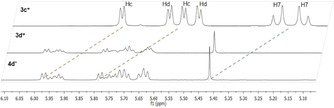
^1^H NMR aromatic *p*‐cymene signal comparison of Ru^II^ complexes **3** c*, 3 d* and Os^II^ derivative 4 d* in D_2_O.

Generally, thiolato‐bridged dimerization has already been reported for organoruthenium complexes.^28^, ^29^, ^30^, ^31^ However, despite numerous publications based on pyrone‐, pyridone‐, and thiopyridone ruthenium and osmium piano‐stool complexes dimerization as observed in this work has not been described yet.^17^, ^18^, ^19^, ^24^, ^26^, ^32^ Therefore, the bridging effect may be attributed to the *N*‐substituted backbone of the new thiopyridone ligands. Compared to maltol and thiomaltol analogues the electron density in the ligand system is enhanced, due to the stronger +M effect of the introduced amine functionality. Additionally, in accordance with the HSAB principle the affinity of the transition metals is expected to be higher towards sulfur than oxygen, because of its softer character. The combination of these two effects may be a crucial factor for the formation of the discovered thiolato bridges. The dimeric species is connected via two thiolato bridges, resulting in a double positive charge, which would furthermore account for the exceptional solubility in water (Figure 3).

ESI‐MS investigations showed peaks according to [*M*−Cl]^+^ fragments for all synthesized complexes. As mass spectra show *m*/*z* peaks, it is impossible to distinguish the monomeric and dimeric species by their mass peaks only. Nevertheless, clear differences should be visible in their metal patterns, which clearly show a monomeric species. These findings are counterintuitive, as samples are prepared in aqueous solution (ACN/MeOH+1 % H_2_O), which should lead to formation of the dimeric complex. However, it is likely that the dimer is cleaved upon electrospray ionization and disintegrates into two monomeric fragments.

### X‐ray diffraction analysis

**Figure 4:**
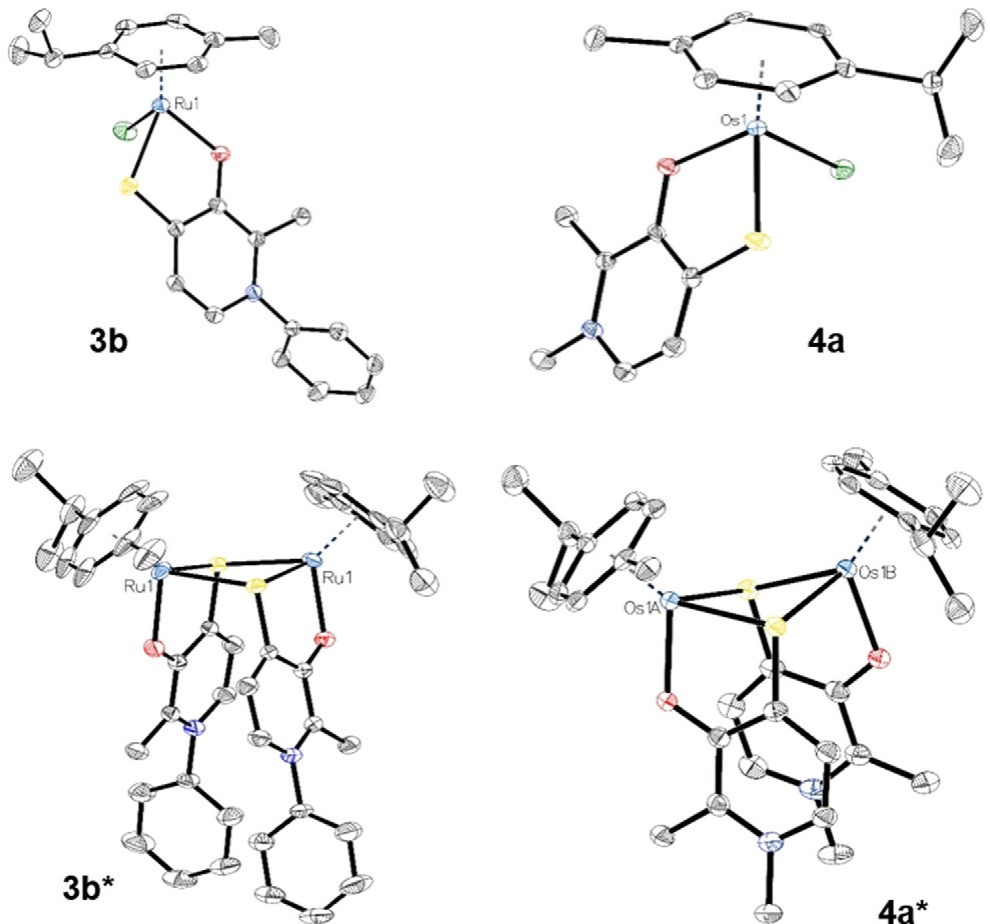
ORTEP views of monomeric Ru complex **3 b** (top left), and Os complex **4 a** (top right) as well as their dimeric congeners 3 b* (bottom left) and 4 a* (bottom right).

Single crystals of **3 a**–**d**, **4 a**–**d**, **5 b**, **6 b**, and **6 d** suitable for X‐ray diffraction analysis were obtained by slow diffusion method from dichloromethane/diethyl ether (**3 b**,**c**, **4 a**,**b**), dichloromethane/*n*‐hexane (**3 d**, **4 c**,**d**, 5 b*, 6 b*, **6 d**), methanol/diethyl ether (3 b*, 3 c*, 4 a*) or dichloromethane/ethyl acetate (3 a*). Five monomeric (**3 d**, **4 b**–**d**, **6 d**), three dimeric (3 a*, 5 b*, 6 b*) and three structures for both configurations (**3 b–c**, 3 b*,c*, 4 a, 4 a*) could be collected. Monomeric crystal structures obtained from polar aprotic solvents confirm the adaption of a “piano‐stool” configuration, with the arene (*p*‐cymene or Cp*) constituting the stool's seat, and the bidentate chelate (thiopyridone) and the chloride‐leaving group resembling its legs. Crystals grown in polar protic solvents confirm the findings of our NMR measurements that thiopyridone‐based organometallic complexes form dimers under these conditions. The most important parameters for complexes **3 b** (ruthenium featuring a thioaniline ligand), and **4 a** (osmium coupled to thiodeferiprone) as well as the respective dimers 3 b* and 4 b* are summarized in Table 1, and their structures are shown in Figure 4. While Ru complex **3 b** crystallizes in monoclinic space groups in the monomeric (*P*2_1_/*n*) and 3 b* (*C*2/*c*) form, monomeric Os compound **4 a** crystallizes in the monoclinic space group *C*2/*c* and the respective dimer 4 a* in the triclinic space group *P1*. According to expectations, C=S bond lengths are increased upon dimerization (3 b/3 b*: 1.719/1.755 Å, and 4 a/4 a*: 1.736/1.773 Å). This indicates a higher stability of the four‐membered dimeric scaffold, featuring two sulfur atoms and two metal centers. On the other hand, M−O and C−O distances showed only a slight increase of around 0.011 Å in 3 b* and 0.017 Å in 4 a*. Additionally, the arene π‐plane distance increases approximately the same, with 0.024 Å (3 b*) and 0.026 Å (4 a*), respectively. Compared to reported values for the respective Os‐thiomaltol parent compound, the M−S bond (**4 a**: 2.366, **Os‐TM**: 2.378) and π‐plane centroid distance (**4 a**: 1.649, **Os‐TM**: 1.662) are slightly shorter, while the M−Cl bond is slightly longer in compound **4 a**, indicating higher stability of the thiopyridone counterparts (**4 a**: 2.488 Å, **Os‐TM**: 2.436 Å).^18^ On the other hand, the distances between M−O, and C−O are approximately the same.

**Table 1:** Selected bond lengths of Ru^II^ and Os^II^ compounds **3 b**, and **4 a** as well as their dimeric counterparts 3 b* and 4 a*.

	**3 b**	3 b*	**4 a**	4 a*
M−S [Å]	2.366	2.372	2.366	2.398
	–	2.414	–	2.437
M−O [Å]	2.074	2.091	2.082	2.085
M−Cl [Å]	2.439	‐	2.448	–
C=S [Å]	1.719	1.755	1.736	1.773
C−O [Å]	1.309	1.313	1.320	1.321
π‐plane centroid [Å]	1.654	1.678	1.649	1.680
M−M	–	3.566	–	3.550
Torsion M‐S‐M [°]	–	12.5	–	23.5

### Stability in aqueous solution

**Figure 5:**
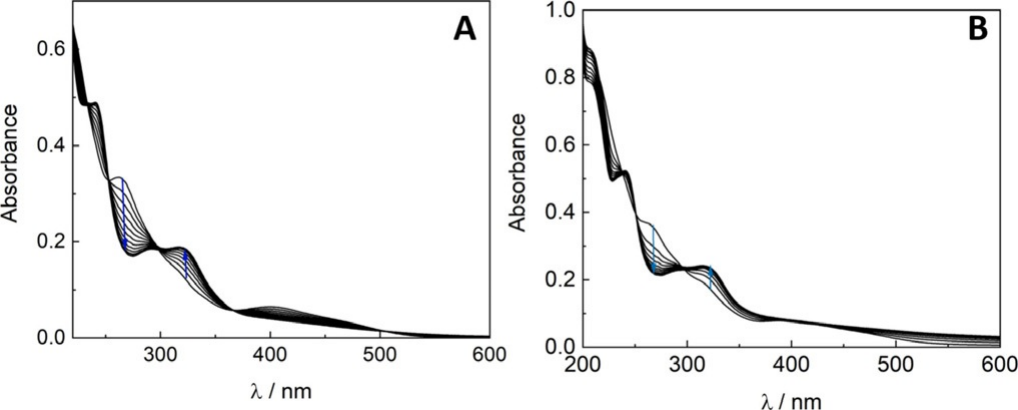
UV/Vis spectra of complex 4 b* in PBS (**A**) and H_2_O (**B**) measured over 24 h.

In order to investigate complex stabilities, UV/Vis spectrophotometry was conducted for all complexes. All ruthenium (3 a*–d*), rhodium (5 a*–d*), and iridium (6 a*–d*) compounds were stable over 24 h in water at 25 °C. UV/Vis spectra showed a slight decrease in complex concentration, which can be attributed to slow precipitation of the product from the UV/Vis cuvette over time (Figures S57–S72). However, osmium complexes 4 a*–d* showed decomposition over time, with UV/Vis spectra showing isosbestic points at 220, 270, 300, and 370 nm, respectively (Figures S61–S64). This process is fastest for complex 4 b* followed by complexes 4 a* and finally 4 c* in PBS. Decomposition of the osmium compounds was slightly slower in pure water (Figure 5).

**Figure 6:**
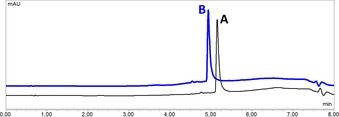
UHPLC stability investigation of complex 3 c* after 0 h (**A**; 5.15 min) and 48 h (**B**; 4.93 min) at pH 7.2 in phosphate buffer, proving stability of the complex under these conditions. Minor shifts in retention time are due to fluctuating pump pressure.

In order to investigate a possible pH influence on complex stability and to gather further insights into the instability of the osmium complexes, UHPLC kinetic measurements were carried out at pH values of 5.8, 6.2, 6.7, 7.2 and 7.9 in phosphate buffered solution via UHPLC. Due to its biological relevance this pH area is of particular interest, as the reported blood pH value is 7.4 and the acidic extracellular milieu of solid tumors is often 0.5–1.0 units lower than in healthy tissue.^27^, ^33^ Solutions of all complexes (500 μm in 20 mm phosphate buffer, pH 7.2) and of complexes 3 a*–d*, 4 c*, 5 b*, and 6 c* (500 μm in 20 mm phosphate buffer, pH 5.8, 6.2, 6.7, and 7.9) were incubated at 25 °C over 24 h and periodically analyzed via gradient UHPLC runs on a C18‐RP column. Due to pump pressure fluctuations the retention times tended to shift ±0.18 min. The investigated ruthenium, rhodium and iridium complexes (3 a*–d*, 5 b*, and 6 c*) proved to be stable over a wide range of biologically relevant pH values without any degradation or side‐product formation (Figure 6, Figures S73–S101). This is in accordance to expectations as the experimental conditions in aqueous solution should induce formation of the thiolato‐bridged dimers.

However, in aqueous solution decomposition of the osmium compound 4 c* was observed. At pH 7.2 and 7.9 formation of a second species was observed after 24 h. On the other hand, at pH 5.8, 6.2 and 6.7 complex 4 c* was stable over 24 h, which indicates a pH‐dependent stability in the case of osmium–thiopyridone complexes. In order to elucidate the fate of complex 4 c* and a possible concentration effect on its stability, additional NMR and MS experiments were carried out. NMR experiments at a concentration of 6 mg mL^−1^ in either [D_6_]DMSO or D_2_O indicated no decomposition over 24 h. Therefore, ^1^H NMR kinetics of 4 c* in D_2_O/1 % [D_6_]DMSO (starting concentration 6 mg mL^−1^) were measured with no visible changes over 24 h. Consequently, the sample was diluted 1:1 (3 mg mL^−1^) and kinetics were measured over 24 h a second time. This step was repeated six times in total (after 24, 48, 72, 96, 168, and 192 h; resulting in a final concentration of 176 μm). At a dilution of 375 μg mL^−1^ formation of a second species became apparent, indicated by an additional septet at 2.77 ppm with a 1:4 intensity. Additionally, the formation of a third methyl signal with a 1:1.5 intensity at 1.08 ppm could be observed. It was impossible to measure 2D NMR sets throughout the experiment, due to the low sample concentration. Instead HR ESI‐MS analysis of the 176 μm sample after 168 h was carried out in order to gather further insights into the nature of the newly formed species. Based on the combined findings, we propose that the decomposition of 4 c* is initiated by cleavage of the arene moiety, indicated by the formation of [OsL(CH_3_OH)]^+^ (*m*_exp_=454.0133, *m*_calc_=454.0517), [OsLCl]^−^ (*m*_exp_=454.0176, *m*_calc_=453.9910), [OsL(Cl)(CH_3_O)]^−^ (*m*_exp_=484.0283, *m*_calc_=484.0070), and [OsLCl_2_(CH_3_OH)]^−^ (*m*_exp_=520.0036, *m*_calc_=520.9860) fragments, respectively. These findings suggest a completely different activation mode for osmium(II) thiopyridone complexes where the *p*‐cymene moiety seems to act as the leaving group.

### Investigation of amino acid interaction by HPLC‐MS

**Figure 7:**
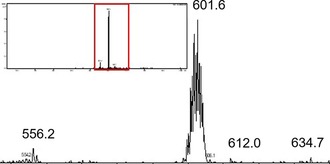
MS spectrum of osmium complex **4 c** after amino acid incubation for 0 h. The formation of a [((*p*‐cym)Os)_2L(_ *N*‐Ac‐Cys)2]^2+^ (*m*_exp_ 601.6, *m*_calc_=601.8) adduct is clearly visible, and metal patterns indicate the presence of a dimeric species.

An important factor for side effects and drug inactivation is the premature interaction of administered anticancer agents with biomolecules on the way to the target site. In order to acquire details about the reactivity of thiopyridone organometallics towards possible binding partners, complexes **3 c**, **4 c**, **5 c**, and **6 c** were incubated in phosphate buffered solutions at pH 7.2 with *N*‐acetyl‐l‐methionine, *N*‐acetyl‐l‐cysteine, and *N*‐acetyl‐l‐histidine (*N*‐Ac‐Met, *N*‐Ac‐Cys, and *N*‐Ac‐His) at 37 °C over 168 h and measured in 24 h intervals via HPLC runs (Figures S102–S105). ESI‐MS analysis revealed *N*‐Ac‐Cys adduct formation for all analyzed complexes, and a *N*‐Ac‐Met adduct with ruthenium complex **3 c** to a lesser extent. All adduct peaks could already be found at 0 h, and HPLC chromatograms and ESI‐MS analyses showed no additional adduct formation over time. In addition ESI‐MS spectra of **3 c** showed peaks with a dimeric metal pattern, according to [((*p*‐cym)Ru)_2_L(*N*‐Ac‐Cys)_2_]^2+^ (*m*_exp_ 513.5, *m*_calc_=513.7) and [((*p*‐cym)Ru)_2_L(*N*‐Ac‐Met)]^+^ (*m*_exp_ 891.1, *m*_calc_=892.1) fragments, while the spectra for osmium complex **4 c** revealed the preferential formation of a [((*p*‐cym)Os)_2_L(*N*‐Ac‐Cys)_2_]^2+^ (*m*_exp_ 601.6, *m*_calc_=601.8) adduct (Figure 7). Furthermore, [((Cp*)Rh)_2_L(*N*‐Ac‐Cys)_2_]^2+^ (*m*_exp_ 515.6, *m*_calc_=514.9) and [((Cp*)Rh)_2_L(*N*‐Ac‐Cys)]^+^ (*m*_exp_ 867.1, *m*_calc_=869.8) fragments could be observed for rhodium complex **5 c**, and iridium complex **6 c** revealed formation of [((Cp*)Ir)_2_L(*N*‐Ac‐Cys)]^2+^ (*m*_exp_ 523.1, *m*_calc_=523.2) and [((Cp*)Ir)_2_L(*N*‐Ac‐Cys)_2_]^2+^ (*m*_exp_ 604.7, *m*_calc_=604.8) adducts, respectively. Overall the [((Ar)M)_2_L(*N*‐Ac‐Cys)_2_]^2+^ species was the most abundant species for all analyzed complexes. On the other hand, no adduct formation with *N*‐Ac‐His was observed. All adducts are based on preceding dissociation of one thiopyridone ligand, which seems to be a crucial step enabling amino acid interaction. These findings indicate a very high affinity of thiopyridone complexes towards sulfur‐containing amino acids rather than nitrogen‐containing amino acids such as l‐histidine under biologically relevant conditions. Furthermore, they are in accordance with previously published results where preferential reactivity of pyrone‐ and pyridone‐based ruthenium(II) complexes towards l‐cysteine and l‐methionine was reported.^34^ Generally, the dimeric complexes of this work showed unexpectedly high affinity towards sulfur‐containing amino acids as adduct formation was observed immediately, and adduct ratios showed only minor changes over time. This behavior strongly deviates from the reactivity of monomeric thiomaltol derivatives, where adduct formation was only observed after 3–24 h, respectively.^18^ These findings prove once more the importance of the attached ligand scaffolds for biological activity and reactivity of piano‐stool complexes, as minor variations thereof may cause major changes in biological studies. Furthermore, it is impossible to do extrapolations between related ligand systems, and in‐depth examination is crucial in order to elucidate a possible mode of action or activation.

### Cytotoxicity

Previously, closely related maltol organometallics had exhibited only moderate activity against the three human cancer cell lines CH1/PA‐1 (ovarian teratocarcinoma), SW480 (colon carcinoma), and A549 (non‐small cell lung carcinoma),^35^ which was subsequently improved via introduction of the *S,O*‐coordination motif, for example, by thiomaltol, resulting in IC_50_ values in the low to sub‐micromolar range.^18^ To expand our knowledge on the cytotoxic behavior of compounds featuring a thiopyridone motif, we now tested ligands **2 a**–**d** and the respective thiopyridone complexes **3 a**–**6 d** in the above mentioned cancer cell lines (Table 2; Figures S108–S111).

**Table 2:** Cytotoxicity of organometallic thiopyridone complexes **3 a**–**6 d**, their thiomaltol analogues and reference drugs.

IC_50_ [μm]^[a]^			
Compound	A549	SW480	CH1/PA‐1
**2 a**	66±6	3.2±0.3	15±1
**2 b**	1.6±1.2^[b]^	0.52±0.03	0.52±0.05
**2 c**	0.79±0.12	0.28±0.04	n/a
**2 d**	1.6±0.1	0.35±0.02	0.62±0.08
**3 a**	>200	55±18	>200
**3 b**	2.4±0.8	2.8±0.1	0.94±0.08
**3 c**	1.3±0.1	3.1±0.2	1.04±0.03
**3 d**	2.1±0.3	4.0±0.3	1.6±0.3
**4 a**	>200	>200	112±27
**4 b**	2.2±1.1	5.8±0.3	1.13±0.03
**4 c**	2.2±1.2	5.6±0.7	1.3±0.2
**4 d**	2.4±0.3	7.7±0.3	1.3±0.3
**5 a**	117±19	5.5±1.1	11±2
**5 b**	2.0±0.8	0.67±0.04	0.66±0.08
**5 c**	0.72±0.11	0.28±0.02	0.37±0.04
**5 d**	1.3±0.2	1.0±0.1	0.48±0.06
**6 a**	63±6	4.2±0.2	7.0±1.6
**6 b**	2.2±0.8	0.59±0.08	0.82±0.05
**6 c**	1.1±0.2	0.54±0.07	0.57±0.05
**6 d**	2.0±0.4	0.80±0.15	0.68±0.02
Ru‐TM	7.7±1.8	4.3±0.2	3.3±0.5
Os‐TM^18^	4.1±0.3	2.0±0.2	2.0±0.2
Rh‐TM^18^	5.9±0.8	1.0±0.1	1.0±0.1
Ir‐TM^18^	5.8±1.7	0.73±0.10	0.57±0.03
Cisplatin^36^	1.3±0.4	3.5±0.3	0.16±0.03
BOLD‐100^18^	156±11	62±9	50±6

[a] 50 % inhibitory concentrations in human carcinoma cell lines A549, SW480, and CH1/PA‐1. Values are means ± SDs obtained by the MTT assay (exposure time: 96 h). [b] High SD due to a broad shoulder at about 50 % in the concentration‐effect curves.

In general, variation of the thiopyridone ligand had a pronounced impact on the cytotoxic activity of these compounds in human cancer cell lines. Complexes featuring the methylamine ligand **2 a** were less active than their respective thiomaltol parent compounds in all tested cell lines by factors of 5 to >100, with IC_50_ values ranging from 4.2 to >200 μm, respectively. Furthermore, the more lipophilic compounds featuring the bulky naphthylamine ligand **2 d** were generally more active (except for osmium compound **4 d** in SW480 cells, which showed four times higher IC_50_ values than the osmium thiomaltol complex). Overall, complexes featuring phenyl and benzyl ligands (**2 b**,**c**) are by far the most active of the established compound library, with IC_50_ values in the low to sub‐micromolar range. Overall, the lowest IC_50_ values were achieved with the Rh‐benzyl compound **5 c**. Apart from the different ligands, variation of the metal center seemed to have a marked impact on cytotoxicity in the tested cancer cell lines. In each ligand series, organo‐osmium derivatives were the least active, followed by the respective Ru analogues, while Rh and Ir complexes showed distinctly higher cytotoxic potency. The most active compound series featured the benzyl ligand **2 c**, where Ru (**3 c**) and Os (**4 c**) analogues had IC_50_ values in the low micromolar range, and their Rh (**5 c**) and Ir (**6 c**) counterparts were found to be even more active with IC_50_ values in the high nanomolar range. Hence, the cytotoxicity of **5 c** and **6 c** is up to 20 times higher than that of **3 c** and **4 c**, depending on the cell line. Overall, Ru and Os organometallics were most active in the broadly chemosensitive CH1/PA‐1 cells, followed by the markedly multidrug‐resistant A549 cells and least active in SW480 cells, which show a certain extent of chemoresistance due to P‐gp expression. Interestingly, the IC_50_ of the complexes featuring either of these two metals was higher by a factor of up to 3 in SW480 cells than in the cell line A549. These findings are in accordance with well‐established Pt^II^ drugs (e.g., cisplatin), which are up to 22 times more active in CH1/PA‐1 cells than in SW480 cells.^36^ Contrary to this, the Rh and Ir complexes turned out to be highly active in SW480 cells, followed by CH1/PA‐1 and least active in A549 cells, where IC_50_ values were up to 21 times higher. This pattern reveals a strong dependency on the employed metal center. In summary, both the ligand and the metal center impact on the anticancer activity in this library of organometallics. Additionally, different trends in cell line sensitivities could be observed depending on the employed metal center.

### Cellular accumulation and lipophilicity

In order to examine possible determinants for the strong differences in cytotoxicity between Ru and Rh congeners, the cellular accumulation of both methyl (**3 a**, **5 a**) and benzyl analogues (**3 c**, **5 c**) was measured in SW480 cells (Table 3). These experiments revealed strong deviations in intracellular metal levels, depending on the ligand, as well as the metal center. Intracellular accumulation was highest for the Rh‐benzyl compound **5 c** (despite a 2.5 times lower concentration applied), followed by its Ru counterpart **3 c**, while complexes featuring a methyl ligand showed lower cellular accumulation overall. These findings are in good accordance with the measured IC_50_ values for these complexes **3 a**, **3 c**, **5 a** and **5 c** in SW480 cells, where benzyl complexes **3 c** and **5 c** exhibited highest cytotoxicity.

**Table 3:** Cellular accumulation in SW480 cells and chromatographic lipophilicity indices of complexes **3 a**, **3 c**, **5 a**, **5 c**, **6 a** and **6 c** in comparison to their IC_50_ values.

Compound	Cellular accumulation [fg/cell]^[a]^	φ_0_	IC_50_ [μm]
**3 a**	21±5	5.51	54.65±18.42
**3 c**	147±54	6.04	3.06±0.2
**5 a**	118±35	5.91	5.54±1.07
**5 c**	855±66	6.28	0.28±0.02
**6 a**	986±152	5.75	4.18±0.19
**6 c**	1680±333	6.22	0.54±0.07

[a] Cellular accumulation in SW480 cells, determined by inductively coupled plasma mass spectrometry (ICP‐MS) after 2 h exposure at concentrations of 20 μm (**5 c**), or 50 μm (**3 a**, **3 c**, **5 a**, **6 a**, **6 c**).

**Figure 8:**
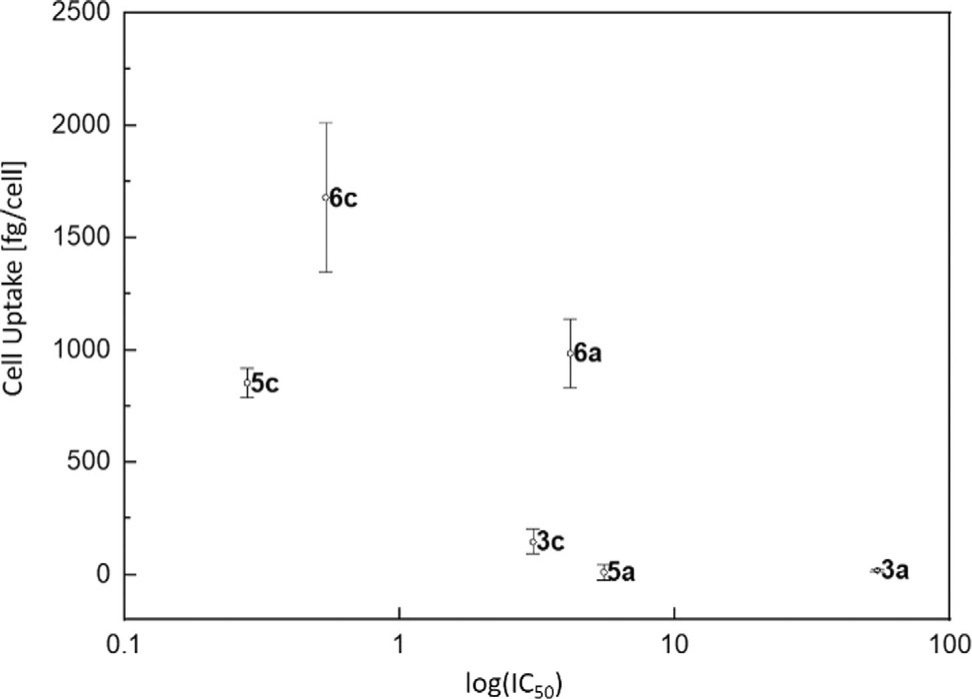
Scatter plot of cellular uptake vs. log(IC_50_) of complexes **3** **a**,**c**, **5** **a**,**c**, **6** **a**, and **6** **c** in SW480 cells. Note that accumulation of **5 c** was determined at a 2.5 times lower concentration.

Additional experiments to determine the chromatographic lipophilicity indices ϕ_0_ were carried out in order to further support these results. Therefore, a well‐established literature‐known procedure was applied, where the retention times of all complexes was compared to a dead time marker in isocratic RP‐UHPLC runs with different mobile phase compositions.^7^, ^37^ In accordance with the commonly employed log*P* value, a higher ϕ_0_ value correlates to a higher lipophilicity of the respective compound.^37^ The ϕ_0_ values for all Ru, Rh, and Ir complexes can be found in the supporting information (Table S36). However, due to stability issues no ϕ_0_ values could be obtained for Os compounds (**4 a**–**d**). According to expectations, complex lipophilicity increases when employing carbon enriched ligands; however, the impact of the applied metal center was marginal. Consequently, the general trend for each transition metal series was, that the naphthyl complexes featuring ligand **d** revealed highest ϕ_0_ values, followed by benzyl complexes **c** and aniline complexes **b**, which showed similar lipophilicity, and the lowest indices were obtained for methyl‐compounds featuring ligand **a**. The findings for methyl (**a**) and benzyl (**c**) complexes establish a good correlation between lipophilicity and cellular accumulation, as well as lipophilicity and cytotoxicity for each metal series (Figure 8, Figures S106–S107). While the Ru‐methyl organometallic **3 a** revealed the lowest lipophilicity index, the lowest cytotoxicity and low cellular accumulation levels, Ru‐benzyl compound **3 c** showed a comparably higher ϕ_0_ value, together with distinctly higher IC_50_ values and intracellular accumulation levels which were seven times as high. The same correlations could be observed for Rh complexes **5 a** and **5 c**, where the cellular accumulation of the benzyl complex was manifold higher than that of the methyl counterpart, even though the applied concentration was lower (**5 a**: 50 μm; **5 c**: 20 μm), associated with IC_50_ values in the nanomolar range and the highest ϕ_0_ value. Finally, Ir‐methyl **6 a** and benzyl **6 c** organometallics revealed the same pattern. Although lipophilicity indices of both Ir compounds were between their respective Ru and Rh counterparts, the cellular accumulation levels were drastically higher. Ir‐methyl compound **6 a** revealed cellular concentrations about 47 times as high as the observed values for Ru‐methyl complex **3 a**, and 82 times as high as Rh‐methyl compound **5 a**. On the other hand, Ir‐benzyl **6 c** concentrations were 11 times as high as Ru‐benzyl **3 c**, and 2 times as high as for Rh‐benzyl **5 c**. While the deviations for benzyl compounds **c** can be explained by the molecular weight of the Ir central ion, which is two times as high as those of Ru and Rh, further studies have to be conducted to completely elucidate the reason for the higher values of methyl compound **6 a**.

### Cell cycle studies

The influence of two ligands, as well as seven complexes on cell cycle distribution was tested in two of the cancer cell lines (SW480, CH1/PA‐1) by means of flow cytometry. The cell cycle distribution (G1, S, G2/M phase in %; Table S37) was investigated upon DNA staining with propidium iodide. Based on their cytotoxic potency, rhodium compounds **5 b** and **5 d** as well as benzyl ligand **2 c** and the corresponding complexes **3 c** (Ru), **5 c** (Rh), and **6 c** (Ir) were chosen and, in order to check for correspondence with cytotoxicity, the least active methyl ligand **2 a** and the respective complexes **5 a** (Rh) and **6 a** (Ir) were included for comparison.

In CH1/PA‐1 cells the benzyl‐featuring ligand **2 c** has little effect on cell cycle distribution, while the corresponding complexes **3 c** (Ru), **5 c** (Rh) and **6 c** (Ir) show increasing activity in the following order: Rh<Ru<Ir (Table S38; 6, 10, 13 % decrease in G1 phase at 2.5 μm, respectively). The most pronounced effect on CH1/PA‐1 cells was observed for Ru‐benzyl complex **3 c** at a concentration of 10 μm, which led to about 16 % decrease in G1 phase in favor of S and G2/M phases. The ligand alone at the same concentration decreased the G1 phase by 9 %. Interestingly, in SW480 cells ligand **2 c** showed an effect at a concentration as low as 2.5 μm (18 % decrease in G1), while complexes **3 c**, **5 c**, and **6 c** exhibited scarce effects at applicable concentrations (<9 % decrease in G1 phase), despite their partially high cytotoxicity.

In the methyl subgroup, featuring ligand **2 a** as well as compounds **5 a** (Rh) and **6 a** (Ir), the metal complexes showed comparable effect on CH1/PA‐1 cells by shifting the cell cycle distribution towards S and G2/M phases by up to 14 % in total (**5 a** at 40 μm) and both were more effective than **2 a**. SW480 cells that were more sensitive than CH1/PA‐1 to higher drug concentrations (cell detachment at concentrations >20 μm was observed) showed minor cell cycle perturbations upon drug exposure. In this case only Rh complex **5 a** caused a noteworthy effect with 10 % decrease in G1 phase at a concentration of 10 μm.

Of the remaining two Rh complexes with an aniline (**5 b**) or naphthyl (**5 d**) ligand, only **5 b** showed a pronounced impact on cell cycle distribution. Moreover, **5 b** is the only compound to cause a G1 phase decrease by more than 20 % in both cell lines (at concentrations of 10 μm in CH1/PA‐1 and 5 μm in SW480).

In conclusion, the compounds show pronounced cytotoxic activity in cancer cells at low to sub‐micromolar concentrations (Table 2), in some cases reversing the activity towards the intrinsically more chemoresistant SW480 cell line, but only some of them (in particular Rh aniline complex **5 b** and benzyl ligand **2 c**) provoked a pronounced cell cycle perturbation upon 24 h exposure. However, even their effects are markedly smaller than those of etoposide, an established drug known to cause cell cycle arrest and hence used as a positive control (Table S38).

### Cytotoxicity in 3D spheroid tumor models

**Figure 9:**
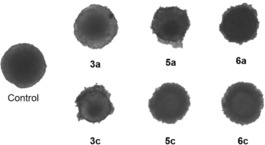
Representative images of HCT‐116 multicellular spheroids after treatment with the indicated compounds (at about their respective IC_50_) after 96 h compared to an untreated control.

Thiopyridone complexes **3 a** (Ru), **5 a** (Rh) and **6 a** (Ir) featuring methyl ligand **2 a** and benzyl derivatives **3 a** (Ru), **5 c** (Rh) and **6 c** (Ir) were also tested in three different human cancer cell lines (A549, HCT‐116, CH1/PA‐1), from which multicellular spheroid models were grown and treated with the desired organometallics for 96 h (Figure 9). As these models mimic solid tumors more closely, it is possible to gather more insights into the cytotoxic behavior of organometallics.^38^ However, the 50 % inhibitory concentrations were higher in 3D models than in 2D monolayer cultures (up to 200 times; values listed in Table 4). This effect has also been reported for other metallodrugs in literature previous publication.^39^ Still, these results confirmed the general cytotoxicity trends of 2D models, where benzyl complexes (**c**) were more active than the respective methyl (**a**) congeners. Additionally, Rh and Ir complexes were least active in the more resistant A549 cell line and showed approximately the same cytotoxicity in CH1/PA‐1 and HCT‐116 cells. The only exception to this trend was Ir benzyl complex **6 c** where a lower concentration was required in HCT‐116 cells for 50 % inhibition compared to both A549 and CH1/PA‐1 cell lines. Interestingly, Ru compounds **3 a** and **3 c** show a different cytotoxicity pattern. While methyl complex **3 a** is distinctly more active in CH1/PA‐1 cells, benzyl‐based complex **3 c** showed similar activity in all three cell lines.

**Table 4:** IC_50_ values of selected compounds in multicellular spheroids of three cell lines after 96 h drug exposure.

IC_50_ [μm]			
Compound	A549	CH1/PA‐1	HCT‐116
**3 a**	>400	267±14	>400
**3 c**	287±3	242±11	211±28
**5 a**	367±24	144±7	140±20
**5 c**	79±5	59±10	62±1
**6 a**	270±17	131±14	138±6
**6 c**	136±5	114±3	78±2

### Laser ablation‐ICP‐MS studies of 3D tumor models

**Figure 10:**
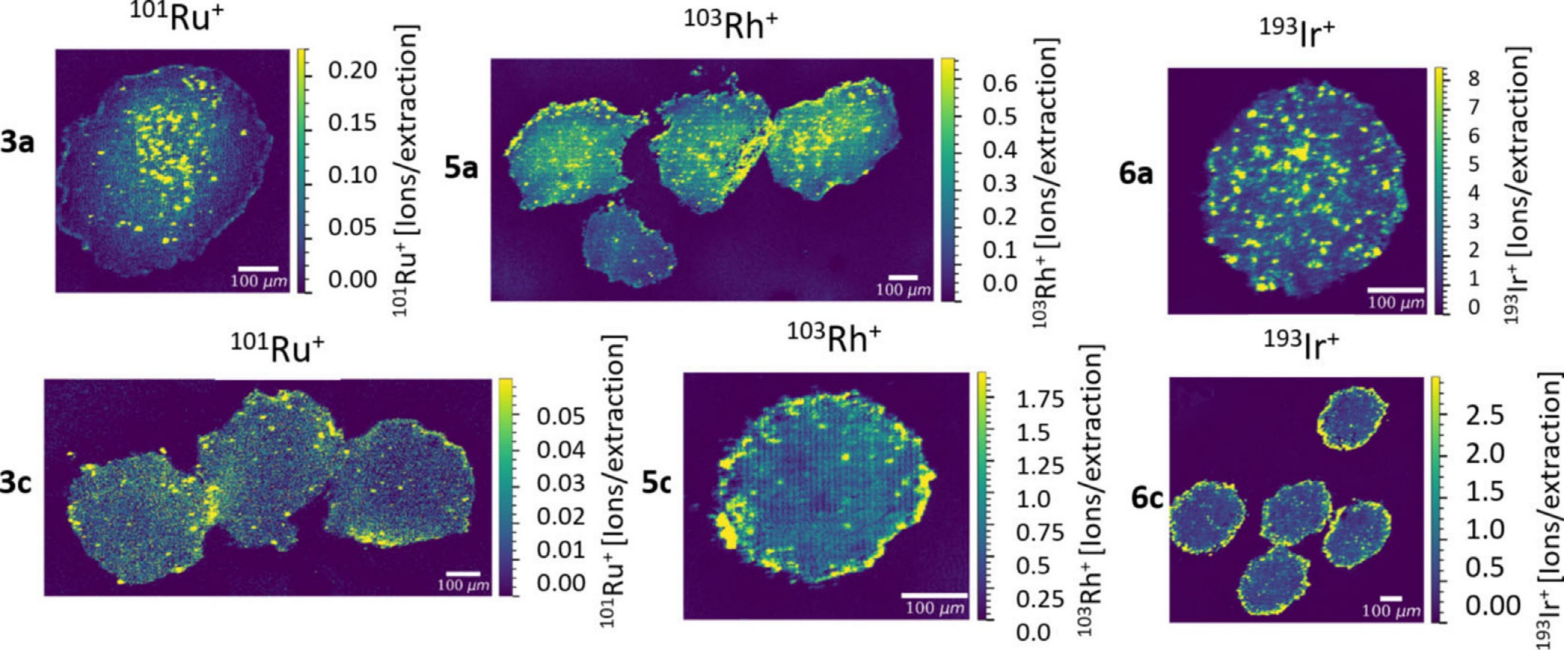
LA‐ICP‐TOF‐MS images of HCT‐116 tumor spheroid sections incubated with organometallic ruthenium (**3** **a**,**c**), rhodium (**5** **a**,**c**), or iridium (**6** **a**,**c**) complexes at the respective IC_50_ concentration for 96 h; scale bar 100 μm.

Laser ablation‐inductively coupled plasma‐mass spectrometry (LA‐ICP‐MS) is a powerful tool to determine the distribution of (metal)drugs in tissue and small organisms (e.g., platinum and ruthenium drugs in vivo).^40^, ^41^, ^42^ This is of special interest as biodistribution in solid tumors is a crucial factor in cancer treatment and thus drug development.^43^ In this work, we report the distribution of ruthenium compounds featuring a methyl or benzyl ligand (**3 a**, **3 c**), as well as the respective rhodium (**5 a**, **5 c**) and iridium (**6 a**, **6 c**) compounds in HCT‐116 (colon cancer) spheroids. The metal content is reported in ions per extraction. Interestingly, the distribution patterns for methyl‐based compounds generally indicate metal disposition throughout the spheroid. This is in accordance with published findings for bioreductive Pt prodrugs, which accumulated in the necrotic core as well.^43^, ^44^ Accordingly, Ru levels of compound **3 a** are highest in the center of the tumor spheroid, while rhodium complex **5 a** accumulated throughout the spheroid sections with higher concentrations at the outer cell layers (proliferating cells), as well as in isolated compartments in the center. On the other hand, iridium organometallic **6 a** revealed an enrichment at the outer rim of the tumor spheroid section (Figure 10). Contrary, complexes featuring a benzyl ligand exhibited highest accumulation levels in the outer cell layers according to literature.^45^ This indicates different disposition of methyl‐ and benzyl‐compounds in biological systems, in accordance with cellular accumulation studies (see section above).

## Conclusions

A library consisting of 16 new thiopyridone‐based piano‐stool complexes was synthesized and characterized by standard analytical methods. These compounds showed increased stability in aqueous solution and higher cytotoxicity compared to their pyridone and thiomaltol parental compounds.^18^, ^19^ These findings could partly be explained by the formation of dimeric species featuring a double positive charge under physiological conditions. It is noteworthy that, despite their structural similarity, the results for osmium compounds deviated in all analytical studies and were not investigated further due to stability issues under physiologically relevant conditions. In order to gather first insights into a possible mode of action as well as SARs, these complexes were investigated by HPLC incubations with *N*‐protected amino acids, revealing a distinct affinity for sulfur‐containing biomolecules (e.g. l‐cysteine). Furthermore, clear trends could be observed in inhibitory concentrations, where benzyl complexes of Rh and Ir proved to be most active, with IC_50_ values in the high nanomolar range. These patterns were paralleled by cellular accumulation experiments, where the most cytotoxic compounds showed the highest cellular accumulation. However, no distinct effect on any cell cycle phase could be observed. Consequently, the mode of action of thiopyridone complexes may significantly differ from that of their thiomaltol congeners, which proved to cause profound S‐phase accumulation.^18^ In order to completely elucidate possible SARs as well as a mode of action further experiments need to be conducted and the thiopyridone library may be expanded.

## Experimental Section

### Materials and methods

All dimeric metal precursors [Ru(*p*‐cym)Cl_2_]_2_,^46^ [Os(*p*‐cym)Cl_2_]_2_,^47^ [Rh(Cp*)Cl_2_]_2_,^48^ and [Ir(Cp*)Cl_2_]_2_,^49^ and ligands **1 a**,^50^ and **2 a**,^51^ were prepared according to literature. The solvents used were purchased from commercial sources and dried before use if needed. Methanol (HPLC grade, Fisher) and DCM (HPLC grade, Fisher) were used for column chromatography. 3‐Hydroxy‐2‐methyl‐4*H*‐pyran‐4‐one (≥99.0 %; maltol), aniline (≥99.0 %), 1‐phenylmethanamine (99 %; benzylamine), hydrazine dihydrochloride (>98.0 %), HCl, and HNO_3_ were purchased from Sigma–Aldrich. Iridium(III) chloride, osmium(VIII) tetroxide, rhodium(III) chloride⋅H_2_O, and ruthenium(III) chloride⋅*x* H_2_O were purchased from Johnson Matthey. Lawesson's reagent (99 %), and hydrochloric acid (37 %), were purchased from Acros‐Fisher. α‐Terpinene (90 %) was purchased from Alfa Aesar. 1,2,3,4,5‐Pentamethylcyclopentadiene (>93 %, TCI Europe), sodium methoxide (ca. 95 %, Fluka), and naphthalene‐1‐amine (>99 %, Merck–Schuchardt) were purchased and used as received. ^1^H, ^13^C (APT) and 2D‐NMR spectra were recorded on a Bruker Avance III^TM^ 500 MHz FT‐NMR spectrometer. ^1^H NMR spectra were measured at 500.10 MHz and ^13^C NMR spectra at 125.75 MHz from solutions in deuterated dimethyl sulfoxide ([D_6_]DMSO) or deuterated water (D_2_O). CHNS elemental analyses were carried out on a Eurovector EA3000 elemental analyzer in the Microanalytical Laboratory of the University of Vienna. High resolution electrospray ionization mass spectra were recorded on a Bruker Maxis UHR qTOF Mass Spectrometer at the Core Facility for Mass Spectrometry of the University of Vienna (Faculty of Chemistry).

The X‐ray intensity data were measured on Bruker D8 Venture and X8 APEX2 diffractometer equipped with multilayer monochromators, Mo_Kα_ INCOATEC micro focus sealed tube and Oxford and Cryoflex2 cooling systems. The structure was solved by direct methods, Patterson or charge flipping and refined by full‐matrix least‐squares techniques. Non‐hydrogen atoms were refined with anisotropic displacement parameters. Hydrogen atoms were inserted at calculated positions and refined with riding model. The following software was used: Bruker SAINT software package^52^ using a narrow‐frame algorithm for frame integration, SADABS^53^ for absorption correction, OLEX2^54^ for structure solution, refinement, molecular diagrams and graphical user‐interface, Shelxle^55^ for refinement and graphical user‐interface SHELXS‐2015^56^ for structure solution, SHELXL‐2015^57^ for refinement, Platon^58^ for symmetry check and π–π interactions proof. Experimental data and CCDC numbers can be found in Table S1 and are available online from http://www.ccdc.cam.ac.uk/.

UV/Vis spectra were recorded using a Hewlett Packard 8452A diode‐array spectrophotometer between 200–800 nm. The path length (*l*) was either 1 or 2 cm. UV/Vis spectra were recorded to confirm complex stability in aqueous systems and over a pH range 5.8–7.9.

Analytical UHPLC was carried out on a Thermo Scientific UltiMate 3000 UHPLC equipped with a silica‐based XBridge C18 (4.6×150 nm, 5 μm) column.

Amino acid incubation studies were performed on an Agilent 1260 Infinity HPLC equipped with a silica‐based Waters Atlantis T3 (4.6×150 nm) column, which was coupled to a Bruker amazon SL ESI‐IT mass spectrometer.

### Stability in aqueous solution

Stock solutions containing the desired complex in DMSO (5 mm) were diluted with phosphate buffer to a final concentration of 50 μm compound in 1 % DMSO/20 mm buffer and directly analyzed via gradient UHPLC runs over 24 h. Phosphate buffers at pH 5.8, 6.2, 6.7, 7.2 and 7.9 were employed to ensure a constant pH value. The instrument was set to 0.6 mL min^−1^ flow rate, 37 °C sampler temperature, 25 °C column compartment, 225 nm detection wavelength, 95 % MeOH in MiliQ water +0.1 % formic acid over 7.0 min.

### Lipophilicity indices

Stock solutions containing the desired complex in DMSO (5 mm) were diluted with phosphate buffer (pH 7.2) to a final concentration of 500 μm compound in 1 % DMSO/20 mm buffer and directly analyzed by four different isocratic UHPLC runs (Δ=5 %). The lipophilicity parameters log*k_w_* and lipophilicity indices φ_0_ were calculated according to literature.^7^, ^37^

### HPLC‐MS incubation studies

For amino acid incubation studies, buffered solutions as described for stability investigations (buffer at pH 7.2) were prepared and additionally contained *N*‐Ac‐Met, *N*‐Ac‐His, and *N*‐Ac‐Cys (500 μm each). The instrument was set to 0.5 mL min^−1^ flow rate, 37 °C sampler temperature, 25 °C column compartment, 225 nm detection wavelength, 95 % MeOH in MiliQ water +0.1 % formic acid over 30.0 min.

### Cell Lines and Culture Conditions

A549 (non‐small cell lung carcinoma), SW480 and HCT‐116 (both colon carcinoma) were kindly provided by Brigitte Marian, Institute of Cancer Research, Department of Medicine I, Medical University of Vienna, Austria. The cell line CH1/PA‐1 (ovarian teratocarcinoma) was kindly provided by Lloyd R. Kelland (CRC Centre for Cancer Therapeutics, Institute of Cancer Research, Sutton, UK). All cell culture media (including supplements) and reagents were obtained from Sigma–Aldrich, and all plasticware from StarLab, unless indicated otherwise.

A549, CH1/PA‐1 and SW480 cells were grown in MEM supplemented with 10 % fetal calf serum (FCS; from Biowest), 1 mm sodium pyruvate, 4 mm l‐glutamine and 1 % v/v nonessential amino acids (from 100×solution) and l‐glutamine. HCT‐116 cells were maintained in McCoy's 5a medium supplemented with 10 % FCS and l‐glutamine. Adherent monolayer cultures were grown in 75 cm^2^ flasks. For spheroid culture, A549, CH1/PA‐1 and HCT‐116 cells were harvested from culture flasks by trypsinization, resuspended in their respective supplemented medium and seeded in ultra‐low attachment round‐bottom 96‐well plates (Corning Costar) at a density of 500 viable cells per well. Plates were incubated for 96 h to allow spheroid formation prior to use for the tests. All cultures were incubated in a humidified incubator at 37 °C with 5 % CO_2_.

### Cytotoxicity tests

Cytotoxicity of the compounds in monolayer cultures was determined by the colorimetric MTT assay (MTT=3‐(4,5‐dimethyl‐2‐thiazolyl)‐2,5‐diphenyl‐2*H*‐tetrazolium bromide). 1×10^3^ CH1/PA‐1, 2×10^3^ SW480 and 3×10^3^ A549 cells were seeded in 100 μL supplemented MEM per well into 96‐well flat‐bottom microculture plates. After 24 h, most test compounds were dissolved in DMSO (Fisher Scientific) first, except for **5 a**, **5 b**, **6 a** and **6 b**, which were dissolved in MEM; all were serially diluted in MEM (to final DMSO contents not exceeding 0.5 % v/v) and added in 100 μL per well. After 96 h, the drug‐containing medium was replaced with 100 μL of RPMI 1640/MTT mixture [6 parts of RPMI 1640 medium (supplemented with 10 % heat‐inactivated fetal bovine serum and 4 mm l‐glutamine), 1 part of MTT solution in phosphate‐buffered saline (5 mg mL^−1^)]. After incubation for 4 h, the mixture was replaced with 150 μL DMSO per well to dissolve the formazan product formed by viable cells. Optical densities at 550 nm (and at a reference wavelength of 690 nm) were measured with a microplate reader (ELx808, Bio‐Tek).

Cytotoxicity in multicellular spheroids was determined by the fluorimetric resazurin assay. For this purpose, 100 μL of the compound dilutions in the appropriate medium (MEM or McCoy's 5a medium) were added to each well of the plates where spheroids had been grown. After incubation for 96 h, 20 μL of a 440 μm resazurin sodium salt solution in PBS were added to each well, and the plates were incubated for further 16 h. Fluorescence was measured with a Synergy HT reader (BioTek) with an excitation wavelength of 530 nm and emission wavelength of 590 nm. For both assays, at least three independent experiments (or two in case of poor activity) were performed, each with triplicates per concentration level, and the 50 % inhibitory concentrations (IC_50_) relative to untreated controls were interpolated from concentration‐effect curves.

### Cell cycle studies

CH1/PA‐1 and SW480 cells were harvested by trypsinization, 8×10^4^ and 1.2×10^5^ cells per well were seeded into 12‐well plates, respectively. The cells were allowed to attach and resume proliferation for 24 h after the seeding. Etoposide was used as a positive control.^59^ Dimethyl sulfoxide (DMSO) or MEM stock solutions of the tested compounds were diluted with MEM and added onto the cells so that the final DMSO content (where applied) did not exceed 0.5 % (controls were treated accordingly). After continuous exposure for 24 h at 37 °C and under 5 % CO_2_, the cells were harvested by trypsinization and centrifuged at 300 g for 3 min. The cells were washed with PBS (1 mL) and resuspended in propidium iodine (PI) containing HSF buffer (600 μL; 0.1 % Triton X‐100, 0.1 % sodium citrate, 50 μg mL^−1^ PI in PBS). After incubation overnight at 4 °C in the dark, 5×10^3^ cells were measured by flow cytometry with a Millipore Guava easyCyte 8HT instrument (Luminex, USA). Data were evaluated by means of FlowJo software (Tree Star) using Watson Pragmatic algorithms.^60^ The fitting of the curve was chosen to keep the root mean square error between 1.0 and 2.0, alongside with good fitting visual model.

### LA‐ICP‐TOF‐MS imaging

An Analyte Excite Excimer 193 nm laser ablation system (Teledyne Photon Machines, Bozeman, MT, USA), equipped with a prototype Cobalt ablation cell and the aerosol rapid introduction system (ARIS), was coupled to an *icp*TOF 2R ICP‐TOF‐Ms instrument (TOFWERK AG, Thun, Switzerland). The optimized He carrier gas flow was 0.5 L min^−1^, to which Ar was added as a makeup gas (≈0.9 L min^−1^) through the low dispersion mixing bulb of the ARIS before entering the plasma. The performance and settings of the instruments were optimized on a daily basis using the standard reference material NIST SRM612 (National Institute for Standards and Technology, Gaithersburg, MD, USA). Optimization criteria are the signal intensities of the isotopes ^59^Co^+^, ^115^In^+^ and ^238^U^+^ as well as the ratios of ^238^U^16^O^+^ to ^238^U^+^ (oxide formation, <2 %) and ^238^U^+^ to ^232^Th^+^ (elemental fractionation, ca. 1). The specified mass resolving power of the ICP‐TOF‐MS is *m/Δm*=6000 (FWHM definition) and the analyzable mass range in the used operation mode is *m*/*z=*14–256. The plasma Ar gas flow was 14 L min^−1^, the auxiliary Ar gas flow was ≈0.8 L min^−1^ and the radio frequency power was 1440 W. Laser ablation was performed with a square spot with 5 μm in diameter and a repetition rate of 200 Hz. The fixed dosage mode was used with dosage set to 2 and the shift between lines was 2.5 μm resulting in an effective ablation area of 2.5×2.5 μm. Tumor spheroid sections were removed quantitatively with a laser fluorescence of 0.75 J cm^−2^. Data was recorded using TofPilot 1.3.4.0 (TOFWERK AG, Thun, Switzerland). LA‐ICP‐TOF‐MS data was further processed with HDIP version 1.2.5.beta4 (Teledyne Photon Machines, Bozeman, MT, USA).

### General procedures

**General protocol for the syntheses of Os^II^/Ru^II^** ***p*** ‐cymene and Ir^III^/Rh^III^ Cp* halido complexes: Syntheses of all complexes were performed by dissolving the respective ligand (1 equiv.) and sodium methoxide (1.1 equiv.) in absolute methanol (10 mL) and the solution was stirred under Ar atmosphere at RT for 20 min. The respective dimeric metal precursor (0.9 equiv.) was added and the resulting dark colored mixture was stirred at RT or 40 °C for several hours (0.5–3.5 h depending on the complex). Afterwards, the solvent was concentrated under reduced pressure and the crude product was dissolved in dichloromethane. In order to remove insoluble by‐products, the solution was filtrated and the filtrate was concentrated in vacuo. Precipitation or crystallization from DCM/Et_2_O or DCM/*n*‐hexane afforded the desired products in moderate to good yields (33–83 %).

**Chlorido[1,2‐dimethyl‐3‐oxo‐κ*O*‐pyridine‐4(1*H*)‐thionato‐κ*S*](η^6^‐*p*‐cymene)‐ruthenium(II) (3 a)**: The synthesis was performed according to the general complexation protocol using ligand **2 a** (101 mg, 0.653 mmol,), sodium methoxide (39 mg, 0.719 mmol) and bis[dichlorido(η^6^‐*p*‐cymene)ruthenium(II)] (200 mg, 0.327 mmol) and a reaction time of 1.5 h. The product was precipitated from DCM/Et_2_O and isolated as a red powder. Yield: 176 mg (63 %).

Monomer **3 a**: ^1^H NMR (500.10 MHz, [D_6_]DMSO, 25 °C): *δ*=1.21 (d, ^3^ *J* (H,H)=7 Hz, 6 H, Hg), 2.14 (s, 3 H, H1), 2.44 (s, 3 H, Ha), 2.67–2.77 (m, 1 H, Hf), 3.86 (s, 3 H, H7), 5.85 (d, ^3^ *J* (H,H)=6 Hz, 2 H, Hd), 6.01 (d, ^3^ *J* (H,H)=6 Hz, 2 H, Hc), 7.27 (d, ^3^ *J* (H,H)=6 Hz, 1 H, H5), 7.54 (d, ^3^ *J* (H,H)=6 Hz, 1 H, H6) ppm. ^13^C NMR (125.75 MHz, [D_6_]DMSO, 25 °C): *δ*=11.9 (C1), 17.5 (Ca), 22.1 (Cg), 30.4 (Cf), 43.3 (C7), 87.2 (Cd), 89.0 (Cc), 102.6 (Ce), 106.6 (Cb), 120.8 (C5), 126.1 (C6), 137.7 (C2), 159.1 (C4), 166.8 (C3) ppm.

Dimer 3 a*: ^1^H NMR (500.10 MHz, D_2_O, 25 °C): *δ*=0.87 (d, ^3^ *J* (H,H)=7 Hz, 6 H, Hg), 1.04 (d, ^3^ *J*(H,H)=7 Hz, 6 H, Hg), 2.05 (s, 6 H, H1), 2.21 (s, 6 H, Ha), 2.53–2.62 (m, 2 H, Hf), 3.81 (s, 6 H, H7), 5.38 (d, ^3^ *J* (H,H)=6 Hz, 2 H, Hd), 5.42 (d, ^3^ *J* (H,H)=6 Hz, 2 H, Hc), 5.60 (d, ^3^ *J* (H,H)=6 Hz, 2 H, Hd), 5.82 (d, ^3^ *J* (H,H)=6 Hz, 2 H, Hc), 7.26 (d, ^3^ *J* (H,H)=6 Hz, 2 H, H5), 7.35 (d, ^3^ *J* (H,H)=6 Hz, 2 H, H6) ppm; ^13^C NMR (125.75 MHz, D_2_O, 25 °C): *δ*=12.3 (C1), 17.6 (Ca), 20.7 (Cg), 21.7 (Cg), 30.1 (Cf), 44.7 (C7), 80.3 (Cc), 83.9 (Cc), 84.9 (Cd), 86.2 (Cd), 103.2 (Ce), 106.9 (Cb), 126.6 (C5), 129.4 (C6), 141.6 (C2), 146.1 (C4), 171.3 (C3) ppm.

ESI‐HR‐MS^+^ *m*/*z* found (calculated): [*M*]^+^ 390.0460 (390.0464). Elemental analysis calcd (%) for C_17_H_22_ClNORuS⋅1.25 H_2_O: C 45.63, H 5.52, N 3.13, S 7.17; found: C 45.60, H 5.55, N 3.09, S 7.07.

**Chlorido[2‐methyl‐3‐(oxo‐κ*O*)‐1‐phenylpyridine‐4(1*H*)‐thionato‐κ*S*](η^6^‐*p*‐cymene)ruthenium(II) (3 b)**: The synthesis was performed according to the general complexation protocol using ligand **2 b** (142 mg, 0.653 mmol), sodium methoxide (39 mg, 0.719 mmol) and bis[dichlorido(η^6^‐*p*‐cymene)ruthenium(II)] (200 mg, 0.327 mmol) and a reaction time of 1.5 h. The product was crystallized from DCM/Et_2_O and isolated as red crystals. Yield: 205 mg (65 %).

Monomer **3 b**: ^1^H NMR (500.10 MHz, [D_6_]DMSO, 25 °C): *δ*=1.22 (d, ^3^ *J* (H,H)=7 Hz, 6 H, Hg), 2.11 (s, 3 H, H1), 2.14 (s, 3 H, Ha), 2.73 (sept, ^3^ *J* (H,H)=7 Hz, 1 H, Hf), 5.84 (d, ^3^ *J* (H,H)=6 Hz, 2 H, Hd), 6.01 (d, ^3^ *J* (H,H)=6 Hz, 2 H, Hc), 7.36 (d, ^3^ *J* (H,H)=6 Hz, 1 H, H5), 7.48–7.54 (m, 3 H, H6, H8, H12), 7.59–7.64 (m, 3 H, H9, H10, H11) ppm; ^13^C NMR (125.75 MHz, [D_6_]DMSO, 25 °C): *δ*=13.9 (C1), 17.5 (Ca), 22.2 (Cg), 30.4 (Cf), 85.4 (Cd), 88.9 (Cc), 102.4 (Ce), 105.8 (Cb), 120.8 (C5), 126.0 (C8, C12), 128.8 (C6), 129.9 (C9, C11), 130.1 (C10), 134.1 (C2), 141.3 (C7), 162.1 (C4), 166.7 (C3) ppm.

Dimer 3 b*: ^1^H NMR (500.10 MHz, D_2_O, 25 °C): *δ*=1.03 (d, ^3^ *J* (H,H)=7 Hz, 6 H, Hg), 1.18 (d, ^3^ *J* (H,H)=7 Hz, 6 H, Hg), 2.05 (s, 6 H, H1), 2.33 (s, 6 H, Ha), 2.68–2.78 (m, 2 H, Hf), 5.56 (d, ^3^ *J* (H,H)=6 Hz, 2 H, Hd), 5.61 (d, ^3^ *J* (H,H)=6 Hz, 2 H, Hc), 5.76 (d, ^3^ *J* (H,H)=6 Hz, 2 H, Hc), 5.98 (d, ^3^ *J* (H,H)=6 Hz, 2 H, Hd), 7.06 (d, ^3^ *J* (H,H)=8 Hz, 2 H, H8, H12), 7.32 (d, ^3^ *J* (H,H)=8 Hz, 2 H, H8, H12), 7.50–7.57 (m, 6 H, H5, H6, H9, H11), 7.59 (dd, ^3^ *J* (H,H)=8 Hz, ^3^ *J* (H,H)=8 Hz, 2 H, H9, H11), 7.68–2.78 (m, 2 H, H10) ppm; ^13^C NMR (125.75 MHz, D_2_O, 25 °C): *δ*=14.8 (C1), 17.6 (Ca), 20.8 (Cg), 21.8 (Cg), 30.1 (Cf), 80.7 (Cd), 84.0 (Cc), 85.2 (Cd), 86.0 (Cc), 103.2 (Ce), 107.1 (Cb) 124.3 (C8, C12), 125.6 (C8, C12), 126.4 (C5), 129.3 (C6), 130.2 (C9, C11), 130.3 (C9, C11), 131.0 (C10), 140.5 (C7), 140.9 (C2), 148.7 (C4), 171.6 (C3) ppm.

ESI‐HR‐MS^+^ *m*/*z* found (calculated): [*M*]^+^ 538.0158 (538.0158). Elemental analysis calcd (%) for C_22_H_24_ClNORuS: C 54.26, H 4.97, N 2.88, S 6.58; found: C 54.26, H 4.98, N 2.90, S 5.60.

**Chlorido[1‐benzyl‐2‐methyl‐3‐(oxo‐κ*O*)‐pyridine‐4(1*H*)‐thionato‐κ*S*](η^6^‐*p*‐cymene)ruthenium(II) (3 c)**: The synthesis was performed according to the general complexation protocol using ligand **2 c** (227 mg, 0.980 mmol), sodium methoxide (58 mg, 1.078 mmol) and bis[dichlorido(η^6^‐*p*‐cymene)ruthenium(II)] (300 mg, 0.490 mmol) and a reaction time of 1 h. The product was crystallized from DCM/Et_2_O and isolated as red crystals. Yield: 163 mg (33 %).

Monomer **3 c**: ^1^H NMR (500.10 MHz, [D_6_]DMSO, 25 °C): *δ*=1.18 (d, ^3^ *J* (H,H)=7 Hz, 6 H, Hg), 2.12 (s, 3 H, Ha), 2.33 (s, 3 H, H1), 2.68 (sept, ^3^ *J* (H,H)=7 Hz, 1 H, Hf), 5.50 (s, 2 H, H7), 5.83 (d, ^3^ *J* (H,H)=6 Hz, 2 H, Hd), 5.98 (d, ^3^ *J* (H,H)=6 Hz, 2 H, Hc), 7.10 (d, ^3^ *J* (H,H)=7 Hz, 2 H, H9, H13), 7.31–7.36 (m, 1 H, H5), 7.36–7.42 (m, 2 H, H10, H12), 7.46 (d, ^3^ *J* (H,H)=6 Hz, 1 H, H11), 7.73 (d, ^3^ *J* (H,H)=6 Hz, 1 H, H6) ppm; ^13^C NMR (125.75 MHz, [D_6_]DMSO, 25 °C): *δ*=12.1 (C1), 17.5 (Ca), 22.4 (Cg), 30.4 (Cf), 58.1 (C7), 78.9 (Cd), 81.1 (Cc), 94.5 (Ce), 98.7 (Cb), 121.0 (C5), 126.7 (C9, C13), 128.3 (C11), 129.1 (C10, C12), 130.4 (C6), 135.0 (C8), 136.5 (C2), 161.2 (C4), 169.0 (C3) ppm.

Dimer 3 c*: ^1^H NMR (500.10 MHz, D_2_O, 25 °C): *δ*=0.88 (d, ^3^ *J* (H,H)=7 Hz, 6 H, Hg), 0.98 (d, ^3^ *J* (H,H)=7 Hz, 6 H, Hg), 1.57 (s, 6 H, H1), 2.16 (s, 6 H, Ha), 2.48–2.56 (m, 2 H, Hf), 5.05 (d, ^2^ *J* (H,H)=15 Hz, 2 H, H7a), 5.13 (d, ^2^ *J* (H,H)=15 Hz, 2 H, H7b), 5.40 (d, ^3^ *J* (H,H)=6 Hz, 2 H, Hd), 5.45 (d, ^3^ *J* (H,H)=7 Hz, 2 H, Hc), 5.50 (d, ^3^ *J* (H,H)=7 Hz, 2 H, Hd), 5.66 (d, ^3^ *J* (H,H)=7 Hz, 2 H, Hc), 7.09 (dd, ^3^ *J* (H,H)=8 Hz, ^4^ *J* (H,H)=2 Hz, 4 H, H9, H13), 7.33–7.40 (m, 6 H, H10, H11, H12), 7.56 (d, ^3^ *J* (H,H)=6 Hz, 2 H, H5), 7.60 (d, ^3^ *J* (H,H)=6 Hz, 2 H, H6) ppm; ^13^C NMR (125.75 MHz, D_2_O, 25 °C): *δ*=12.3 (C1), 17.5 (Ca), 21.1 (Cg), 21.9 (Cg), 30.2 (Cf), 60.1 (C7), 73.0 (Cc), 76.5 (Cc), 76.9 (Cd), 79.0 (Cd), 95.1 (Ce), 99.0 (Cb), 125.1 (C5), 127.7 (C9, C13), 129.3 (C6), 129.5 (C10, C12), 130.3 (C11), 132.7 (C8), 141.4 (C2), 146.4 (C4), 172.7 (C3) ppm.

ESI‐HR‐MS^+^ *m*/*z* found (calculated): [*M*]^+^ 452.0626 (452.0622). Elemental analysis calcd (%) for C_23_H_26_ClNORuS⋅0.5 H_2_O: C 54.16, H 5.34, N 2.75, S 6.29; found: C 54.04, H 5.36, N 2.78, S 6.39.

**Chlorido[2‐methyl‐1‐(naphthalene‐1‐yl)‐3‐(oxo‐κ*O*)‐pyridine‐4(1*H*)‐thionato‐κ*S*](η^6^‐*p*‐cymene)ruthenium(II) (3 d)**: The synthesis was performed according to the general complexation protocol using **2 d** (174 mg, 0.653 mmol), sodium methoxide (39 mg, 0.719 mmol) and bis[dichlorido(η^6^‐*p*‐cymene)ruthenium(II)] (200 mg, 0.327 mmol) and a reaction time of 1 h. The product was crystallized from DCM/Et_2_O and isolated as red crystals. Yield: 248 mg (63 %).

^1^H NMR (500.10 MHz, [D_6_]DMSO, 25 °C): *δ*=1.24 (d, ^3^ *J* (H,H)=7 Hz, 6 H, Hg), 1.98 (s, 3 H, H1), 2.17 (s, 3 H, Ha), 2.75 (sept., ^3^ *J* (H,H)=7 Hz, 1 H, Hf), 5.84–5.95 (m, 2 H, Hd), 6.05 (s, 2 H, Hc), 7.01 (d, ^3^ *J* (H,H)=6 Hz, 1 H, H5), 7.47 (d, ^3^ *J* (H,H)=8 Hz, 1 H, H8), 7.60–7.66 (m, 2 H, H9, H14), 7.71–7.66 (m, 3 H, H6, H10, H13), 8.17 (d, ^3^ *J* (H,H)=8 Hz, 1 H, H15), 8.24 (d, ^3^ *J* (H,H)=8 Hz, 1 H, H12) ppm; ^13^C NMR (125.75 MHz, [D_6_]DMSO, 25 °C): *δ*=13.1 (C1), 17.5 (Ca), 22.3 (Cg), 22.6 (Cg), 30.5 (Cf), 79.0 (Cc), 81.4 (Cd), 103.7 (Ce), 106.1 (Cb), 120.7 (C5), 121.2 (C15), 124.7 (C8), 125.8 (C9/C13), 126.1 (C9/C13), 127.5 (C6), 127.7 (C11, C16), 128.8 (C12, C14), 129.8 (C5), 130.7 (C10), 133.7 (C16), 137.0 (C2, C7), 167.0 (C3, C4) ppm.

ESI‐HR‐MS^+^ *m*/*z* found (calculated): [*M*]^+^ 572.0163 (572.0162). Elemental analysis calcd (%) for C_26_H_26_ClNORuS⋅0.5 CH_2_Cl_2_: C 54.92, H 4.70, N 2.42, S 5.53; found: C 54.88, H 4.59, N 2.54, S 5.44.

**[Chlorido[1,2‐dimethyl‐3‐(oxo‐κ*O*)‐pyridine‐4(1*H*)‐thionato‐κ*S*](η^6^‐*p*‐cymene)osmium(II)] (4 a)**: The synthesis was performed according to the general complexation protocol using **2 a** (70 mg, 0.448 mmol), sodium methoxide (27 mg, 0.493 mmol) and bis[dichlorido(η^6^‐*p*‐cymene)osmium(II)] (177 mg, 0.224 mmol) and a reaction time of 1.5 h. The product was crystallized from DCM/Et_2_O and isolated as orange crystals. Yield: 130 mg (56 %).

Monomer **4 a**: ^1^H NMR (500.10 MHz, [D_6_]DMSO, 25 °C): *δ*=1.22 (d, ^3^ *J*(H,H)=7 Hz, 6 H; Hg), 2.22 (s, 3 H; Ha), 2.48 (s, 3 H, H1), 2.62–2.70 (m, 1 H, Hf), 3.89 (s, 3 H, H7), 5.93 (d, ^3^ *J* (H,H)=5 Hz, 2 H, Hd), 6.10 (d, ^3^ *J* (H,H)=5 Hz, 2 H, Hc), 7.34 (d, ^3^ *J* (H,H)=6 Hz, 1 H, H6), 7.61 (d, ^3^ *J* (H,H)=6 Hz, 1 H, H5) ppm; ^13^C NMR (125.75 MHz, [D_6_]DMSO, 25 °C): *δ*=12.0 (C1), 17.4 (Ca), 22.4 (Cg), 30.5 (Cf), 43.4 (C7), 78.8 (Cc), 81.3 (Cd), 95.1 (Cb), 97.1 (Ce), 120.6 (C5), 130.2 (C6), 137.4 (C2), 159.6 (C4), 168.3 (C3) ppm.

Dimer 4 a*: ^1^H NMR (500.10 MHz, D_2_O, 25 °C): *δ*=0.94 (d, ^3^ *J* (H,H)=6 Hz, 6 H, Hg), 1.15 (d, ^3^ *J* (H,H)=6 Hz, 6 H, Hg), 2.24 (s, 6 H, H1), 2.38 (s, 6 H, Ha), 2.55–2.64 (m, 2 H, Hf), 3.92 (s, 6 H, H7), 5.76 (dd, ^3^ *J* (H,H)=6 Hz, ^3^ *J* (H,H)=6 Hz, 4 H, Hc, Hd), 5.96 (d, ^3^ *J* (H,H)=6 Hz, 2 H, Hd), 6.16 (d, ^3^ *J* (H,H)=6 Hz, 2 H, Hc), 7.47 (dd, ^3^ *J* (H,H)=6 Hz, ^3^ *J* (H,H)=6 Hz, 4 H, H5, H6) ppm; ^13^C NMR (125.75 MHz, 298.2 K, D_2_O): *δ*=12.5 (C1), 17.6 (Ca), 21.1 (Cg), 22.1 (Cg), 30.2 (Cf), 44.8 (C7), 72.3 (Cc), 75.5 (Cd), 76.3 (Cc), 78.8 (Cd), 96.5 (Cb), 99.2 (Ce), 126.5 (C5), 130.2 (C6), 142.3 (C2), 145.3 (C4), 172.5 (C3) ppm.

ESI‐HR‐MS^+^ *m*/*z* found (calculated): [*M*]^+^ 550.0398 (550.0398). Elemental analysis calcd (%) for C_17_H_22_ClNOOsS: C 39.72, H 4.31, N 2.72, S 6.24; found: C 39.62, H 4.20, N 2.80, S 6.19.

**Chlorido[2‐methyl‐3‐(oxo‐κ*O*)‐1‐phenylpyridine‐4(1*H*)‐thionato‐κ*S*](η^6^‐*p*‐cymene)osmium(II) (4 b)**: The synthesis was performed according to the general complexation protocol using **2 b** (137 mg, 0.632 mmol), sodium methoxide (38 mg, 0.696 mmol) and bis[dichlorido(η^6^‐*p*‐cymene)osmium(II)] (250 mg, 0.316 mmol) and a reaction time of 1.5 h. The product was crystallized from DCM/Et_2_O and isolated as orange crystals. Yield: 213 mg (58 %).

Monomer **4 b**: ^1^H NMR (500.10 MHz, [D_6_]DMSO, 25 °C): *δ*=1.24 (d, ^3^ *J*(H,H)=7 Hz, 6 H; Hg), 2.16 (s, 3 H, H1), 2.22 (s, 3 H, Ha), 2.69 (sept., ^3^ *J* (H,H)=7 Hz, 1 H, Hf), 5.96 (d, ^3^ *J* (H,H)=5 Hz, 2 H, Hd), 6.15 (d, ^3^ *J* (H,H)=7 Hz, 2 H, Hc), 7.46 (d, ^3^ *J* (H,H)=6 Hz, 1 H, H5), 7.51–7.56 (m, 2 H, H8, H12), 7.59 (d, ^3^ *J* (H,H)=7 Hz, 1 H, H6), 7.60–7.66 (m, 3 H, H9, H10, H11) ppm; ^13^C NMR (125.75 MHz, [D_6_]DMSO, 25 °C): *δ*=13.9 (C1), 17.4 (Ca), 22.5 (Cg), 30.5 (Cf), 78.5 (Cd), 81.3 (Cc), 95.7 (Ce), 98.2 (Cb), 120.7 (C5), 126.1 (C8, C12), 129.9 (C9, C11), 130.0 (C10), 130.2 (C6), 136.3 (C2), 141.1 (C7), 162.4 (C4), 168.3 (C3) ppm.

Dimer 4 b*: ^1^H NMR (500.10 MHz, D_2_O, 25 °C): *δ*=1.01 (d, ^3^ *J* (H,H)=7 Hz, 6 H, Hg), 1.19 (d, ^3^ *J* (H,H)=7 Hz, 6 H, Hg), 2.15 (s, 6 H, H1), 2.42 (s, 6 H, Ha), 2.66 (sept, ^3^ *J* (H,H)=7 Hz, 2 H, Hf), 5.86 (d, ^3^ *J* (H,H)=6 Hz, 2 H, Hd), 5.91 (d, ^3^ *J* (H,H)=6 Hz, 2 H, Hc), 6.05 (d, ^3^ *J* (H,H)=6 Hz, 2 H, Hc), 6.25 (d, ^3^ *J* (H,H)=6 Hz, 2 H, Hd), 7.06 (d, ^3^ *J* (H,H)=8 Hz, 2 H, H8/H12), 7.33 (d, ^3^ *J* (H,H)=8 Hz, 2 H, H8/H12), 7.51–7.64 (m, 6 H, H6, H9, H11), 7.64–7.71 (m, 4 H, H5, H10) ppm; ^13^C NMR (125.75 MHz, D_2_O, 25 °C): *δ*=14.9 (C1), 17.6 (Ca), 21.1 (Cg), 22.1 (Cg), 30.2 (Cf), 72.6 (Cc), 75.6 (Cd), 76.8 (Cc), 78.9 (Cd), 96.5 (Ce), 99.6 (Cb), 124.3 (C8, C12), 125.6 (C8, C12), 126.2 (C5), 130.1 (C9, C11), 130.3 (C6), 131.1 (C10), 140.4 (C7), 141.8 (C2), 147.8 (C4), 172.7 (C3) ppm.

ESI‐HR‐MS^+^ *m*/*z* found (calculated): [*M*]^+^ 612.0555 (612.0555). Elemental analysis calcd (%) for C_22_H_24_ClNOOsS: C 45.86, H 4.20, N 2.43, S 5.56; found: C 45.93, H 4.19, N 2.59, S 5.54.

**Chlorido[1‐benzyl‐2‐methyl‐3‐(oxo‐κ*O*)‐pyridine‐4(1*H*)‐thionato‐κ*S*](η^6^‐*p*‐cymene)osmium(II) (4 c)**: The synthesis was performed according to the general complexation protocol using **2 c** (146 mg, 0.632 mmol), sodium methoxide (38 mg, 0.696 mmol) and bis[dichlorido(η^6^‐*p*‐cymene)osmium(II)] (250 mg, 0.316 mmol) and a reaction time of 1.5 h. The product was crystallized from DCM/Et_2_O and isolated as orange crystals. Yield: 171 mg (46 %).

Monomer **4 c**: ^1^H NMR (500.10 MHz, [D_6_]DMSO, 25 °C): *δ*=1.19 (d, ^3^ *J* (H,H)=7 Hz, 6 H, Hg), 2.20 (s, 3 H, Ha), 2.37 (s, 3 H, H1), 2.60–2.69 (m, 1 H, Hf), 5.55 (s, 2 H, H7), 5.94 (d, ^3^ *J* (H,H)=6 Hz, 2 H, Hc), 6.10 (d, ^3^ *J*(H,H)=6 Hz, 2 H, Hd), 7.11 (d, ^3^ *J* (H,H)=7 Hz, 2 H, H9, H13), 7.34 (dd, ^3^ *J* (H,H)=7 Hz, ^3^ *J* (H,H)=7 Hz, 1 H, H11), 7.40 (dd, ^3^ *J* (H,H)=7 Hz, ^3^ *J*(H,H)=7 Hz, 2 H, H10, H12), 7.46 (d, ^3^ *J* (H,H)=6 Hz, 1 H, H5), 7.79 (d, ^3^ *J* (H,H)=6 Hz, 1 H, H6) ppm; ^13^C NMR (125.75 MHz, [D_6_]DMSO, 25 °C): *δ*=12.1 (C1), 17.5 (Ca), 22.4 (Cg), 30.4 (Cf), 58.1 (C7), 78.9 (Cc), 81.1 (Cd), 94.5 (Cb), 98.7 (Ce), 121.0 (C5), 126.7 (C9, C13), 128.3 (C11), 129.1 (C10, C12), 130.4 (C6), 135.0 (C8), 136.5 (C2), 161.2 (C4), 169.0 (C3) ppm.

Dimer 4 c*: ^1^H NMR (500.10 MHz, D_2_O, 25 °C): *δ*=0.95 (d, ^3^ *J* (H,H)=7 Hz, 6 H, Hg), 1.09 (d, ^3^ *J* (H,H)=7 Hz, 6 H, Hg), 1.81 (s, 6 H, H1), 2.32 (s, 6 H, Ha), 2.54 (sept., ^3^ *J* (H,H)=7 Hz, 2 H, Hf), 5.09 (d, ^2^ *J* (H,H)=15 Hz, 2 H, H7), 5.23 (d, ^2^ *J* (H,H)=15 Hz, 2 H, H7), 5.82 (d, ^3^ *J* (H,H)=6 Hz, 2 H, Hd), 5.86 (d, ^3^ *J* (H,H)=6 Hz, 2 H, Hc), 5.91 (d, ^3^ *J* (H,H)=6 Hz, 2 H, Hc), 6.08 (d, ^3^ *J* (H,H)=6 Hz, 2 H, Hd), 7.17–7.22 (m, 4 H, H9, H13), 7.45–7.49 (m, 6 H, H10, H11, H12), 7.56 (d, ^3^ *J* (H,H)=6 Hz, 2 H, H5), 7.74 (d, ^3^ *J* (H,H)=6 Hz, 2 H, H6) ppm; ^13^C NMR (125.75 MHz, D_2_O, 25 °C): *δ*=12.3 (C1), 17.5 (Ca), 21.1 (Cg), 21.9 (Cg), 30.2 (Cf), 60.1 (C7), 73.0 (Cc), 76.5 (Cd), 76.9 (Cc), 79.0 (Cd), 95.1 (Cb), 99.0 (Ce), 125.1 (C5), 127.7 (C9, C13), 129.3 (C11), 129.5 (C10, C12), 130.3 (C6), 132.6 (C8), 141.4 (C2), 146.4 (C4), 172.7 (C3) ppm.

ESI‐HR‐MS^+^ *m*/*z* found (calculated): [*M*]^+^ 626.0714 (626.0711). Elemental analysis calcd (%) for C_23_H_26_ClNOOsS⋅0.5 CH_2_Cl_2_: C 44.61, H 4.30, N 2.21, S 5.07; found: C 44.54, H 4.25, N 2.25, S 4.92.

**Chlorido[2‐methyl‐1‐(naphthalene‐1‐yl)‐3‐(oxo‐κ*O*)‐pyridine‐4(1*H*)‐thionato‐κ*S*](η^6^‐*p*‐cymene)osmium(II) (4 d)**: The synthesis was performed according to the general complexation protocol using **2 d** (168 mg, 0.628 mmol), sodium methoxide (37 mg, 0.691 mmol) and bis[dichlorido(η^6^‐*p*‐cymene)osmium(II)] (248 mg, 0.314 mmol) and a reaction time of 1 h. The product was crystallized from DCM/Et_2_O and isolated as orange crystals. Yield: 248 mg (63 %).

Monomer **4 d**: ^1^H NMR (500.10 MHz, [D_6_]DMSO, 25 °C): *δ*=1.24 (d, ^3^ *J* (H,H)=7 Hz, 6 H, Hg), 2.02 (s, 3 H, H1), 2.24 (s, 3 H, Ha), 2.66–2.74 (m, 1 H, Hf), 5.98 (d, ^3^ *J* (H,H)=5 Hz, 1 H, Hd), 6.02 (d, ^3^ *J* (H,H)=5 Hz, 1 H, Hc), 6.16 (d, ^3^ *J* (H,H)=5 Hz, 2 H, Hc, Hd), 7.04 (d, ^3^ *J* (H,H)=6 Hz, 1 H, H5), 7.55 (d, ^3^ *J* (H,H)=8 Hz, 1 H, H8), 7.60–7.66 (m, 1 H, H9), 7.66–7.71 (m, 2 H, H10, H14), 7.71–7.78 (m, 2 H, H6, H13), 8.17 (d, ^3^ *J* (H,H)=8 Hz, 1 H, H15), 8.24 (d, ^3^ *J* (H,H)=8 Hz, 1 H, H12) ppm; ^13^C NMR (125.75 MHz, [D_6_]DMSO, 25 °C): *δ*=13.1 (C1), 17.5 (Ca), 22.3 (Cg), 22.6 (Cg), 30.5 (Cf), 79.0 (Cc), 79.4 (Cd), 81.4 (Cb), 95.3 (Ce), 120.7 (C5), 121.1 (C15), 124.8 (C8), 125.8 (C9, C13), 127.5 (C6), 127.7 (C11, C16), 128.8 (C12, C14), 130.5 (C5), 130.7 (C10), 133.7 (C16), 137.0 (C2, C7), 167.8 (C4), 168.5 (C3) ppm.

ESI‐HR‐MS^+^ *m*/*z* found (calculated): [*M*]^+^ 662.0711 (662.0712). Elemental analysis calcd (%) for C_26_H_26_ClNOOsS⋅0.5 CH_2_Cl_2_: C 47.60, H 4.07, N 2.09, S 4.80; found: C 47.65, H 4.05, N 2.18, S 4.76.

**Chlorido[1,2‐dimethyl‐3‐oxo‐κ*O*‐pyridine‐4(1*H*)‐thionato‐κ*S*](η^5^‐1,2,3,4,5‐pentamethylcyclopentadienyl)rhodium(III) (5 a)**: The synthesis was performed according to the general complexation protocol using ligand **2 a** (50 mg, 0.320 mmol), sodium methoxide (21 mg, 0.384 mmol) and bis[dichlorido(η^5^‐1,2,3,4,5‐pentamethyl‐cyclopentadienyl)rhodium (III)] (89 mg, 0.144 mmol) and a reaction time of 2.0 h. The product was crystallized from DCM/*n*‐hexane and isolated as red crystals. Yield: 86 mg (70 %).

Monomer **5 a**: ^1^H NMR (500.10 MHz, [D_6_]DMSO, 25 °C): *δ*=1.64 (s, 15 H, CH_3_, Cp*), 2.45 (s, 3 H, H1), 3.86 (s, 3 H, H7), 7.22 (d, ^3^ *J* (H,H)=6 Hz, 1 H, H5), 7.54 (d, ^3^ *J* (H,H)=6 Hz, 1 H, H6) ppm; ^13^C‐NMR (125.75 MHz, [D_6_]DMSO, 25 °C): *δ*=8.4 (CH_3,_ Cp*), 12.3 (C1), 43.5 (C7), 98.9 (Cq, Cp*), 121.5 (C5), 129.4 (C6), 142.0 (C2), 148.0 (C4), 171.0 (C3) ppm.

Dimer 5 a*: ^1^H NMR (500.10 MHz, D_2_O, 25 °C): *δ*=1.19 (s, 15 H, CH_3_, Cp*), 1.60 (s, 15 H, Cp*), 2.14 (s, 3 H, H1), 2.71 (s, 3 H, H1), 3.94 (s, 3 H, H7), 4.08 (s, 3 H, H7), 7.31 (d, ^3^ *J* (H,H)=6 Hz, 1 H, H5), 7.41 (d, ^3^ *J* (H,H)=6 Hz, 1 H, H6), 7.70 (s, 2 H, H5, H6) ppm; ^13^C‐NMR (125.75 MHz, D_2_O, 25 °C): *δ*=7.1 (CH_3_, Cp*), 7.9 (CH_3_, Cp*), 11.9 (C1), 12.9(C1), 44.5 (C7), 45.2 (C7), 96.5 (Cq, Cp*), 97.0 (Cq, Cp*), 125.2 (C5), 126.0 (C5), 128.7 (C6), 130.2 (C6), 141.0 (C2), 143.5 (C2), 149.6 (2 C4), 169.5 (C3), 170.7 (C3) ppm. 1:1 ratio

ESI‐HR‐MS^+^ *m*/*z* found (calculated): [M]^+^ 392.0552 (392.0550). Elemental analysis calcd (%) for C_17_H_23_ClNORhS⋅H_2_O: C 45.80, H 5.65, N 3.14, S 7.19; found: C 45.73, H 5.38, N 3.15, S 7.46.

**Chlorido[1‐phenyl‐2‐methyl‐3‐oxo‐κ*O*‐pyridine‐4(1*H*)‐thionato‐κ*S*](η^5^‐1,2,3,4,5‐pentamethylcyclopentadienyl)rhodium(III) (5 b)**: The synthesis was performed according to the general complexation protocol using ligand **2 b** (75 mg, 0.345 mmol), sodium methoxide (22 mg, 0.414 mmol) and bis[dichlorido(η^5^‐1,2,3,4,5‐pentamethyl‐cyclopentadienyl)rhodium (III)] (96 mg, 0.155 mmol) and a reaction time of 2.0 h. The product was crystallized from DCM/*n*‐hexane and isolated as red crystals. Yield: 63 mg (42 %).

^1^H NMR (500.10 MHz, [D_6_]DMSO, 25 °C): *δ*=1.68 (s, 15 H, CH_3,_Cp*), 2.08 (s, 3 H, H1), 7.30 (d, ^3^ *J* (H,H)=6 Hz, 1 H, H5), 7.45–7.55 (m, 3 H, H9, H10, H11), 7.57–7.67 (m, 3 H, H6, H8, H12), 7.58–7.67 (m, 1 H, H6) ppm; ^13^C NMR (125.75 MHz, [D_6_]DMSO, 25 °C): *δ*=8.5 (CH_3,Cp*_), 14.2 (C1), 97.8 (Cq, Cp*), 121.3 (C5), 126.0 (C8, C12), 128.7 (C6), 129.9 (C9, C11), 130.0 (C10), 135.8 (C2), 141.4 (C7), 161.3 (C4), 166.2 (C3) ppm.

ESI‐HR‐MS^+^ *m*/*z* found (calculated): [*M*]^+^ 454.0708 (454.0706). Elemental analysis calcd (%) for C_22_H_25_ClNORhS⋅1.1 H_2_O: C 51.84, H 5.38, N 2.75, S 6.29; found: C 51.71, H 5.19, N 2.86, S 6.23.

**Chlorido[1‐benzyl‐2‐methyl‐3‐oxo‐κ*O*‐pyridine‐4(1*H*)‐thionato‐κ*S*](η^5^‐1,2,3,4,5‐pentamethylcyclopentadienyl)rhodium(III) (5 c)**: The synthesis was performed according to the general complexation protocol using ligand **2 c** (50 mg, 0.216 mmol), sodium methoxide (14 mg, 0.259 mmol) and bis[dichlorido(η^5^‐1,2,3,4,5‐pentamethyl‐cyclopentadienyl)rhodium (III)] (60 mg, 0.0973 mmol) and a reaction time of 2.0 h. The product was crystallized from DCM/*n*‐hexane and isolated as red crystals. Yield: 72 mg (74 %).

^1^H NMR (500.10 MHz, [D_6_]DMSO, 25 °C): *δ*=1.65 (s, 15 H, CH_3,_ Cp*), 2.36 (s, 3 H, H1), 5.51 (s, 2 H, H7), 7.10 (d, ^3^ *J* (H,H)=7 Hz, 2 H, H9, H13), 7.34 (d, ^3^ *J* (H,H)=6 Hz, 2 H, H10, H12), 7.40 (dd, ^3^ *J* (H,H)=7 Hz, ^3^ *J* (H,H)=7 Hz, 2 H, H5, H11), 7.58–7.70 (m, ^3^ *J* (H,H)=6 Hz, 1 H, H6) ppm; ^13^C NMR (125.75 MHz, [D_6_]DMSO, 25 °C): *δ*=8.4 (CH_3,_ Cp*), 12.3 (C1), 58.3 (C7), 98.7 (Cq, Cp*), 121.8 (C5), 126.6 (C9, C13), 128.2 (C10, C12), 129.1 (C11). 129.5 (C6), 135.1 (C8), 136.9 (C2), 159.7 (C4), 166.6 (C3) ppm.

ESI‐HR‐MS^+^ *m*/*z* found (calculated): [*M*]^+^ 468.0854 (468.0863). Elemental analysis calcd (%) for C_23_H_27_ClNORhS⋅0.75 H_2_O: C 53.39, H 5.55, N 2.71, S 6.20; found: C 53.21, H 5.47, N 2.62, S 5.99.

**Chlorido[1‐(naphthalene‐1‐yl)‐2‐methyl‐3‐oxo‐κ*O*‐pyridine‐4(1*H*)‐thionato‐κ*S*](η^5^‐1,2,3,4,5‐pentamethylcyclopentadienyl)rhodium(III) (5 d)**: The synthesis was performed according to the general complexation protocol using ligand **2 d** (85 mg, 0.320 mmol), sodium methoxide (26 mg, 0.480 mmol) and bis[dichlorido(η^5^‐1,2,3,4,5‐pentamethyl‐cyclopentadienyl)rhodium (III)] (100 mg, 0.160 mmol) and a reaction time of 2.0 h. The product was precipitated from DCM/*n*‐hexane and isolated as a red powder. Yield: 136 mg (79 %).

^1^H NMR (500.10 MHz, [D_6_]DMSO, 25 °C): *δ*=1.71 (s, 15 H, CH_3_,Cp*), 1.99 (s, 3 H, H1), 7.04 (d, ^3^ *J*(H,H)=8 Hz, 1 H, H5), 7.42 (d, ^3^ *J* (H,H)=7 Hz, 1 H, H8), 7.62–7.77 (m, 5 H, H6, H9, H10, H13, H14), 8.16 (d, ^3^ *J* (H,H)=8 Hz, 1 H, H15), 8.23 (d, ^3^ *J*(H,H)=8 Hz, 1 H, H12) ppm.

^13^C NMR (125.75 MHz, [D_6_]DMSO, 25 °C): *δ*=8.5 (CH_3_, Cp*), 13.4 (C1), 98.6 (Cq, Cp*), 120.7 (C5), 121.8 (C15), 124.6 (C8), 125.7 (C9, C13), 127.4 (C6), 127.7 (C11, C16), 128.7 (C12, C14), 129.6 (C5), 130.6 (C10), 133.7 (C16), 136.8 (C7), 137.2 (C2), 162.0 (C4), 166.2 (C3) ppm.

ESI‐HR‐MS^+^ *m*/*z* found (calculated): [*M*]^+^ 504.0856 (504.0863). Elemental analysis calcd (%) for C_26_H_27_ClNORhS⋅0.25 H_2_O: C 57.36, H 5.09, N 2.57, S 5.89; found: C 57.24, H 5.24, N 2.50, S 5.56.

**Chlorido[1,2‐dimethyl‐3‐oxo‐κ*O*‐pyridine‐4(1*H*)‐thionato‐κ*S*](*η*^5^‐1,2,3,4,5‐pentamethylcyclopentadienyl)iridium(III) (6 a)**: The synthesis was performed according to the general complexation protocol using ligand **2 a** (50 mg, 0.322 mmol), sodium methoxide (26 mg, 0.483 mmol) and bis[dichlorido(η^5^‐1,2,3,4,5‐pentamethyl‐cyclopentadienyl)iridium(III)] (128 mg, 0.161 mmol) and a reaction time of 2.0 h. The product was precipitated from DCM/*n*‐hexane and isolated as an orange solid. Yield: 124 mg (74 %).

^1^H NMR (500.10 MHz, [D_6_]DMSO, 25 °C): *δ*=1.71 (s, 15 H, CH_3_, Cp*), 3.90 (s, 3 H, H1), 7.33 (d, ^3^ *J* (H,H)=6 Hz, 1 H, H5), 7.63 (d, ^3^ *J* (H,H)=6 Hz, 1 H, H6) ppm;

^13^C NMR (125.75 MHz, [D_6_]DMSO, 25 °C): *δ*=8.1 (CH_3_, Cp*), 12.2 (C1), 43.4 (C7), 93.7 (Cq, Cp*), 121.3 (C5), 130.4 (C6), 138.3 (C2), 156.8 (C4), 167.5 (C3) ppm, H7 signal under DMSO peak.

ESI‐HR‐MS^+^ *m*/*z* found (calculated): [*M*]^+^ 482.1104 (482.1123). Elemental analysis calcd (%) for C_17_H_23_ClIrNOS⋅1.2 H_2_O: C 37.90, H 4.75, N 2.60, S 5.95; found: C 37.98, H 4.82, N 2.58, S 5.87.

**Chlorido[1‐phenyl‐2‐methyl‐3‐oxo‐κ*O*‐pyridine‐4(1*H*)‐thionato‐κ*S*](η^5^‐1,2,3,4,5‐pentamethylcyclopentadienyl)iridium(III) (6 b)**: The synthesis was performed according to the general complexation protocol using ligand **2 b** (54 mg, 0.250 mmol), sodium methoxide (20 mg, 0.380 mmol) and bis[dichlorido(η^5^‐1,2,3,4,5‐pentamethyl‐cyclopentadienyl)iridium(III)] (100 mg, 0.130 mmol) and a reaction time of 2.0 h. The product was precipitated from DCM/*n*‐hexane and isolated as a red powder. Yield: 120 mg (83 %).

^1^H NMR (500.10 MHz, [D_6_]DMSO, 25 °C): *δ*=1.74 (s, 15 H, CH_3_, Cp*), 2.19 (s, 3 H, H1), 7.45 (d, ^3^ *J* (H,H)=6 Hz, 1 H, H5), 7.54–7.59 (m, 2 H, H8, H12), 7.60–7.66 (m, 4 H, H6, H9, H10, H11) ppm;

^13^C NMR (125.75 MHz, [D_6_]DMSO, 25 °C): *δ*=8.1 (CH_3_, Cp*), 14.2 (C1), 93.8 (Cq, Cp*), 121.4 (C5), 126.0 (C8, C12), 129.9 (C6), 130.1 (C9, C11), 130.2 (C10), 137.2 (C2), 141.1 (C7), 159.4 (C4), 167.5 (C3) ppm.

ESI‐HR‐MS^+^ *m*/*z* found (calculated): [*M*]^+^ 544.1265 (544.1280). Elemental analysis calcd (%) for C_22_H_25_ClIrNOS⋅0.75 H_2_O: C 44.58, H 4.51, N 2.36, S 5.41; found: C 44.25, H 4.52, N 2.35, S 5.43.

**Chlorido[1‐benzyl‐2‐methyl‐3‐oxo‐κ*O*‐pyridine‐4(1*H*)‐thionato‐κ*S*](η^5^‐1,2,3,4,5‐pentamethylcyclopentadienyl)iridium(III) (6 c)**: The synthesis was performed according to the general complexation protocol using ligand **2 c** (58 mg, 0.250 mmol), sodium methoxide (20 mg, 0.380 mmol) and bis[dichlorido(η^5^‐1,2,3,4,5‐pentamethyl‐cyclopentadienyl)iridium(III)] (100 mg, 0.130 mmol) and a reaction time of 2.0 h. The product was precipitated from DCM/*n*‐hexane and isolated as an orange powder. Yield: 115 mg (78 %).

^1^H NMR (500.10 MHz, [D_6_]DMSO, 25 °C): *δ*=1.71 (s, 15 H, CH_3_, Cp*), 2.42 (s, 3 H, H1), 5.56 (s, 2 H, H7), 7.13 (d, ^3^ *J* (H,H)=6 Hz, 2 H, H9, H13), 7.40 (dd, ^3^ *J* (H,H)=7 Hz, ^3^ *J* (H,H)=7 Hz, 1 H, H11), 7.46 (d, ^3^ *J* (H,H)=6 Hz, 2 H, H10, H12), 7.46 (d, ^3^ *J* (H,H)=6 Hz, 1 H, H5), 7.80 (d, ^3^ *J* (H,H)=6 Hz, 1 H, H6) ppm;

^13^C NMR (125.75 MHz, [D_6_]DMSO, 25 °C): *δ*=8.1 (CH_3_, Cp*), 12.3 (C1), 58.2 (C7), 93.8 (Cq, Cp*), 121.7 (C5), 126.7 (C9, C13), 128.3 (C11), 129.1 (C10, C12), 130.5 (C6), 134.8 (C8), 137.5 (C2), 158.3 (C4), 168.0 (C3) ppm.

ESI‐HR‐MS^+^ *m*/*z* found (calculated): [*M*]^+^ 558.1422 (558.1436). Elemental analysis calcd (%) for C_23_H_27_ClIrNOS: C 46.57, H 4.59, N 2.36, S 5.41; found: C 46.46, H 4.75, N 2.44, S 5.42.

**Chlorido[1‐(naphthalene‐1‐yl)‐2‐methyl‐3‐oxo‐κ*O*‐pyridine‐4(1*H*)‐thionato‐κ*S*](η^5^‐1,2,3,4,5‐pentamethylcyclopentadienyl)iridium(III) (6 d)**: The synthesis was performed according to the general complexation protocol using ligand **2 d** (67 mg, 0.250 mmol), sodium methoxide (20 mg, 0.34 mmol) and bis[dichlorido(η^5^‐1,2,3,4,5‐pentamethyl‐cyclopentadienyl)iridium(III)] (100 mg, 0.13 mmol) and a reaction time of 2.0 h. The product was precipitated from DCM/*n*‐hexane and isolated as an orange powder. Yield: 114 mg (72 %).

^1^H NMR (500.10 MHz, [D_6_]DMSO, 25 °C): *δ*=1.71 (s, 15 H, CH_3_, Cp*), 1.99 (s, 3 H, H1), 7.07 (d, ^3^ *J*(H,H)=8 Hz, 1 H, H5), 7.55 (d, ^3^ *J* (H,H)=7 Hz, 1 H, H8), 7.61–7.82 (m, 5 H, H6, H9, H10, H13, H14), 8.17 (d, ^3^ *J* (H,H)=8 Hz, 1 H, H15), 8.25 (d, ^3^ *J*(H,H)=8 Hz, 1 H, H12) ppm.

^13^C NMR (125.75 MHz, [D_6_]DMSO, 25 °C): *δ*=8.1 (CH_3_, Cp*), 13.4 (C1), 93.9 (Cq, Cp*), 120.8 (C5), 121.8 (C15), 124.8 (C8), 125.7 (C9, C13), 127.5 (C6), 127.6 (C11, C16), 128.7 (C12, C14), 130.8 (C5, C10), 133.7 (C16), 136.9 (C7), 137.7 (C2), 160.5 (C4), 167.6 (C3) ppm.

ESI‐HR‐MS^+^ *m*/*z* found (calculated): [*M*]^+^ 594.1424 (594.1436). Elemental analysis calcd (%) for C_26_H_27_ClIrNOS: C 49.63, H 4.33, N 2.23, S 5.10; found: C 49.93, H 4.64, N 2.28, S 4.97.

## Conflict of interest

The authors declare no conflict of interest.

## Supporting information

As a service to our authors and readers, this journal provides supporting information supplied by the authors. Such materials are peer reviewed and may be re‐organized for online delivery, but are not copy‐edited or typeset. Technical support issues arising from supporting information (other than missing files) should be addressed to the authors.

Supplementary
