# Supplementary material for: Synthesis, Modification, and Biological Evaluation of a Library of Novel Water‐Soluble Thiopyridone‐Based Organometallic Complexes and Their Unexpected (Biological) Behavior
Source: Chemistry. 2020 Apr 6;26(24):5419–33. doi: 10.1002/chem.201905546 (PMC7217150; doi:10.1002/chem.201905546)
Supplement: Supplementary file 1 — Supplementary [file CHEM-26-5419-s001.pdf]

Chemistry–A European Journal

Supporting Information

Synthesis, Modification, and Biological Evaluation of a Library of Novel Water-Soluble Thiopyridone-Based Organometallic Complexes and Their Unexpected (Biological) Behavior

Sophia Harringer,[a] Barbara Happl,[a, b, c] Marius Ozenil,[c] Caroline Kast,[a] Michaela Hejl,[a] Debora Wernitznig,[a] Anton A. Legin,[a] Andreas Schweikert,[a, d] Natalie Gajic,[a] Alexander Roller,[a] Gunda Koellensperger,[d] Michael A. Jakupec,[a, e] Wolfgang Kandioller,*[a, e] and Bernhard K. Keppler[a, e]

Contents

General procedures 2
Ligand NMR spectra 6
NMR Spectra in d_6-DMSO 9
NMR Spectra in D_2O (Charges omitted for clarity) 25
X-ray diffraction analysis 34
UV-Vis 35
HPLC stabilities 43
HPLC incubation 58
Chromatographic lipophilicity index φ_0 61
Cytotoxicity in cancer cell lines (monolayer cultures) 63
Cell cycle studies 67

General procedures

General protocol for syntheses of pyridone ligands (1b–d): Syntheses of all ligands were performed by combining maltol (1 eq) and the respective amine (2 eq) in 0.38 M HCl (10 mL) followed by reaction under microwave conditions (T = 165 °C, t = 30 min, P_{max} = 200 W, p_{max} = 10 bar). The resulting solid was separated *via* decantation and recrystallized from MeOH. The products were obtained in moderate to good yields (56–74%).

3-Hydroxy-2-methyl-1-phenylpyridin-4(1H)-one (**1b**): The synthesis was performed according to the general procedure for pyridone ligand syntheses using maltol (1.50 g, 11.9 mmol, and aniline (3.33 mL, 23.8 mmol). The product was isolated as a beige powder. Yield: 1.35 g (56%). ^{1}H-NMR (500.10 MHz, 298.2 K, d_6-DMSO): δ = 2.24 (s, 3H, H1), 6.33 (d, ^3J (H,H) = 7 Hz, 1H, H5), 7.13–7.28 (m, 2H, H8, H12), 7.33–7.43 (m, 3H, H9, H10, H11), 8.03 (d, ^3J (H,H) = 6 Hz, 1H, H6) ppm.

1-Benzyl-3-hydroxy-2-methylpyridin-4(1H)-one (**1c**): The synthesis was performed according to the general procedure for pyridone ligand syntheses using maltol (1.50 g, 11.9 mmol) and benzylamine (2.55 g, 23.8 mmol). The product was isolated as yellow crystals. Yield: 1.43 g (56%). ^{1}H-NMR (500.10 MHz, 298.2 K, d_6-DMSO): δ = 2.11 (s, 3H, H1), 5.25 (s, 2H, H7), 6.19 (d, ^3J (H,H) = 7 Hz, 1H, H5), 7.07 (d, ^3J (H,H) = 8 Hz, 2H, H9, H13), 7.27–7.33 (m, 1H, H11), 7.35–7.41 (m, 2H, H9, H10, H12), 7.75 (d, ^3J (H,H) = 7 Hz, 1H, H6) ppm.

3-Hydroxy-2-methyl-1-(naphthalene-1-yl)pyridin-4(1H)-one (**1d**): The synthesis was performed according to the general procedure for pyridone ligand syntheses using maltol (1.50 g, 11.9 mmol) and 1-naphthylamine (3.41 g, 23.8 mmol). The product was isolated as violet crystals. Yield: 2.24 g (74%). ^{1}H-NMR (500.10 MHz, 298.2 K, d_6-DMSO): δ = 1.79 (s, 3H, H1), 6.31 (d, ^3J (H,H) = 7 Hz, 1H, H5), 7.24 (d, ^3J (H,H) = 8 Hz, 1H, H15), 7.61–7.68 (m, 3H, H6, H13, H14), 7.69 (dd, ^{3}J (H,H) = 5 Hz, ^4J (H,H) = 1 Hz, 2H, H8, H10), 8.13 (dd, ^3J (H,H) = 8 Hz, ^4J (H,H) = 1 Hz, 1H, H12), 8.14–8.17 (m, 1H, H9) ppm.

General protocol for thiopyridone ligand syntheses (2b–2d): Thionations were performed *via* refluxation of the respective pyridone ligand (1 eq) and Lawesson's reagent (0.5 eq) in dry toluene (15 mL) for several hours. The reaction mixture was then concentrated *in vacuo* and the product was obtained after recrystallization from MeOH in moderate yields (49-70%).

3-Hydroxy-2-methyl-1-phenylpyridin-4(1*H*)-thione (**2b**): The synthesis was performed according to the general thionation protocol using compound **1b** (300 mg, 1.49 mmol,) and Lawesson's reagent (303 mg, 0.75 mmol,), which were refluxed for 4.5 h. The product was obtained as beige crystals. Yield: 207 mg (64%). ESI-MS$^+$ *m/z* found (calculated): $[M]^+$ 218.26 (218.06). Elemental analysis calcd (%) for $C_{12}H_{11}NOS$: C 66.33, H 5.10, N 6.45, S 14.76; found: C 66.39, H 5.07, N 6.46, S 14.48. ^{1}H-NMR (600.25 MHz, 298.2 K, d_6-DMSO): δ = 2.12 (s, 3H, H1), 7.38 (d, ^3J (H,H) = 7 Hz, 1H, H5), 7.56–7.59 (m, 2H, H8, H12), 7.60–7.66 (m, 4H, H6, H9, H10, H11), 8.78 (d, ^3J (H,H) = 6 Hz, 1H,

OH) ppm. ^{13}C-NMR (150.95 MHz, 298.2 K, d_6-DMSO): δ = 14.2 (C1), 124.5 (C5), 126.5 (C8, C12). 128.1 (C7), 129.9 (C9–C11), 133.0 (C6), 141.2 (C2), 152.0 (C4), 170.8 (C3).

3-Hydroxy-2-methyl-1-benzylpyridin-4(1*H*)-thione (**2c**): The synthesis was performed according to the general thionation protocol using compound **1c** (300 mg, 1.39 mmol,) and Lawesson's reagent (283 mg, 0.70 mmol,), which were refluxed for 16 h. The product was obtained as yellow crystals. Yield: 159 mg (49%). ESI-MS$^+$ *m/z* found (calculated): $[M]^+$ 232.25 (232.08), $[M+Na]^+$ 254.24 (254.06). Elemental analysis calcd (%) for $C_{13}H_{13}NOS\cdot 0.1\ H_2O$: C 66.98, H 5.71, N 6.01, S 13.76; found: C 66.87, H 5.67, N 6.07, S 14.10. ^{1}H-NMR (600.25 MHz, 298.2 K, d_6-DMSO): δ = 2.31 (s, 3H, H1), 5.47 (s, 2H, H7), 7.13 (d, ^3J (H,H) = 7 Hz, 2H, H5, H13), 7.32-7.36 (m, 1H, H9), 7.38–7.43 (m, 3H, H10–H12), 7.84 (d, ^3J (H,H) = 7 Hz, 1H, H6), 8.75 (s, 1H, OH) ppm. ^{13}C-NMR (150.95 MHz, 298.2 K, d_6-DMSO): δ = 12.5 (C1), 57.6 (C7), 124.9 (C11), 126.5 (C5, C9). 128.1 (C13), 128.3 (C8), 129.1 (C10, C12), 133.6 (C6), 135.5 (C2), 152.7 (C4), 169.6 (C3).

3-Hydroxy-2-methyl-1-(naphthalene-1-yl)pyridine-4(1*H*)-thione (**2d**): The synthesis was performed according to the general thionation protocol using compound **1d** (1.13 g, 4.49 mmol,) and Lawesson's reagent (0.908 g, 2.25 mmol,), which were refluxed for 8 h. The product was obtained as a grey powder. Yield: 724 mg (60%). ESI-MS$^+$ *m/z* found (calculated): $[M]^+$ 268.08 (268.08), $[M+Na]^+$ 290.03 (290.06). Elemental analysis calcd (%) for $C_{16}H_{13}NOS\cdot 0.05\ H_2O$: C 71.64, H 4.92, N 5.22, S 11.95; found: C 71.45, H 4.83,

N 5.27, S 11.81. ^{1}H-NMR (600.25 MHz, 298.2 K, d_6-DMSO): δ = 1.97 (s, 3H, H1), 7.23 (d, ^{3}J (H,H) = 8 Hz, 1H, H5), 7.48 (d, ^{3}J (H,H) = 7 Hz, 1H, H8), 7.62–7.65 (m, 1H, H14), 7.66–7.68 (m, 1H, H9), 7.75 (dd, ^{3}J (H,H) = 5 Hz, ^{4}J (H,H) = 1 Hz, 2H, H6, H10), 7.81 (dd, ^{3}J (H,H) = 5 Hz, ^{4}J (H,H) = 1 Hz, 1H, H13), 8.16 (d, ^{3}J (H,H) = 8 Hz, 1H, H15), 8.23 (d, ^{3}J (H,H) = 8 Hz, 1H, H12), 8.86 (s, 1H, OH) ppm. ^{13}C-NMR (150.95 MHz, 298.2 K, d_6-DMSO): δ = 13.4 (C1), 121.0 (C5), 124.8 (C8), 125.3 (C13), 125.7 (C10), 127.4 (C9). 128.3 (C11), 128.6 (C16, C15), 128.7 (C14), 130.5 (C12), 133.5 (C6), 133.7 (C7), 137.0 (C2), 152.2 (C4), 171.7 (C3).

Ligand NMR spectra

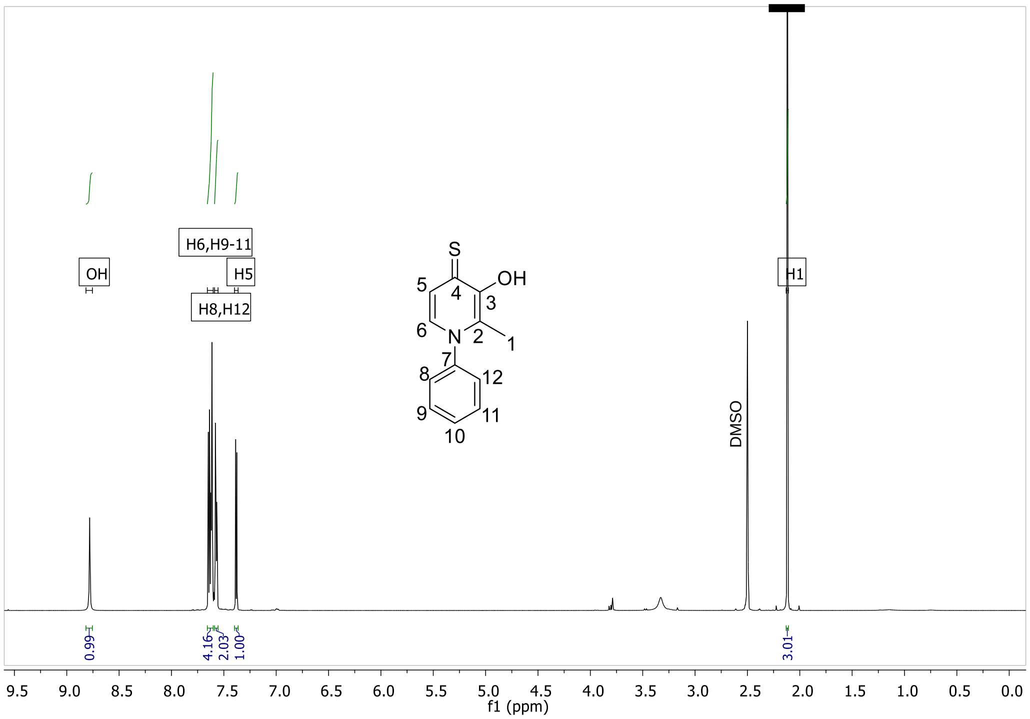

Figure S1. ^{1}H NMR of ligand **2b** (600.25 MHz, d_6-DMSO, 25 °C).

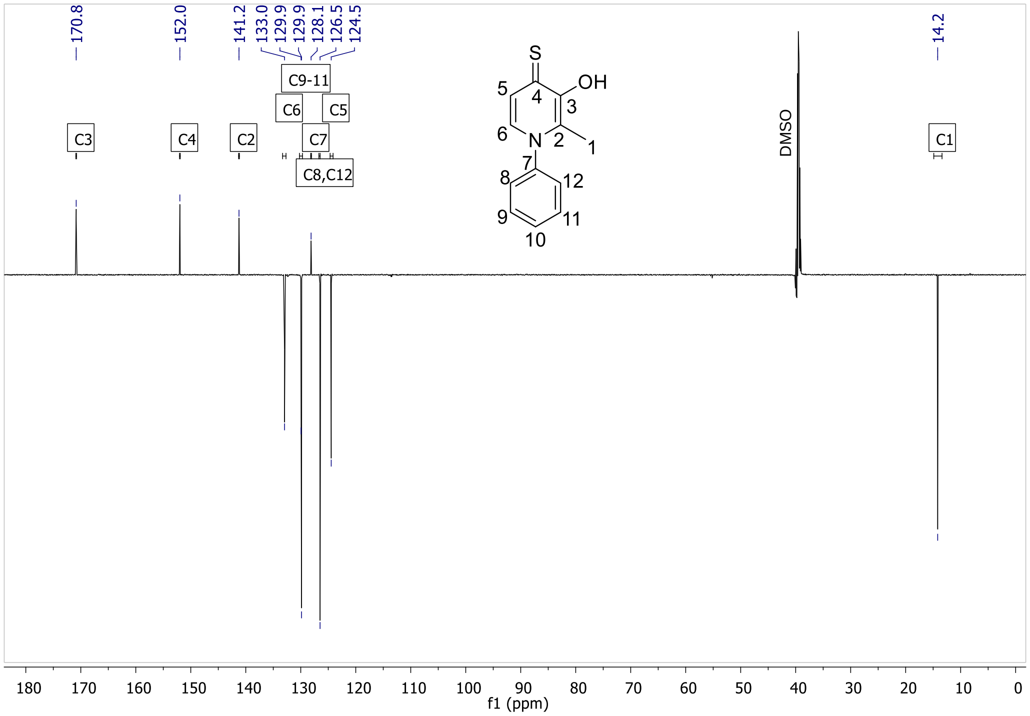

Figure S2. ^{13}C NMR of ligand **2b** (150.95 MHz, d_6-DMSO, 25 °C).

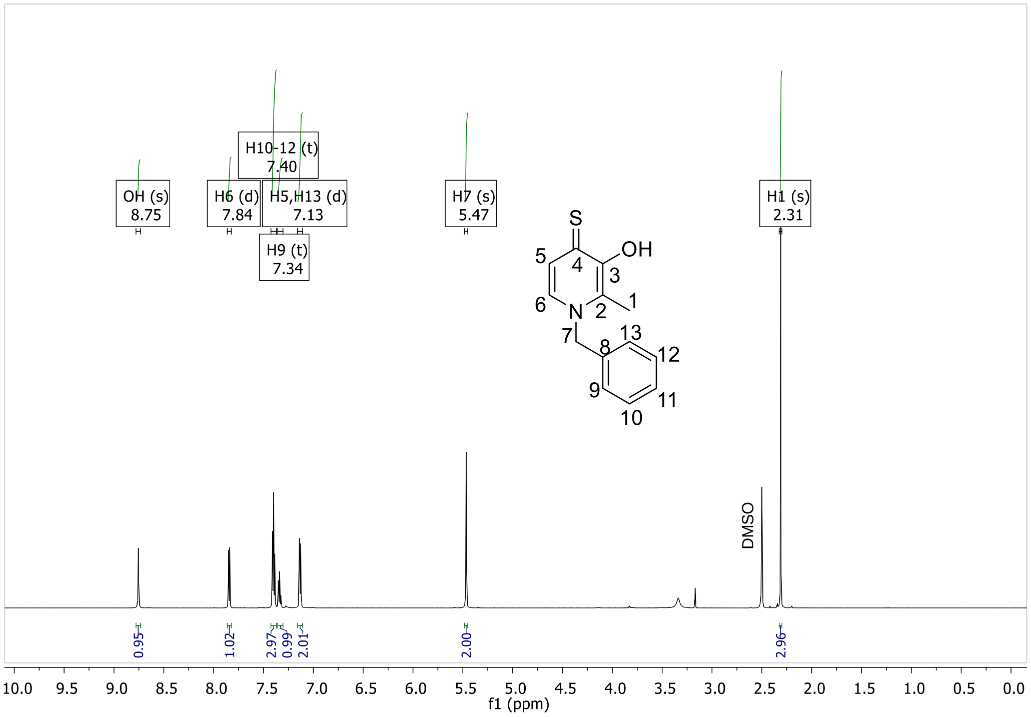

Figure S3. ^{1}H NMR of ligand **2c** (600.25 MHz, d_6-DMSO, 25 °C).

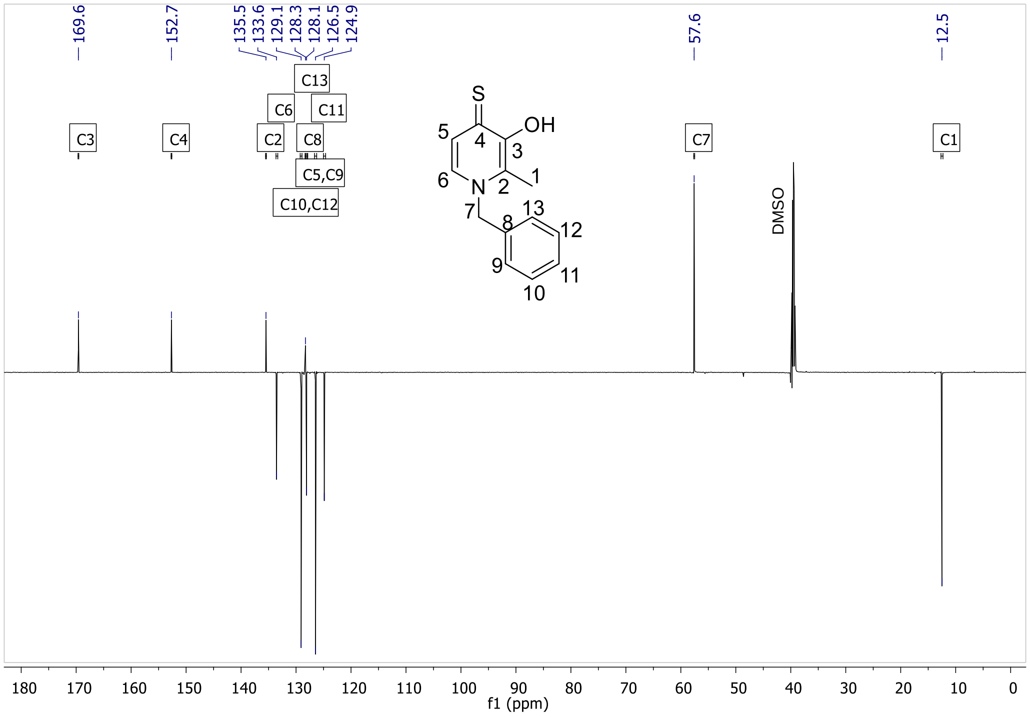

Figure S4. ^{13}C NMR of ligand **2c** (150.95 MHz, d_6-DMSO, 25 °C).

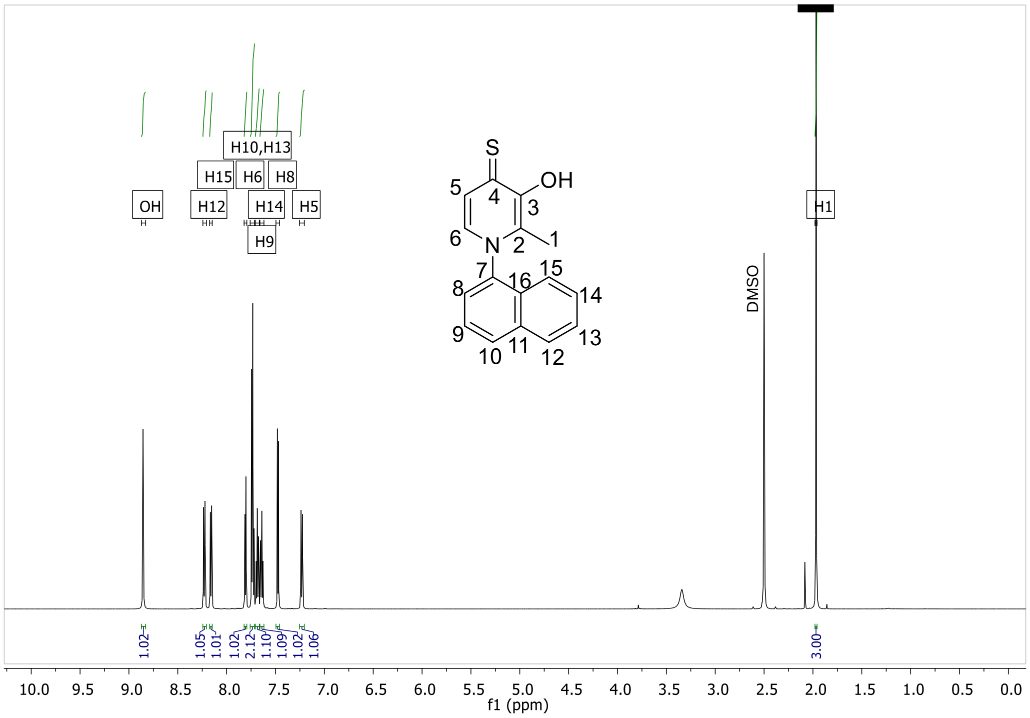

Figure S5. ^{1}H NMR of ligand **2d** (600.25 MHz, d_6-DMSO, 25 °C).

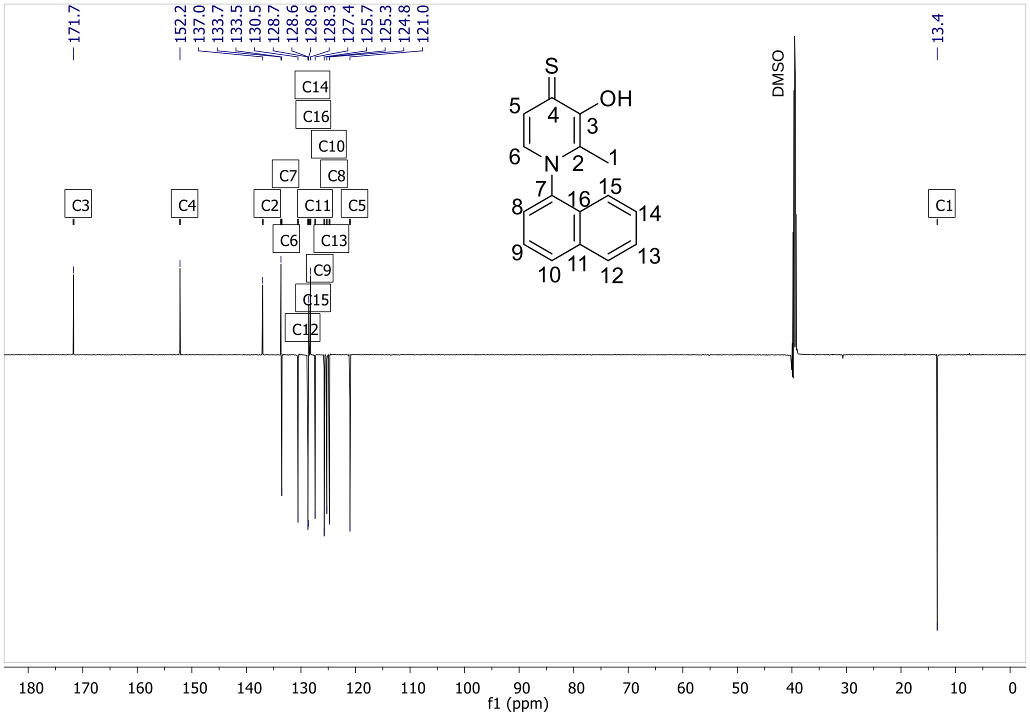

Figure S6. ^{13}C NMR of ligand **2d** (150.95 MHz, d_6-DMSO, 25 °C)

NMR Spectra in d_6-DMSO

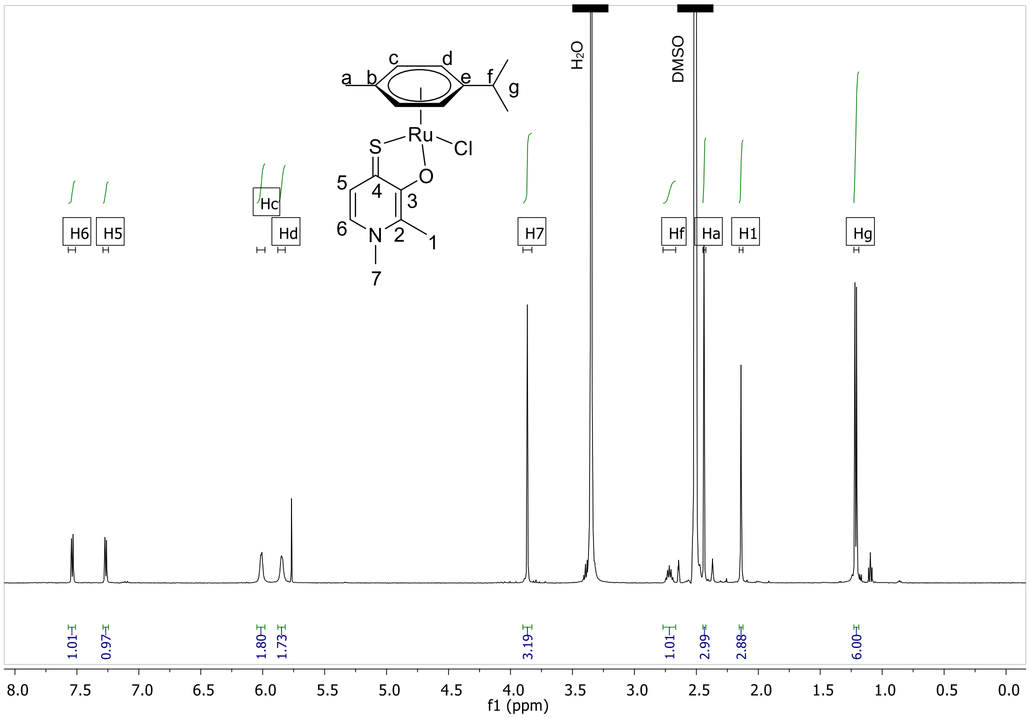

Figure S7. ^{1}H NMR of complex **3a** (500.10 MHz, d_6-DMSO, 25 °C).

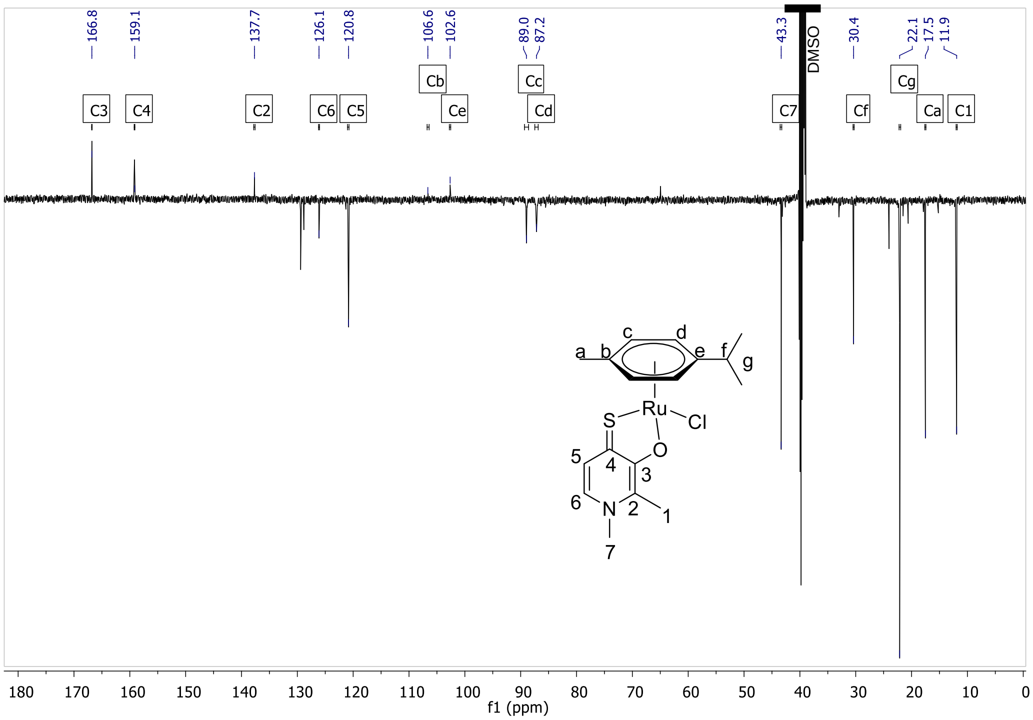

Figure S8. ^{13}C NMR of complex **3a** (125.75 MHz, d_6-DMSO, 25 °C).

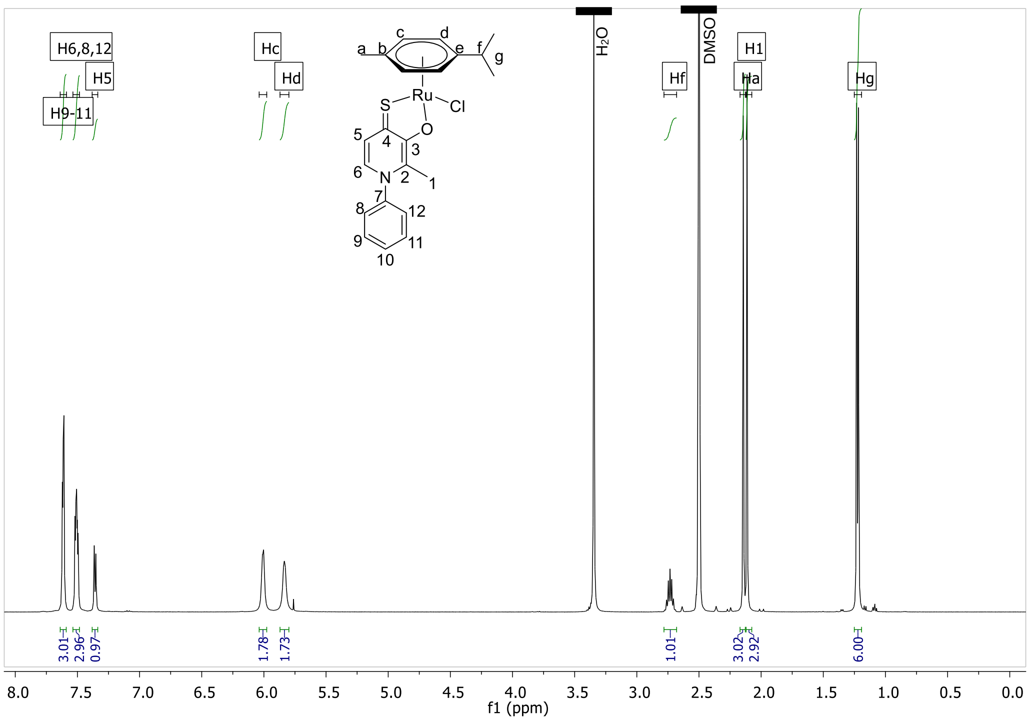

Figure S9. ^{1}H NMR of complex **3b** (500.10 MHz, d_6-DMSO, 25 °C).

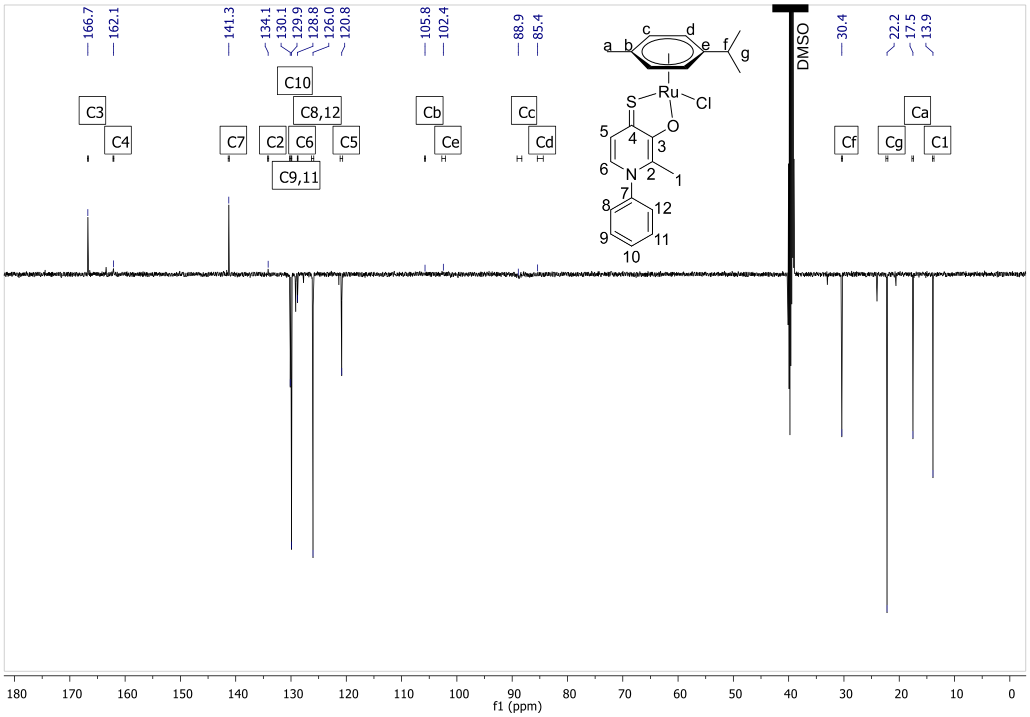

Figure S10. ^{13}C NMR of complex **3b** (125.75 MHz, d_6-DMSO, 25 °C).

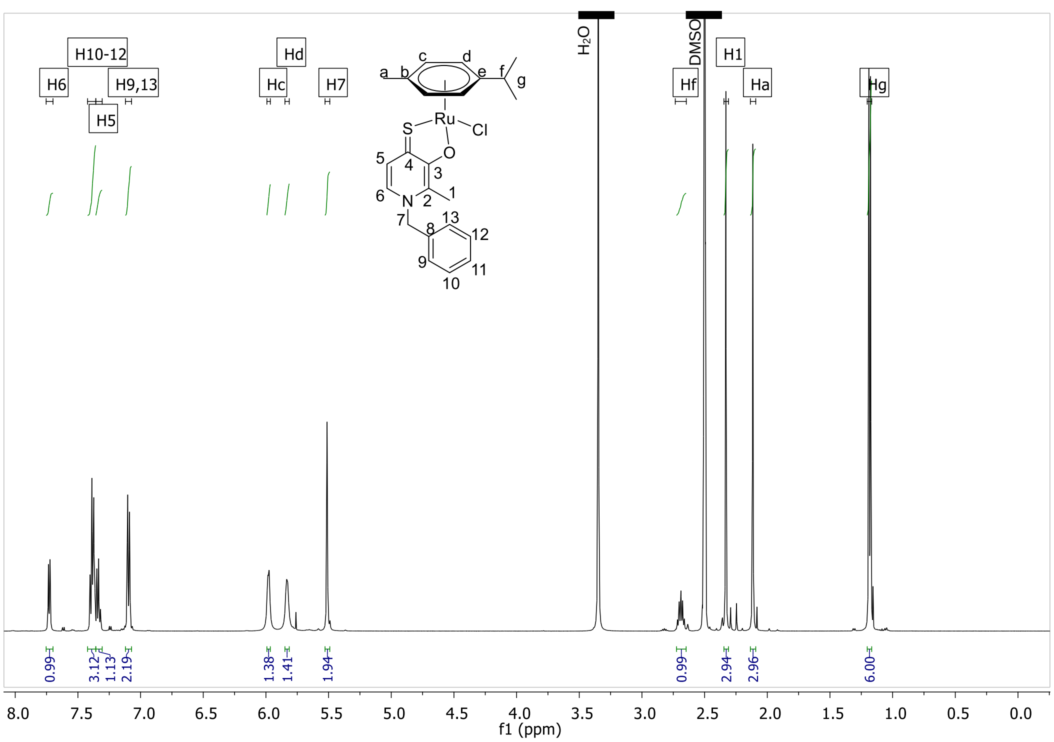

Figure S11. ^{1}H NMR of complex **3c** (500.10 MHz, d_6-DMSO, 25 °C).

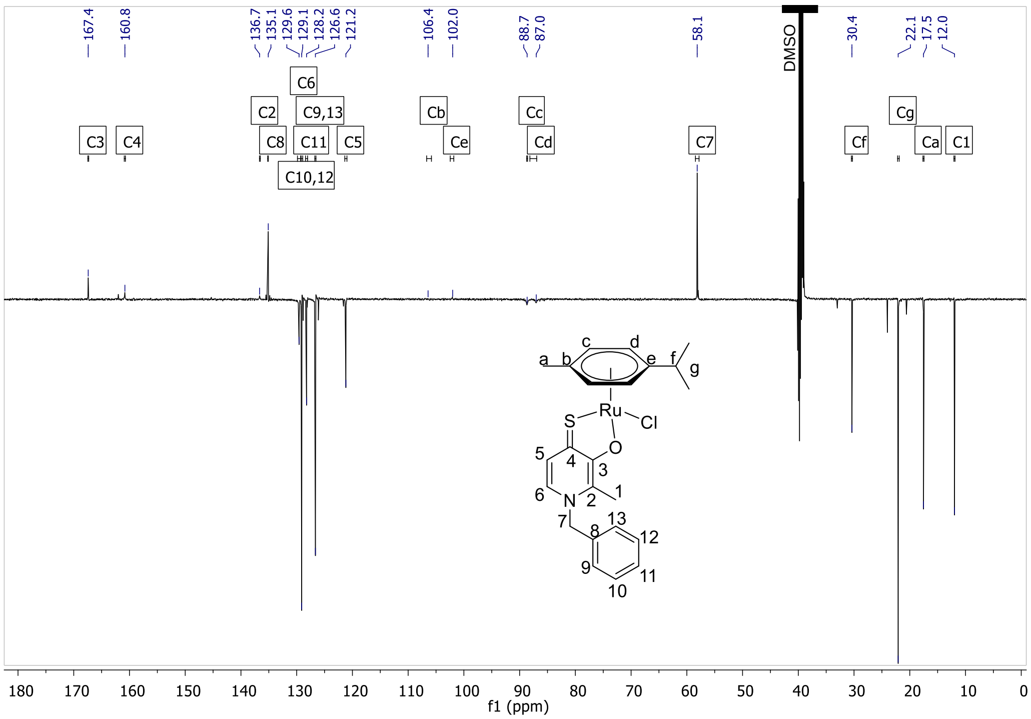

Figure S12. ^{13}C NMR of complex **3c** (125.75 MHz, d_6-DMSO, 25 °C).

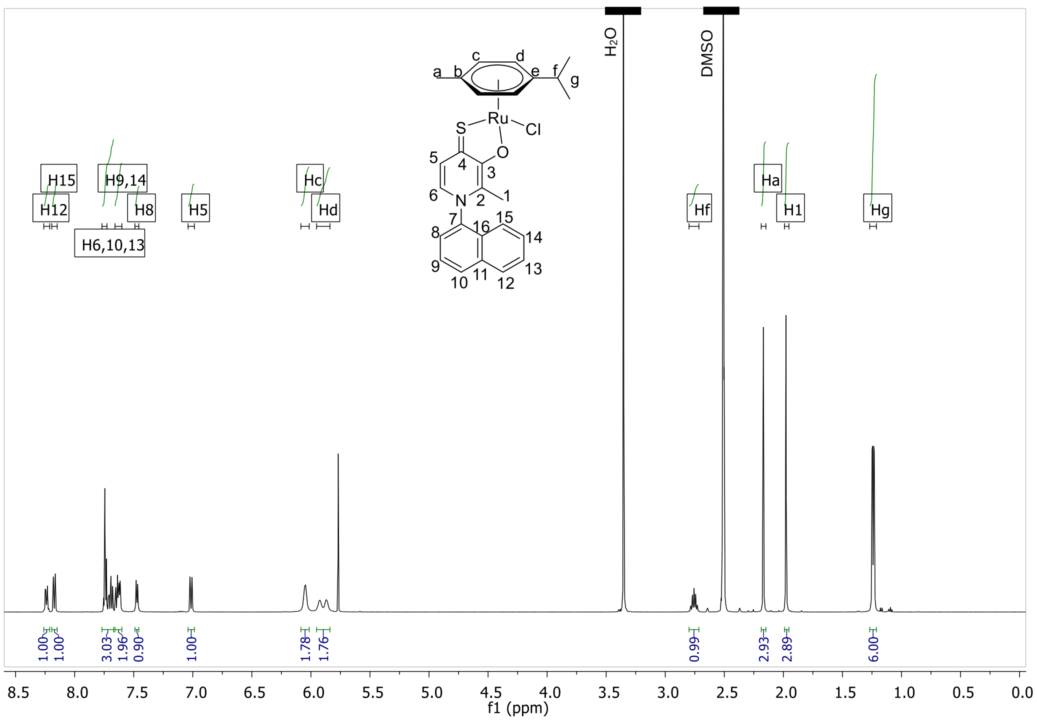

Figure S13. ^{1}H NMR of complex **3d** (500.10 MHz, d$_6$-DMSO, 25 °C).

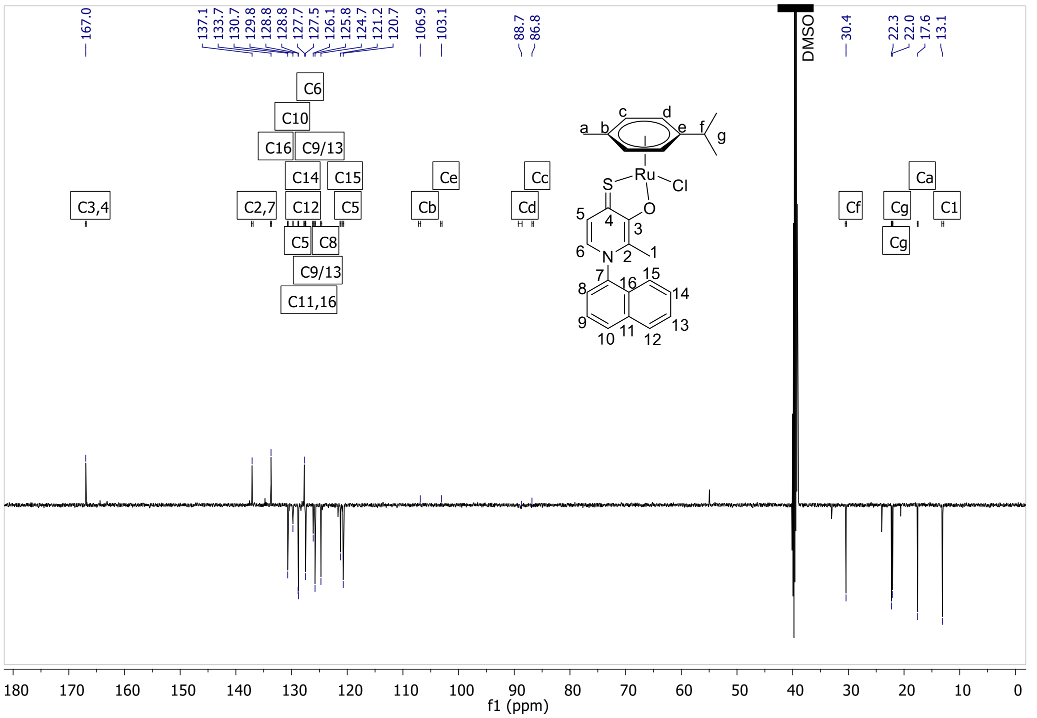

Figure S14. ^{13}C NMR of complex **3d** (125.75 MHz, d$_6$-DMSO, 25 °C).

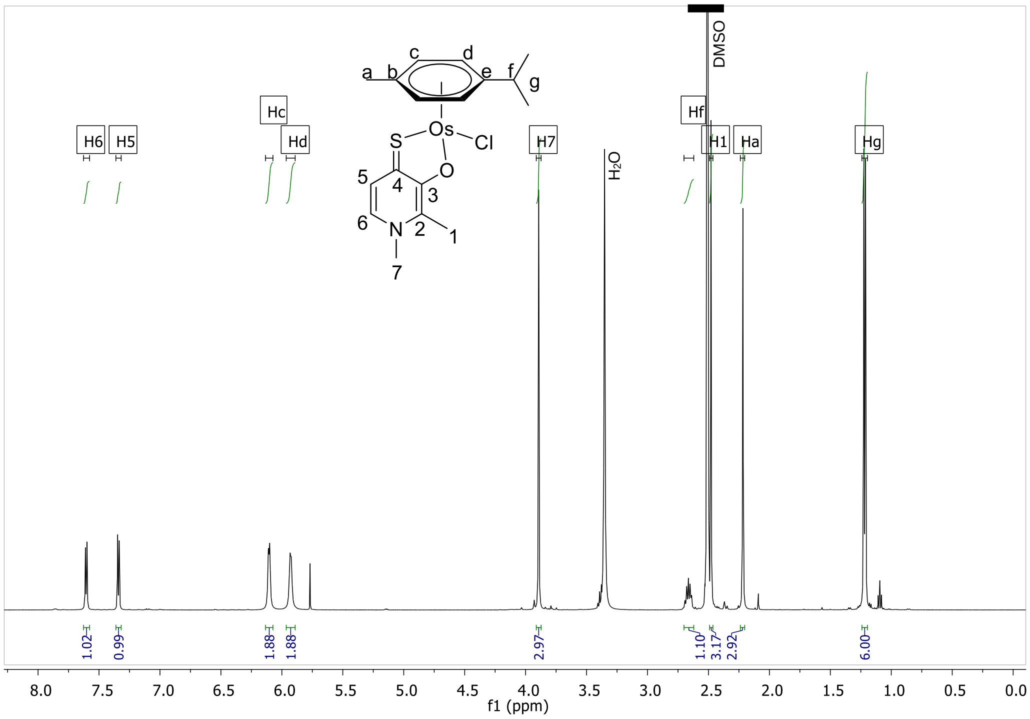

Figure S15. ^{1}H NMR of complex **4a** (500.10 MHz, d_6-DMSO, 25 °C).

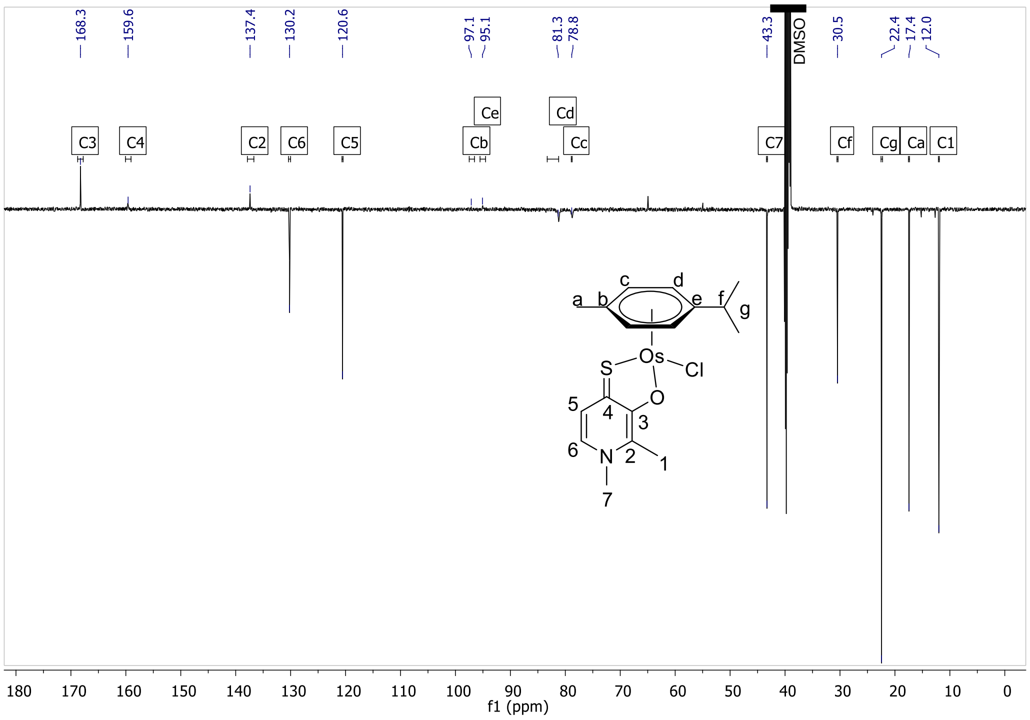

Figure S16. ^{13}C NMR of complex **4a** (125.75 MHz, d_6-DMSO, 25 °C).

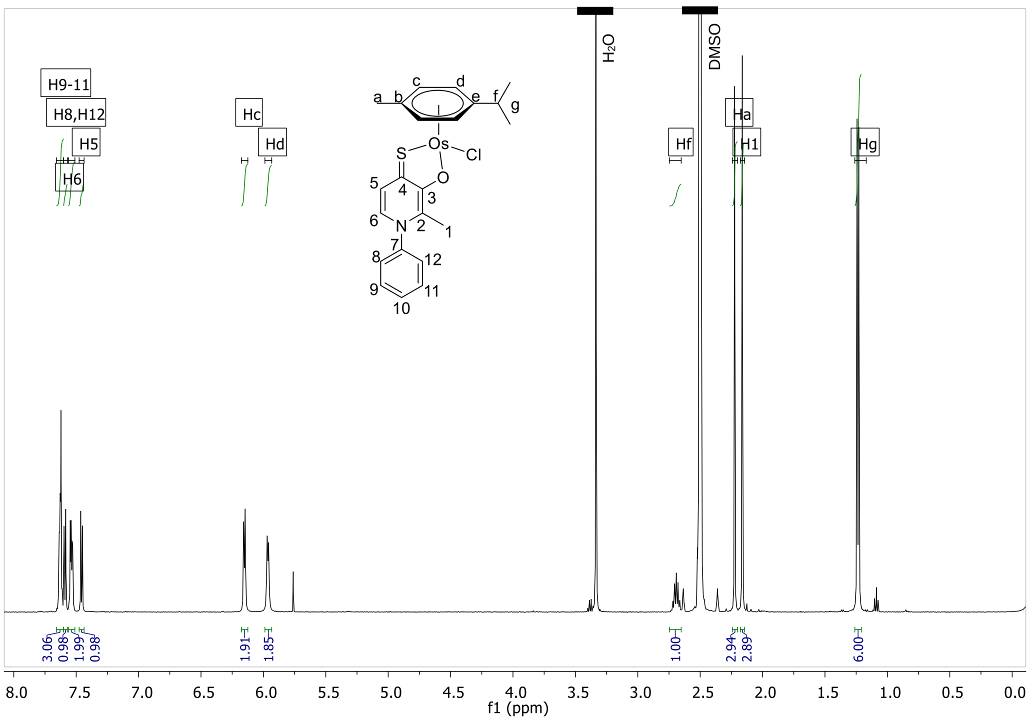

Figure S17. ^{1}H NMR of complex **4b** (500.10 MHz, d_6-DMSO, 25 °C).

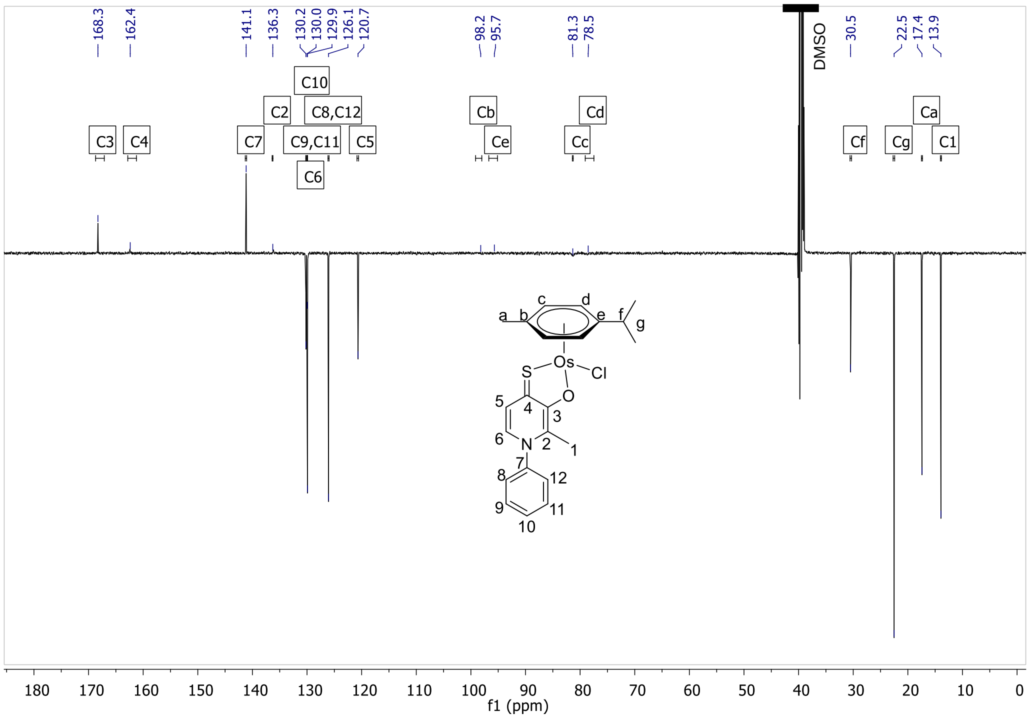

Figure S18. ^{13}C NMR of complex **4b** (125.75 MHz, d_6-DMSO, 25 °C).

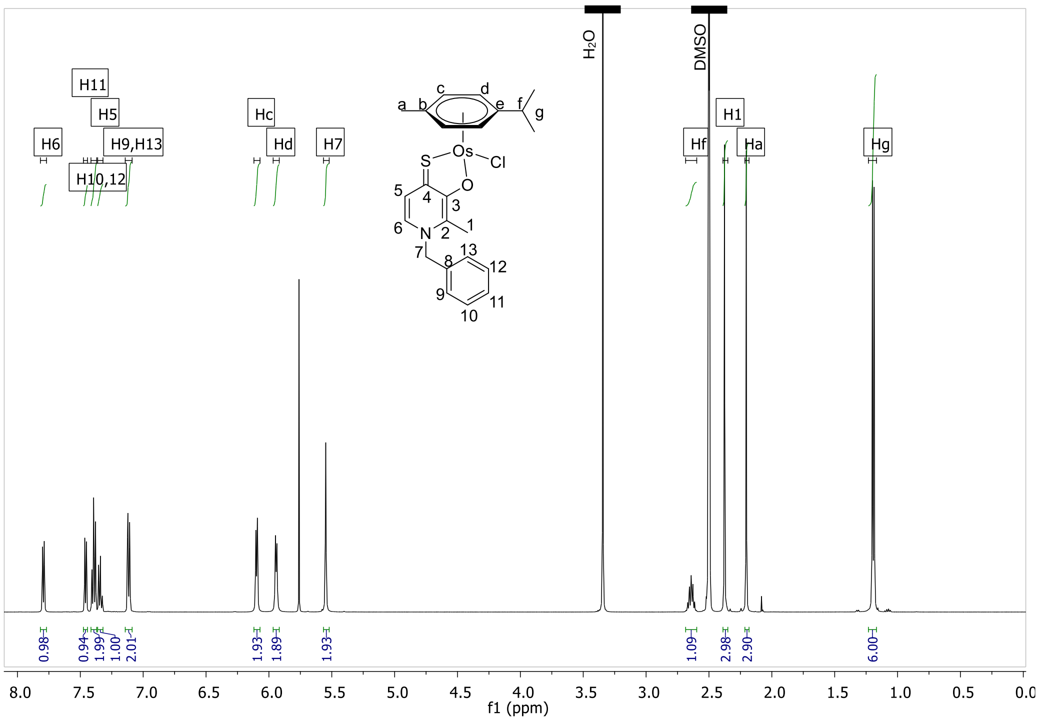

Figure S19. ^{1}H NMR of complex **4c** (500.10 MHz, d$_6$-DMSO, 25 °C).

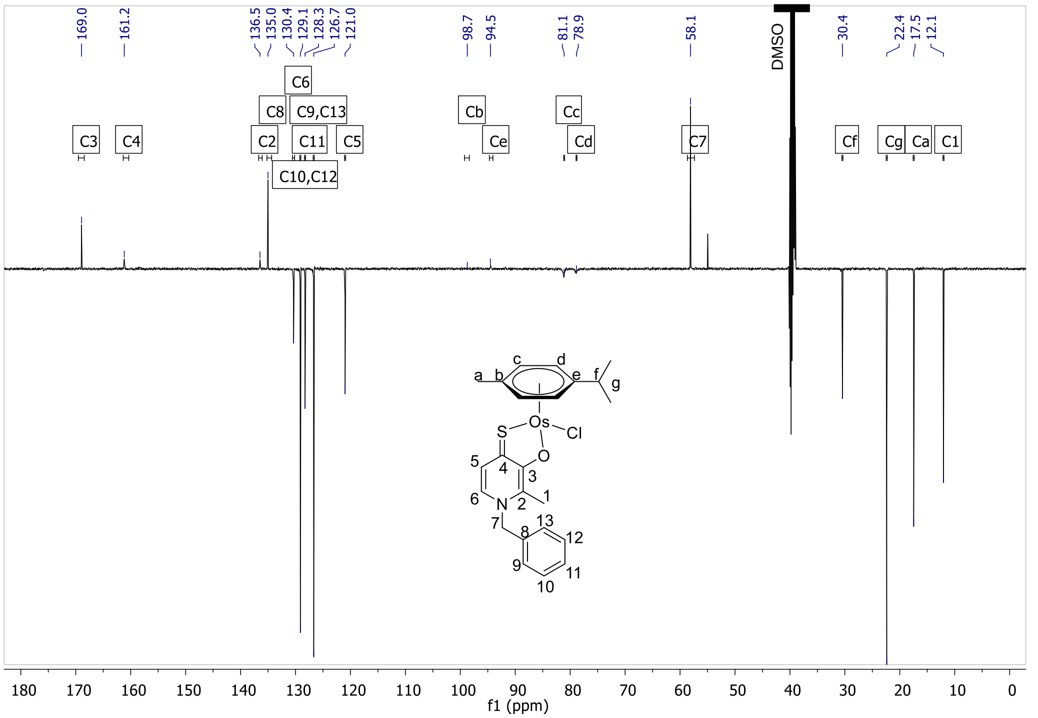

Figure S20. ^{13}C NMR of complex **4c** (125.75 MHz, d$_6$-DMSO, 25 °C).

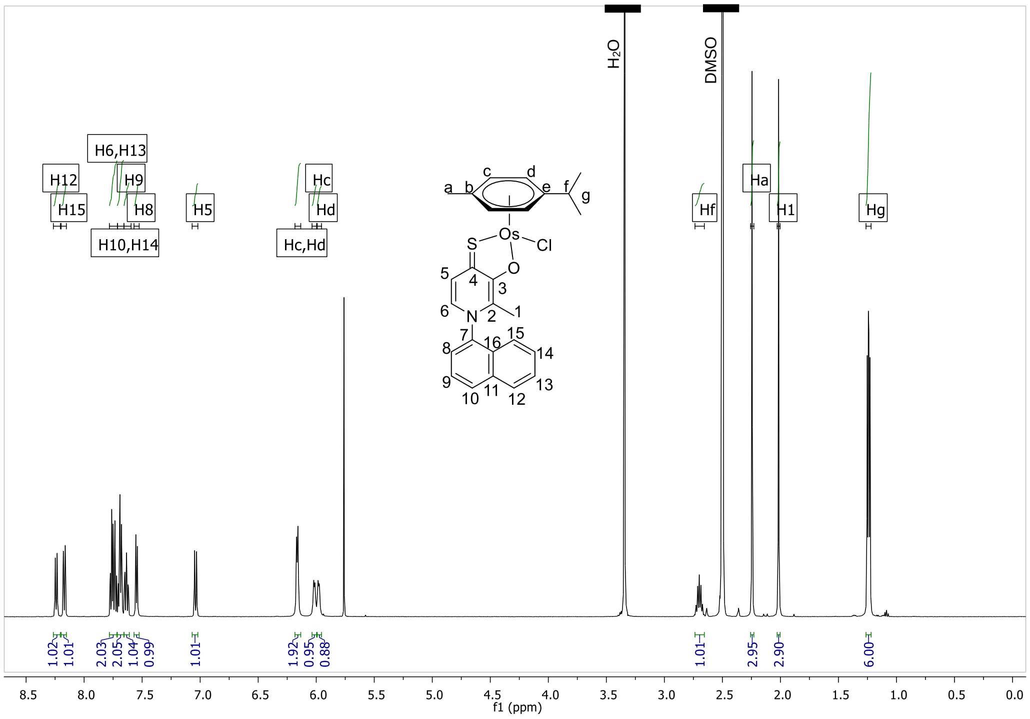

Figure S21. ^{1}H NMR of complex **4d** (500.10 MHz, d_6-DMSO, 25 °C).

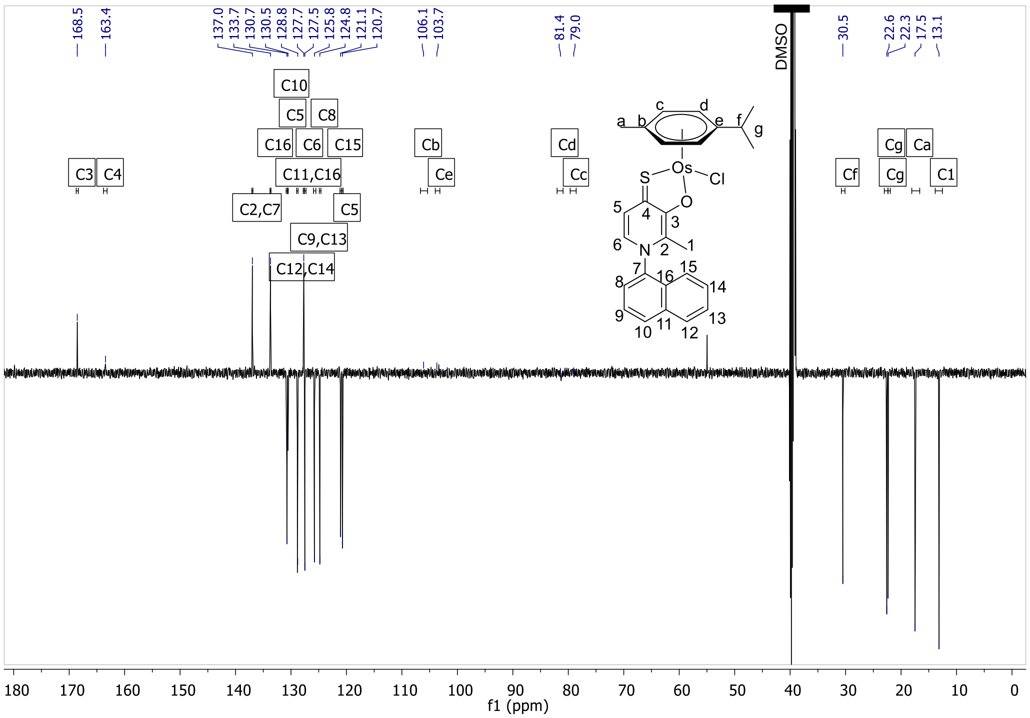

Figure S22. ^{13}C NMR of complex **4d** (125.75 MHz, d_6-DMSO, 25 °C).

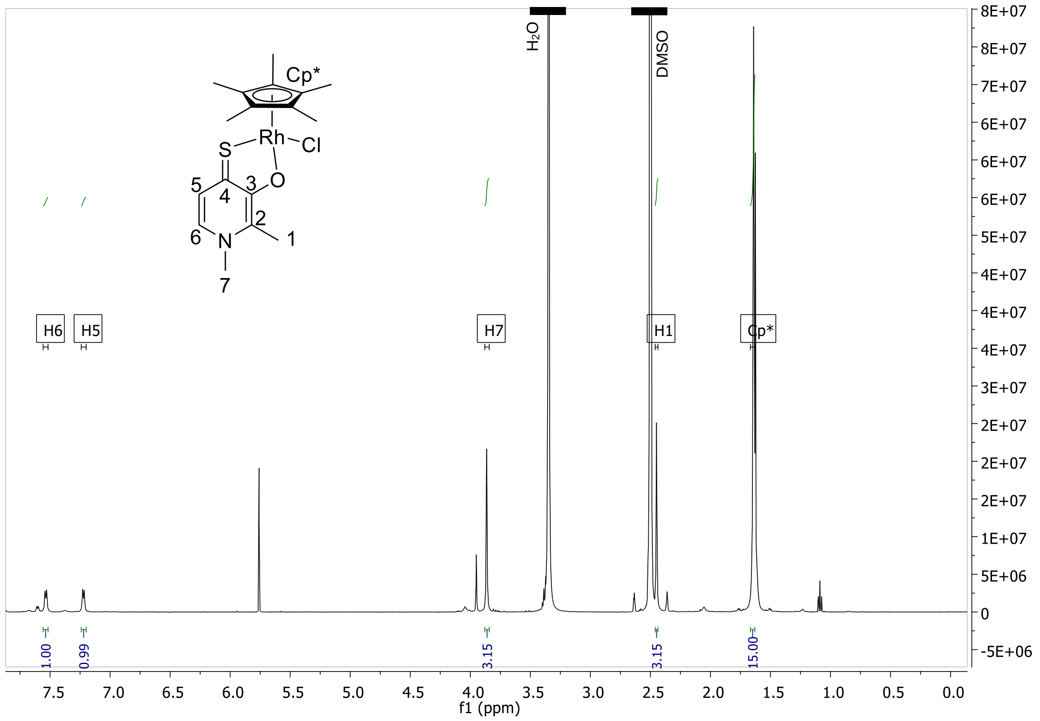

Figure S23. ^{1}H NMR of complex **5a** (500.10 MHz, d_6-DMSO, 25 °C).

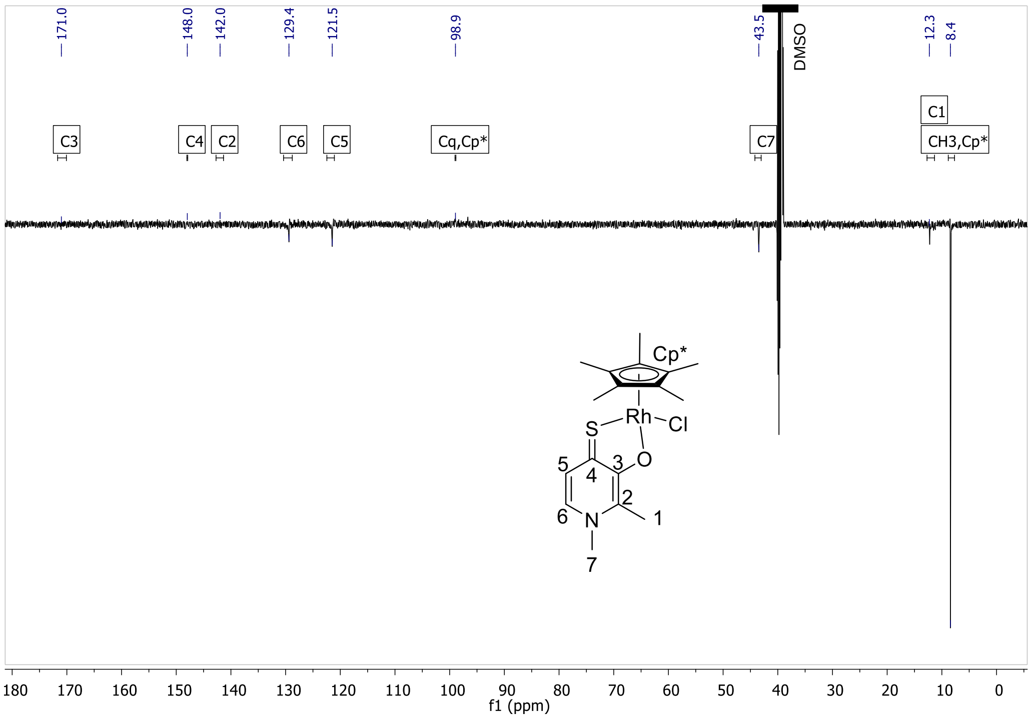

Figure S24. ^{13}C NMR of complex **5a** (125.75 MHz, d_6-DMSO, 25 °C).

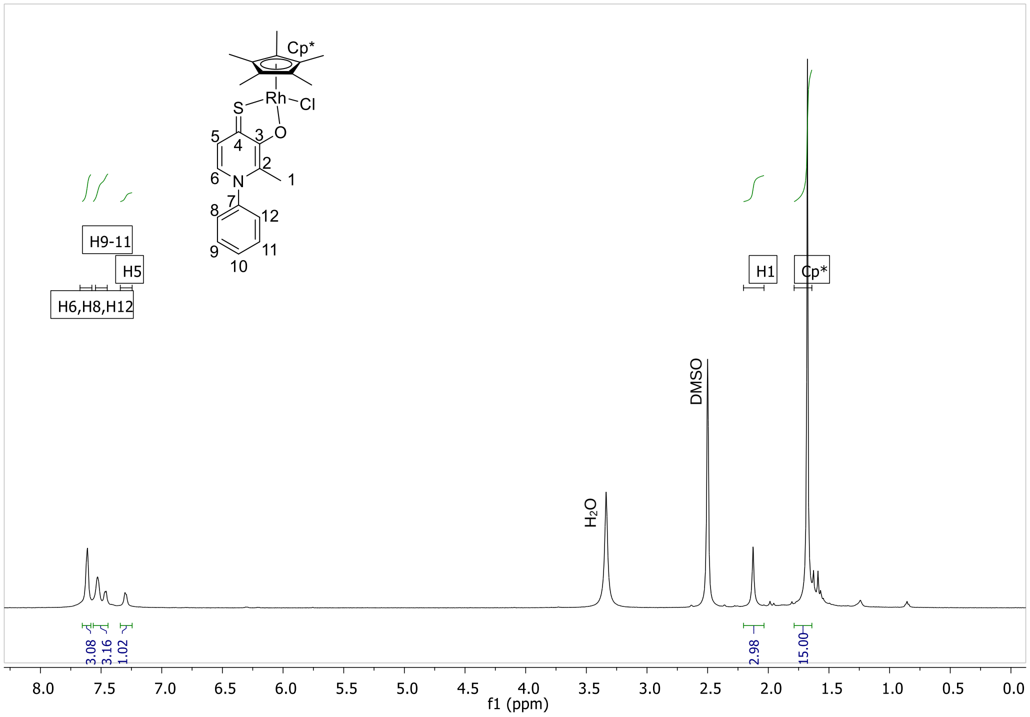

Figure S25. ^{1}H NMR of complex **5b** (500.10 MHz, d_6-DMSO, 25 °C).

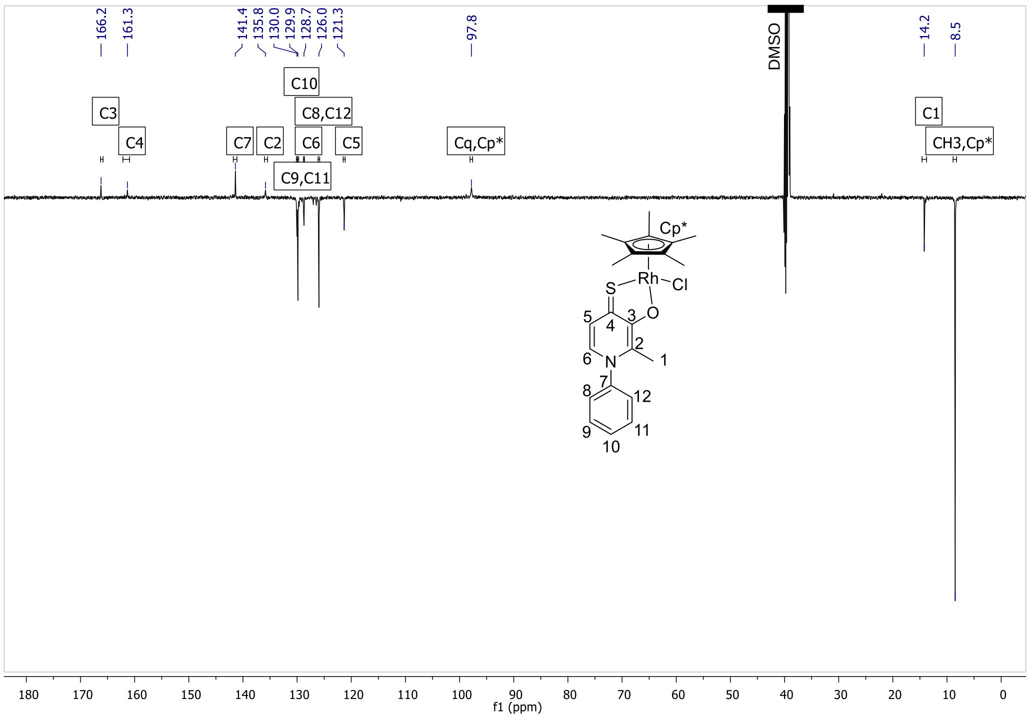

Figure S26. ^{13}C NMR of complex **5b** (125.75 MHz, d_6-DMSO, 25 °C).

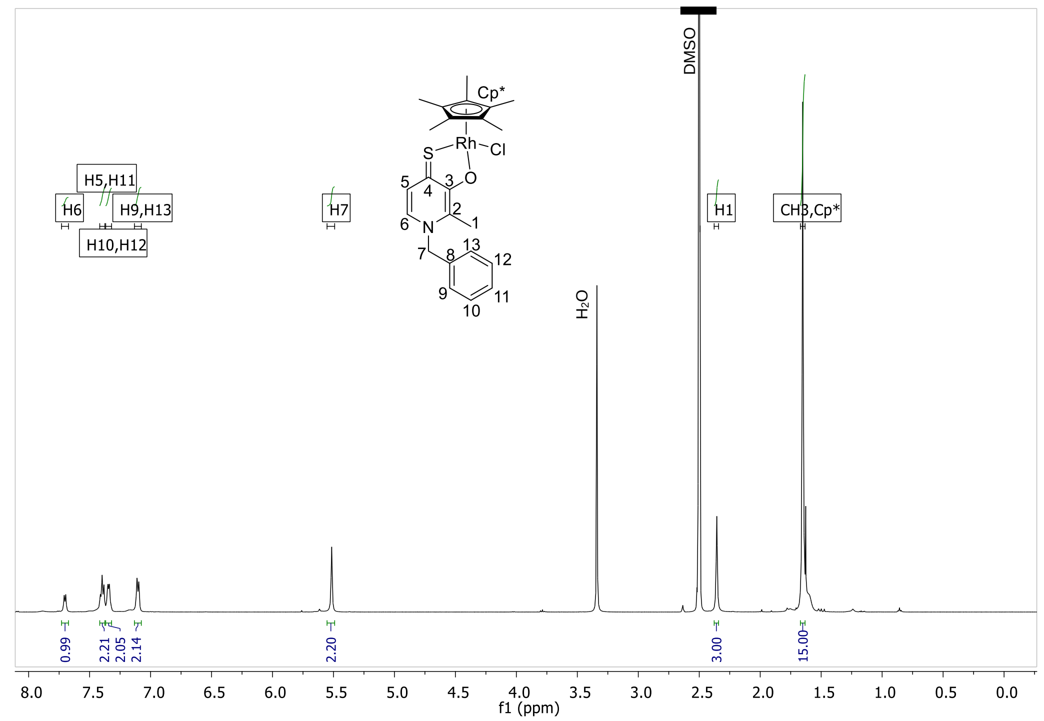

Figure S27. ^{1}H NMR of complex **5c** (500.10 MHz, d_6-DMSO, 25 °C).

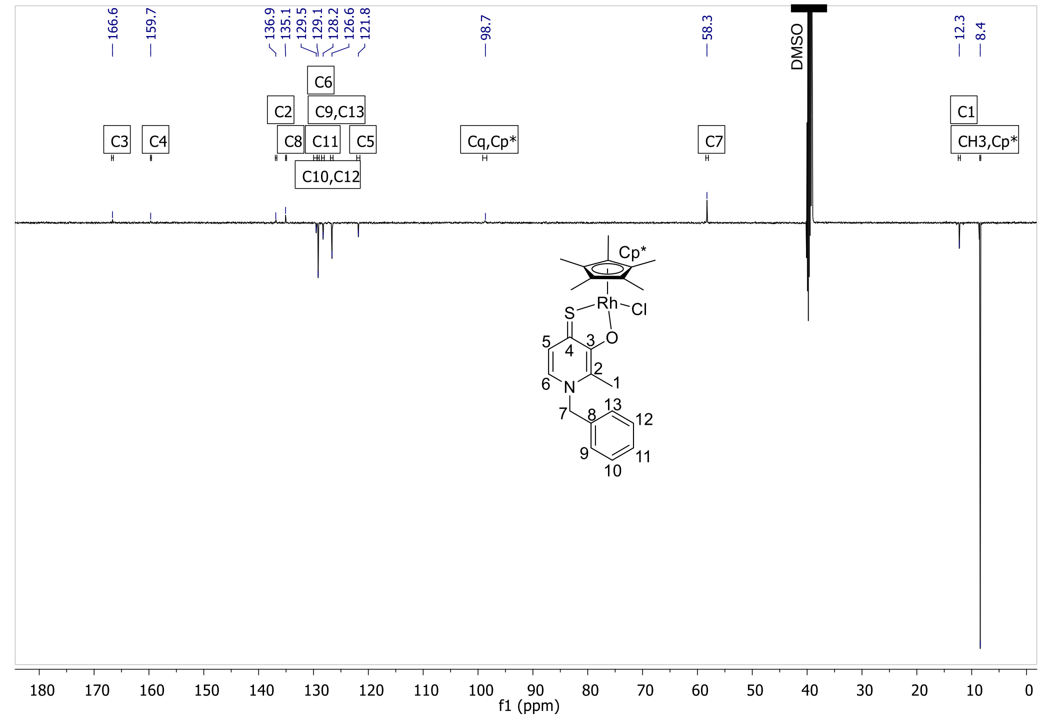

Figure S28. ^{13}C NMR of complex **5c** (125.75 MHz, d_6-DMSO, 25 °C).

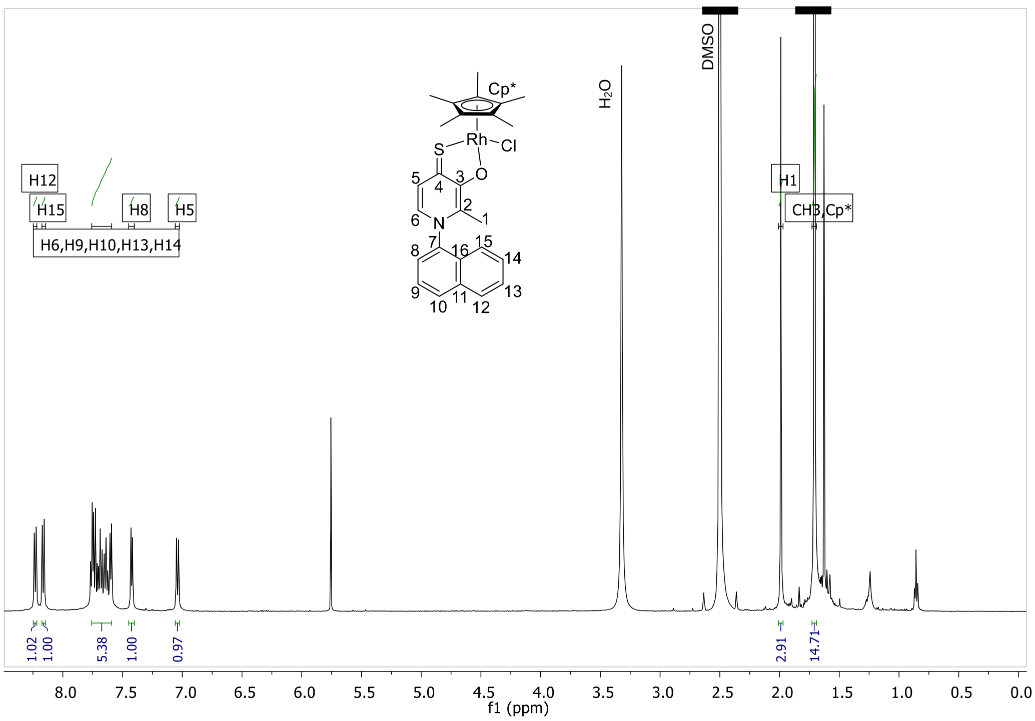

Figure S29. ^{1}H NMR of complex **5d** (500.10 MHz, d$_6$-DMSO, 25 °C).

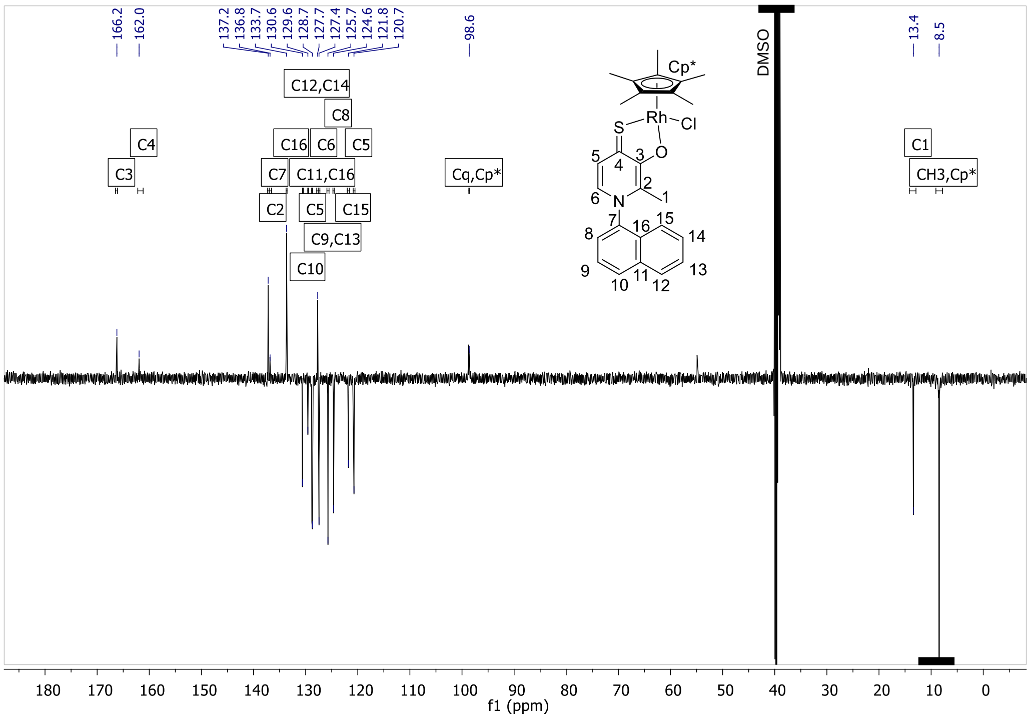

Figure S30. ^{13}C NMR of complex **5d** (125.75 MHz, d$_6$-DMSO, 25 °C).

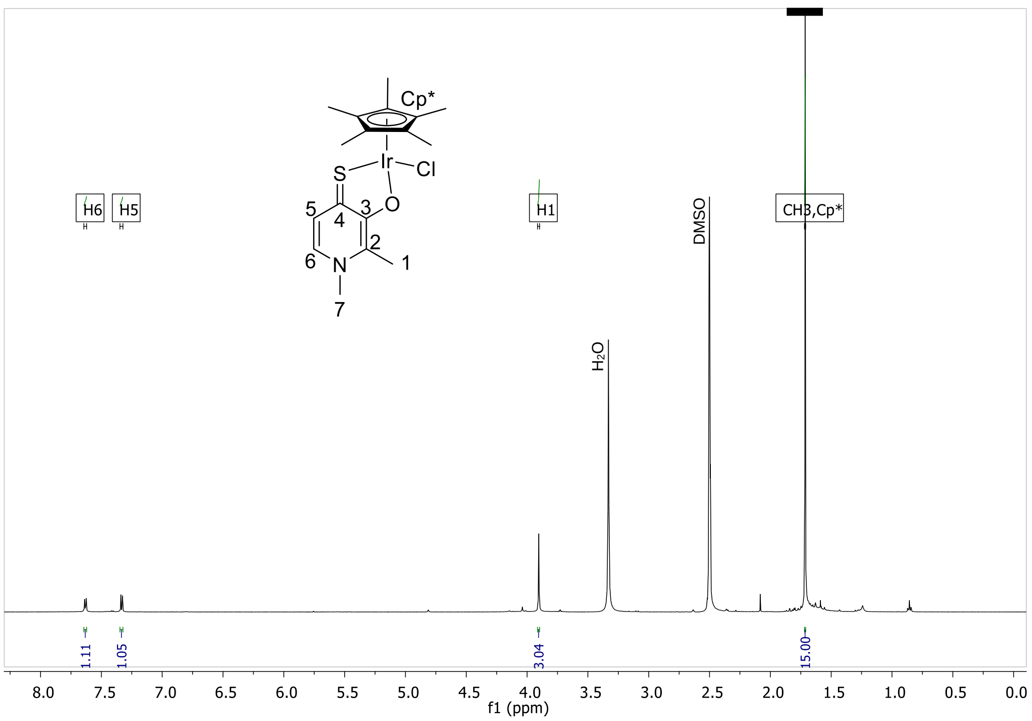

Figure S31. ^{1}H NMR of complex **6a** (500.10 MHz, d_6-DMSO, 25 °C).

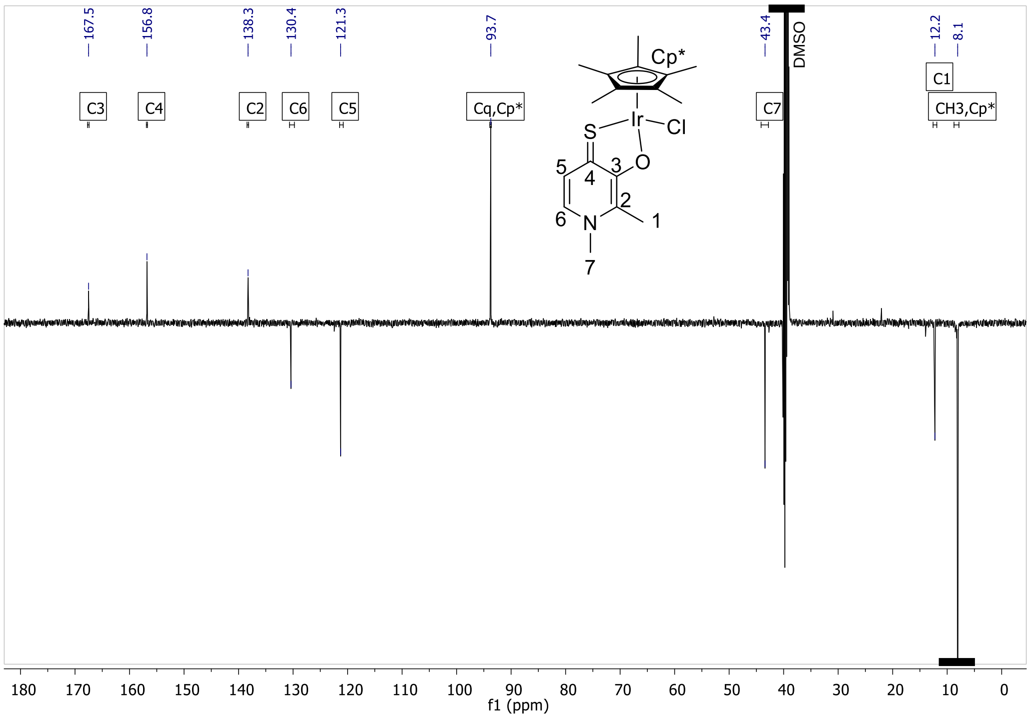

Figure S32. ^{13}C NMR of complex **6a** (125.75 MHz, d_6-DMSO, 25 °C).

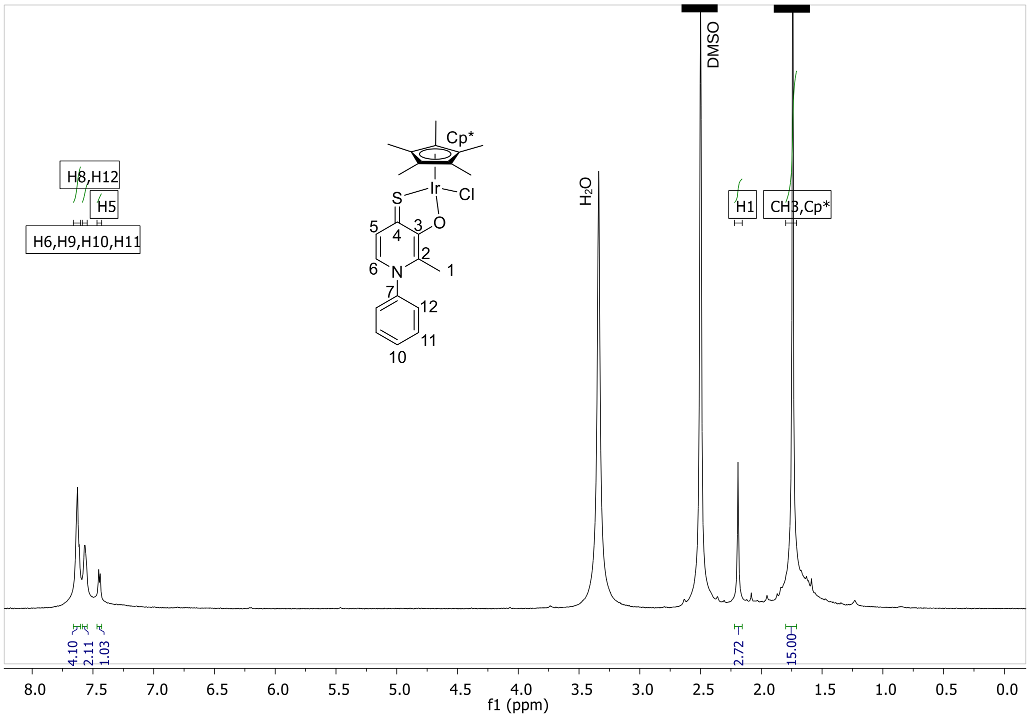

Figure S33. ^{1}H NMR of complex **6b** (500.10 MHz, d_6-DMSO, 25 °C).

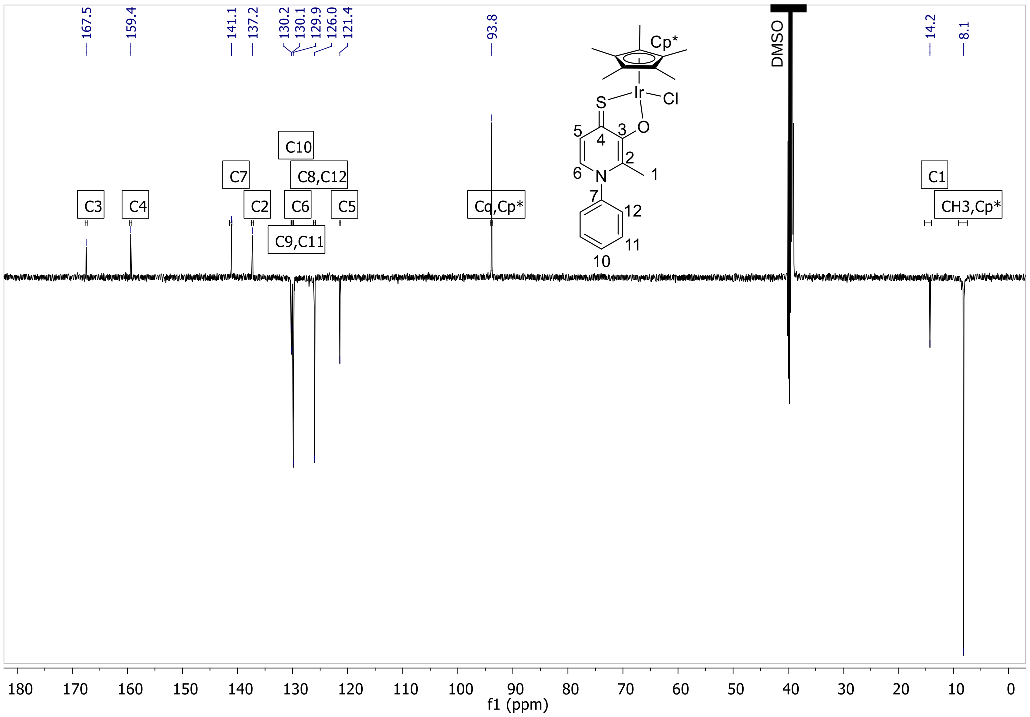

Figure S34. ^{13}C NMR of complex **6b** (125.75 MHz, d_6-DMSO, 25 °C).

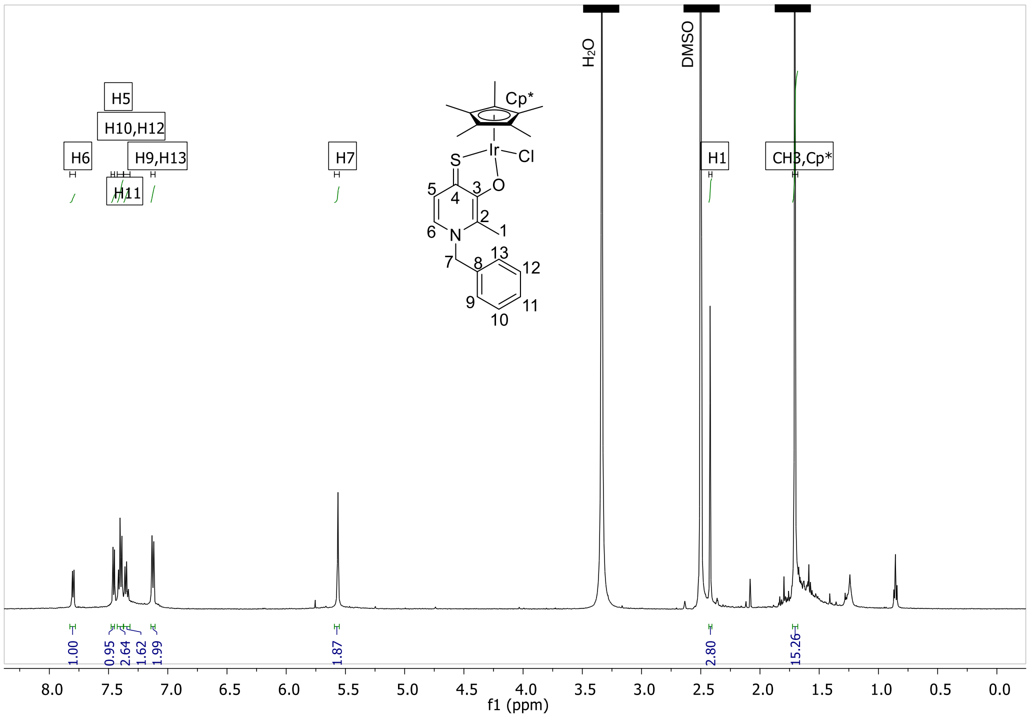

Figure S35. ^{1}H NMR of complex **6c** (500.10 MHz, d_6-DMSO, 25 °C).

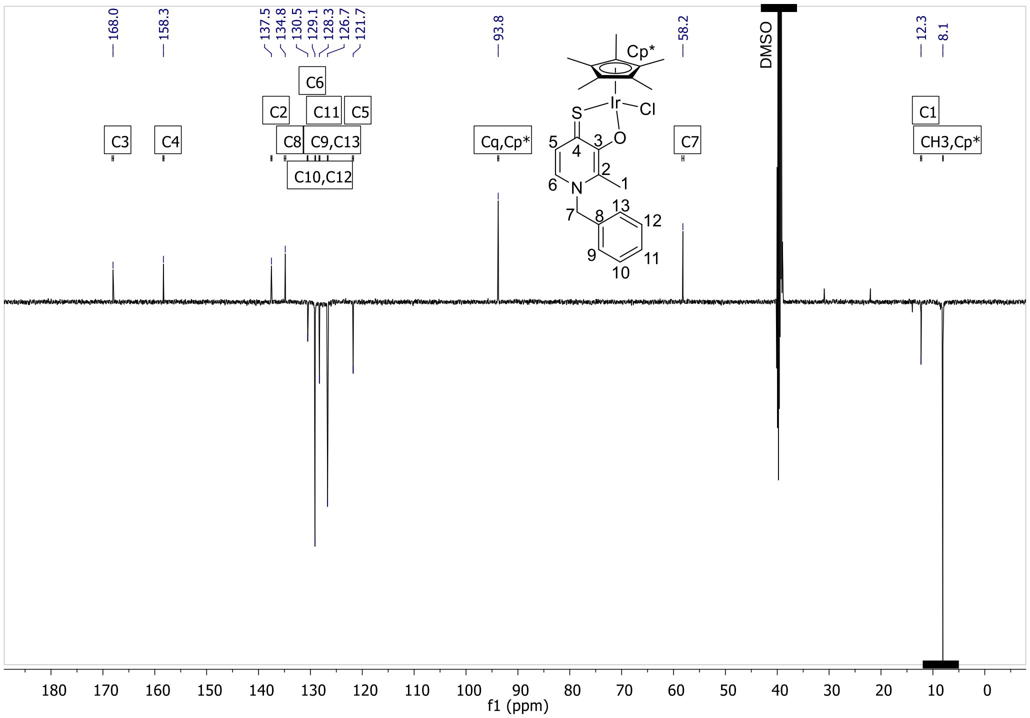

Figure S36. ^{13}C NMR of complex **6c** (125.75 MHz, d_6-DMSO, 25 °C).

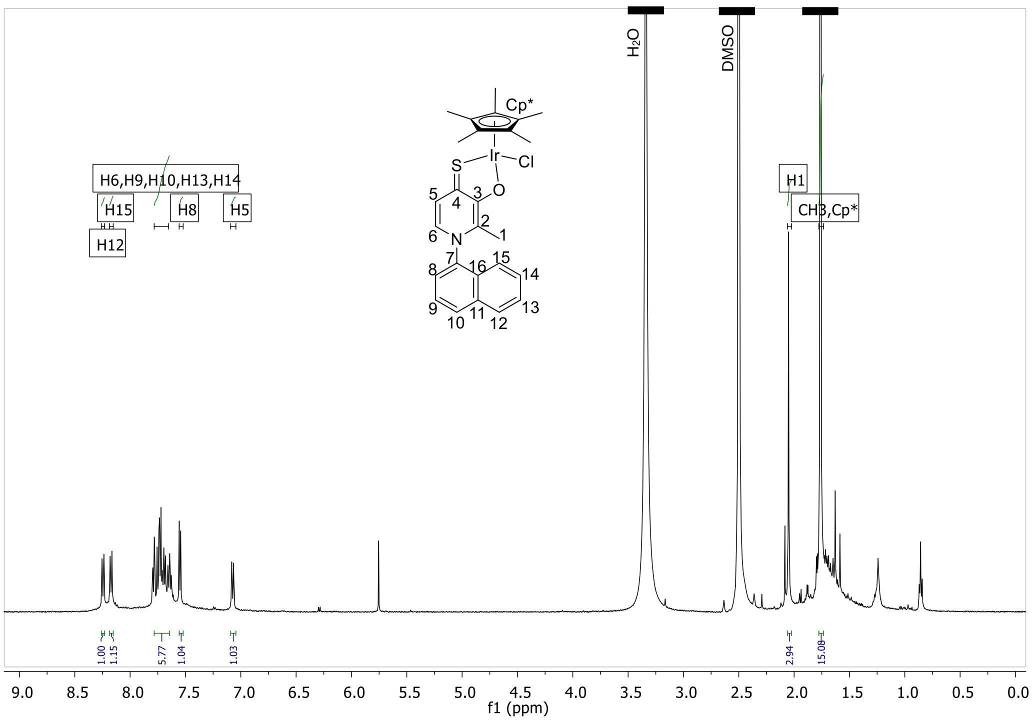

Figure S37. ^{1}H NMR of complex **6d** (500.10 MHz, d_6-DMSO, 25 °C).

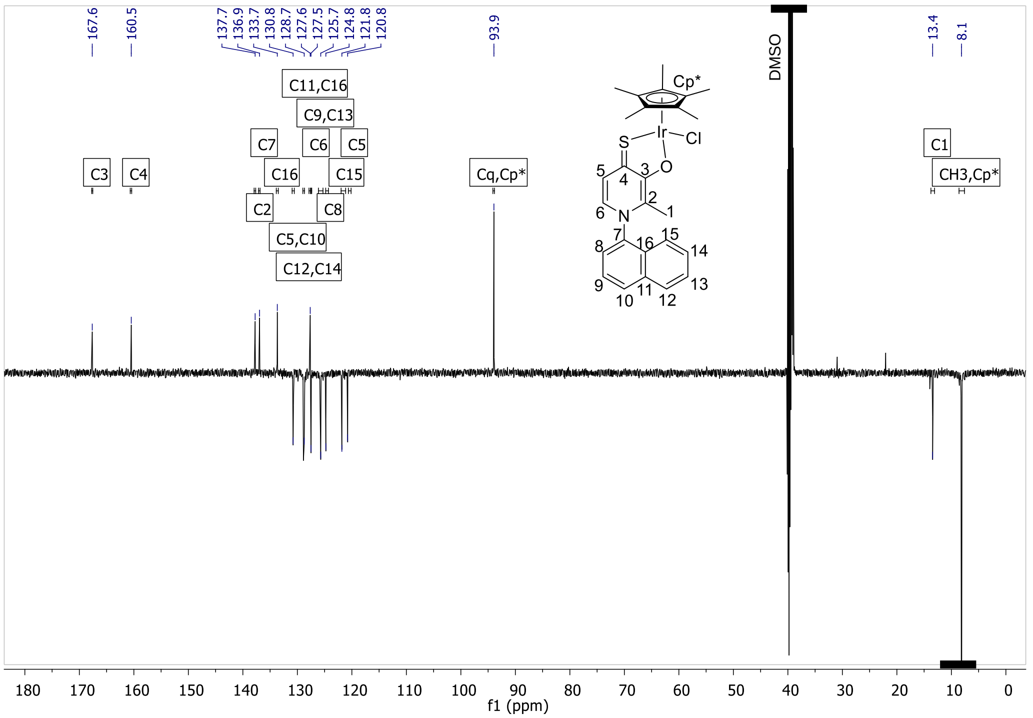

Figure S38. ^{13}C NMR of complex **6d** (125.75 MHz, d_6-DMSO, 25 °C).

NMR Spectra in D_2O (Charges omitted for clarity)

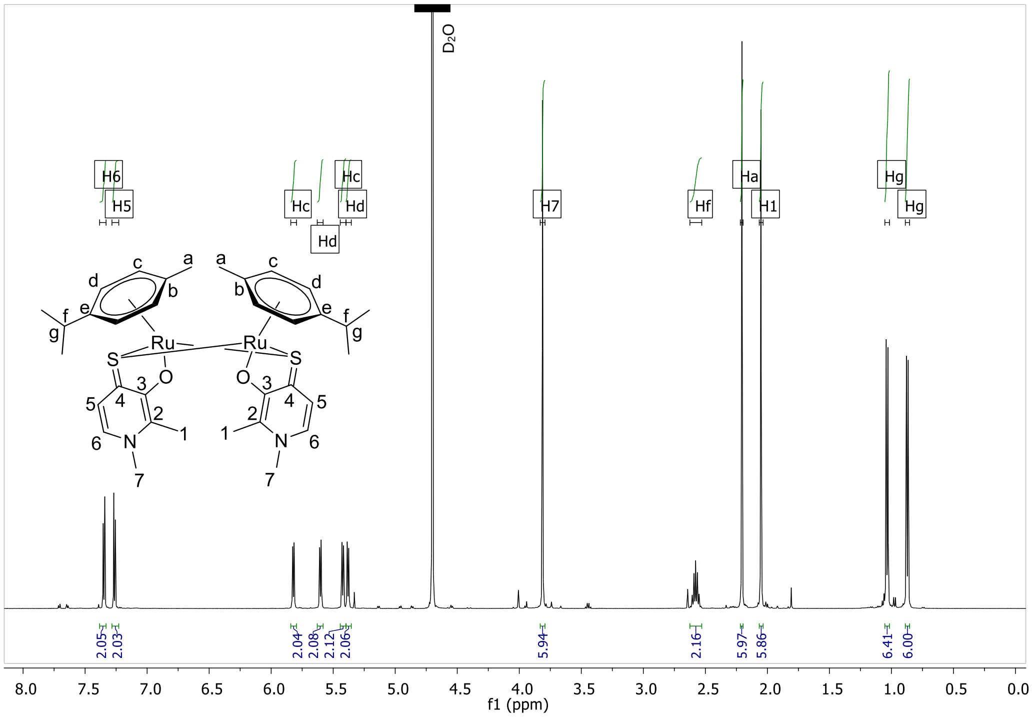

Figure S39. ^{1}H NMR of complex **3a*** (500.10 MHz, D_2O, 25 °C).

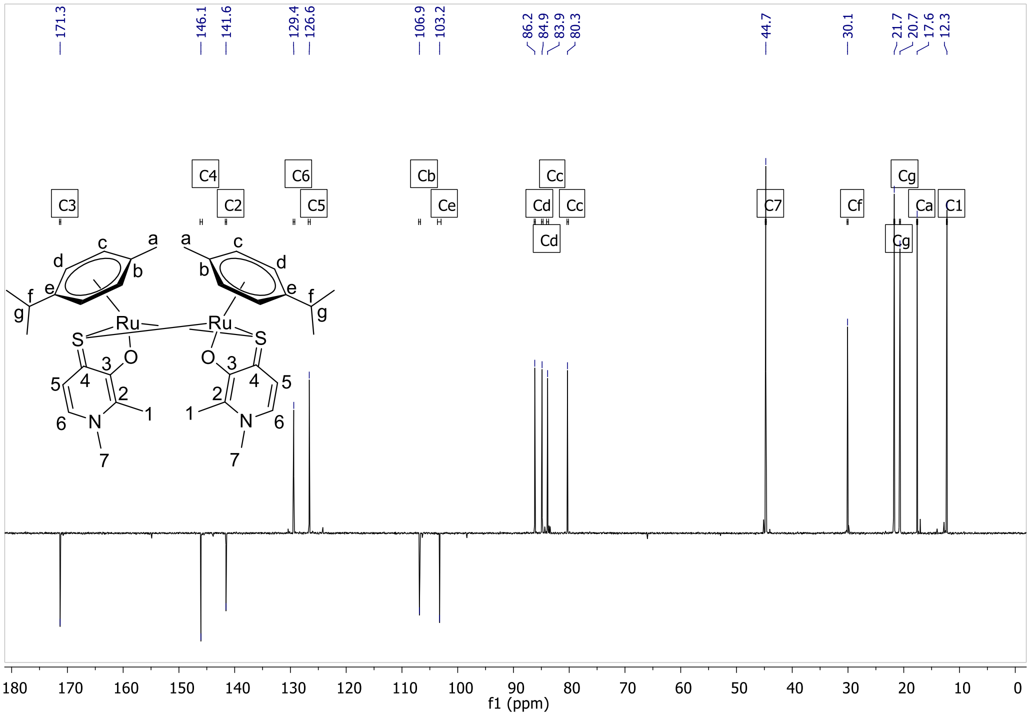

Figure S 40. ^{13}C NMR of complex **3a*** (125.75 MHz, D_2O, 25 °C).

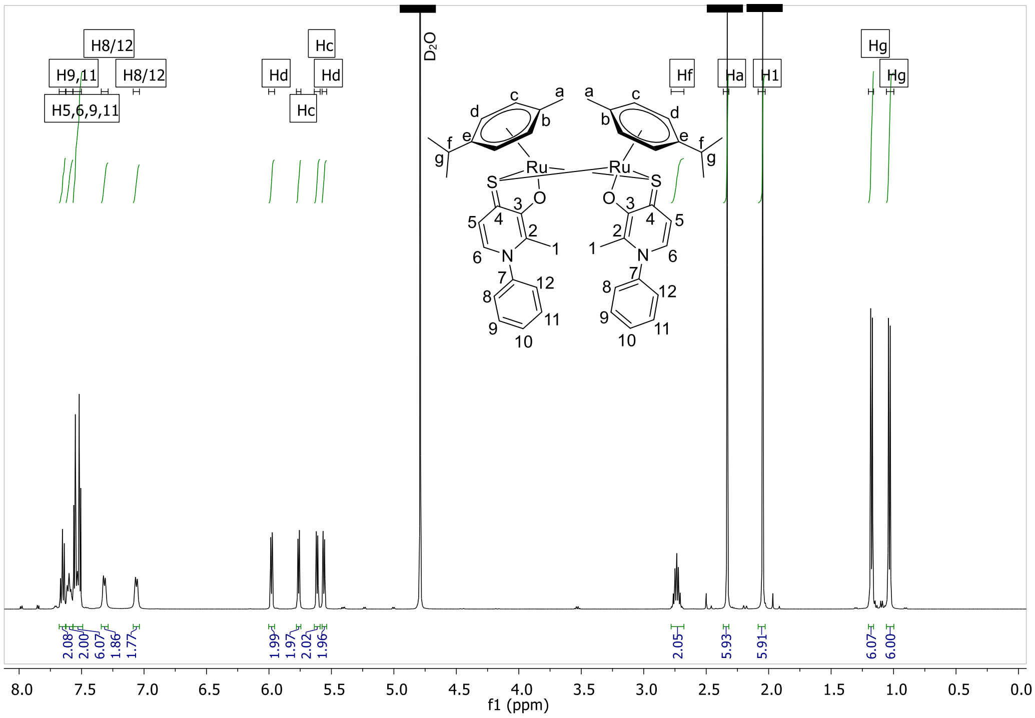

Figure S41. ^{1}H NMR of complex **3b*** (500.10 MHz, D_2O, 25 °C).

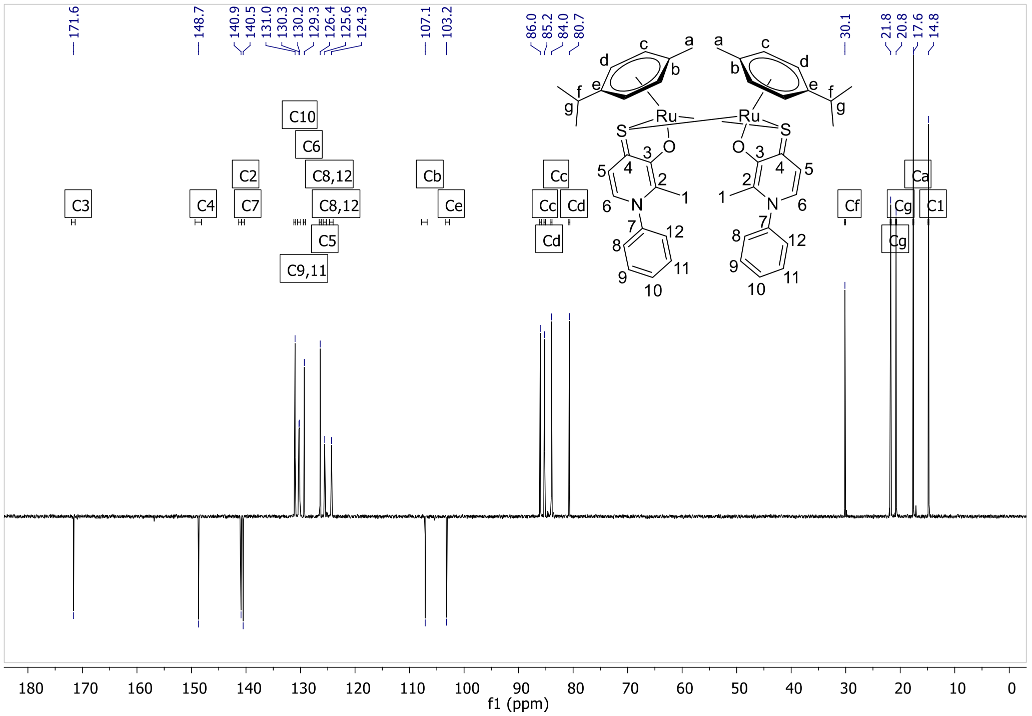

Figure S42. ^{13}C NMR of complex **3b*** (125.75 MHz, D_2O, 25 °C).

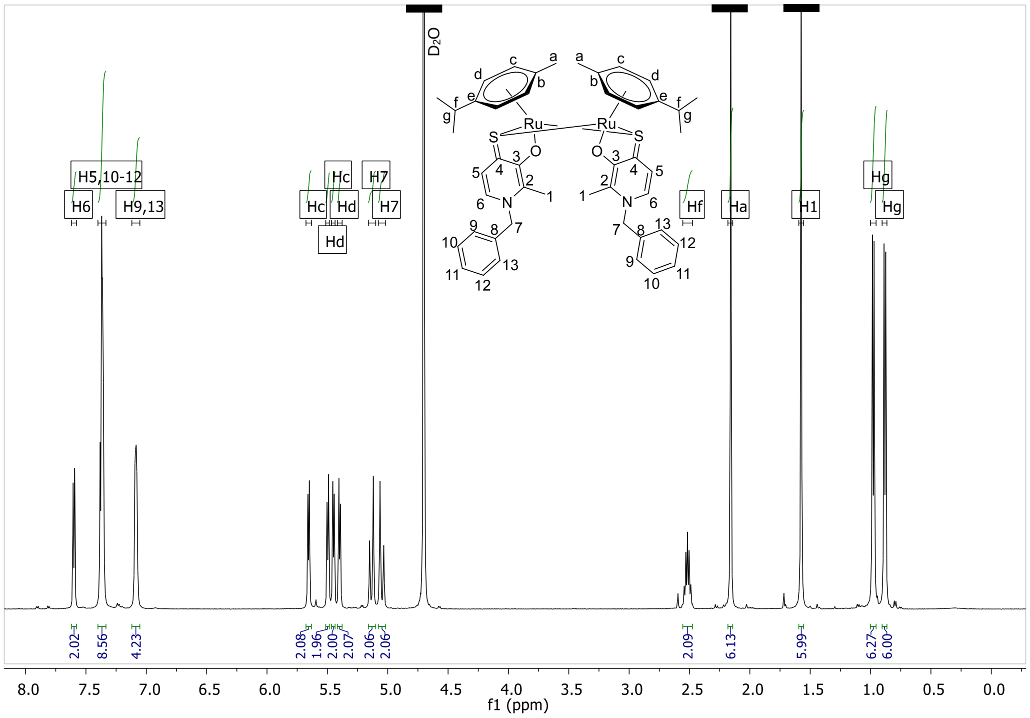

Figure S43. ^{1}H NMR of complex **3c*** (500.10 MHz, D_2O, 25 °C).

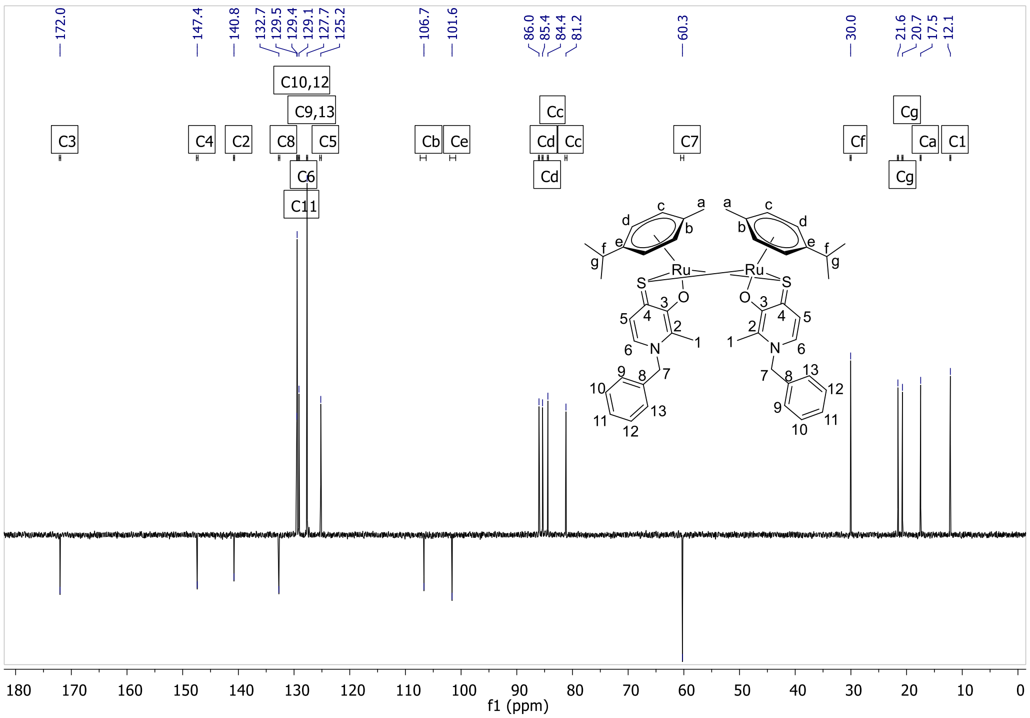

Figure S44. ^{13}C NMR of complex **3c*** (125.75 MHz, D_2O, 25 °C).

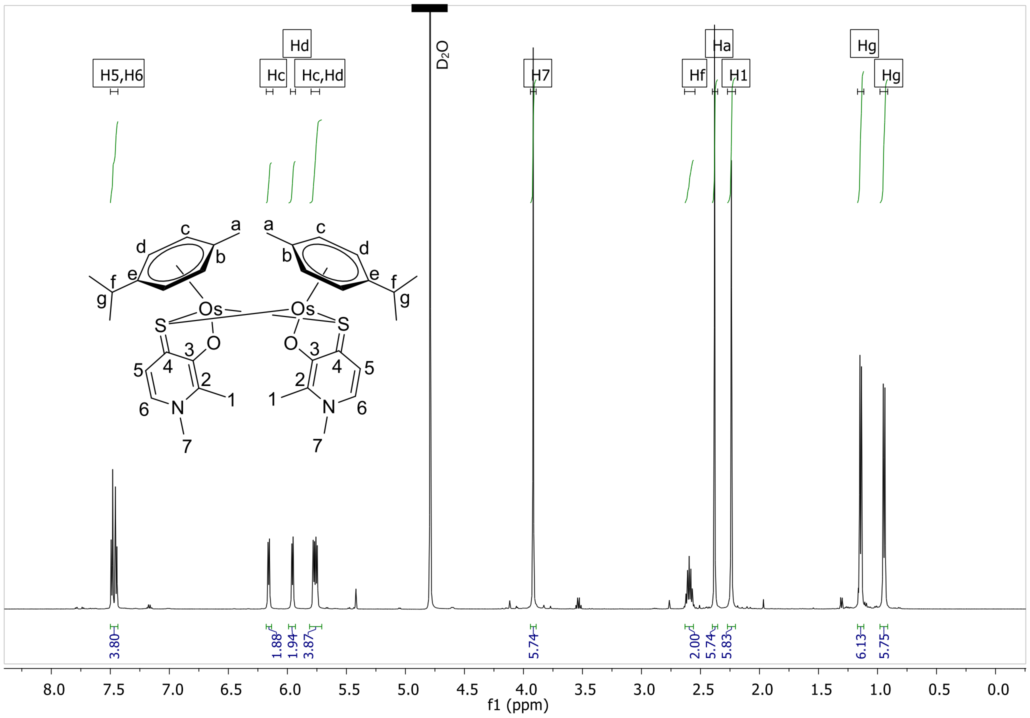

Figure S45. ^{1}H NMR of complex **4a*** (500.10 MHz, D_2O, 25 °C).

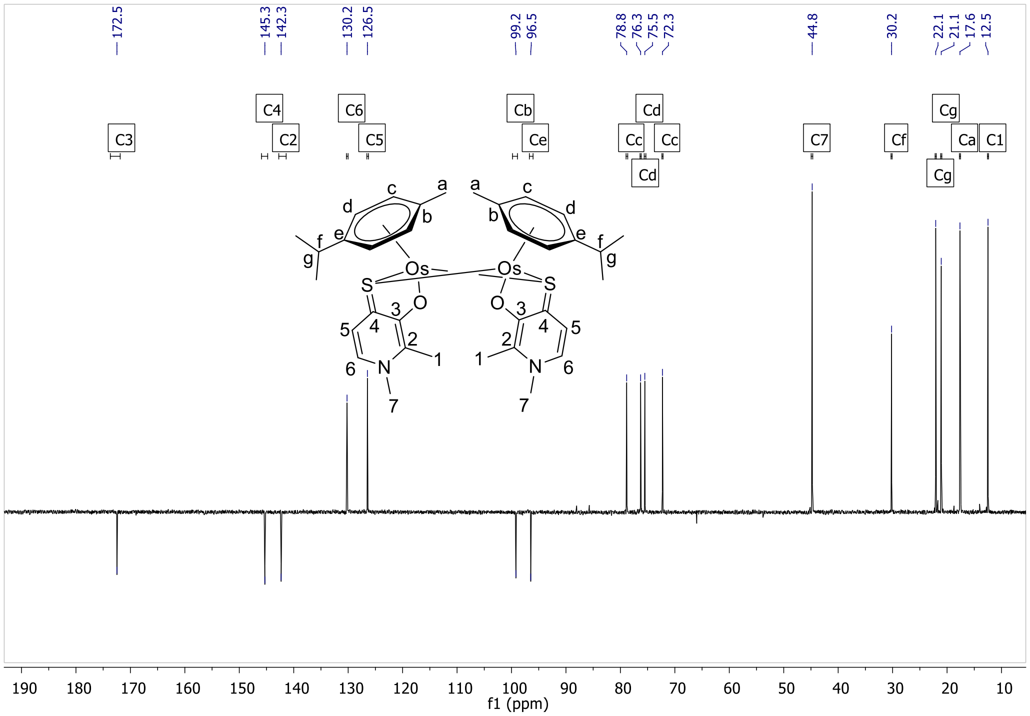

Figure S46. ^{13}C NMR of complex **4a*** (125.75 MHz, D_2O, 25 °C).

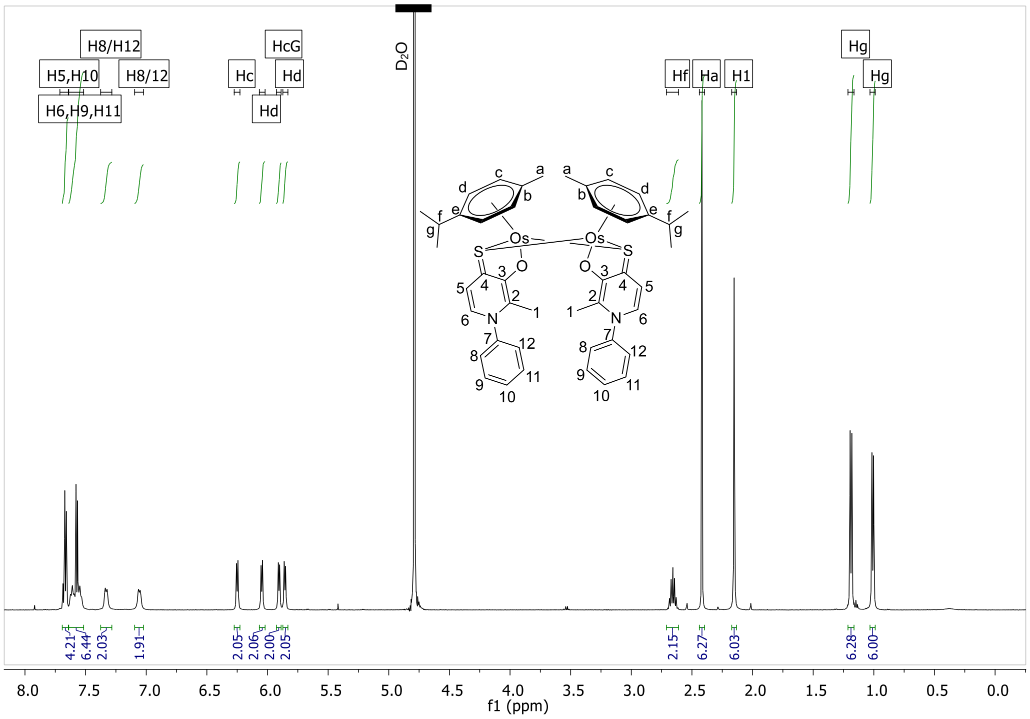

Figure S47. ^{1}H NMR of complex **4b*** (500.10 MHz, D_2O, 25 °C).

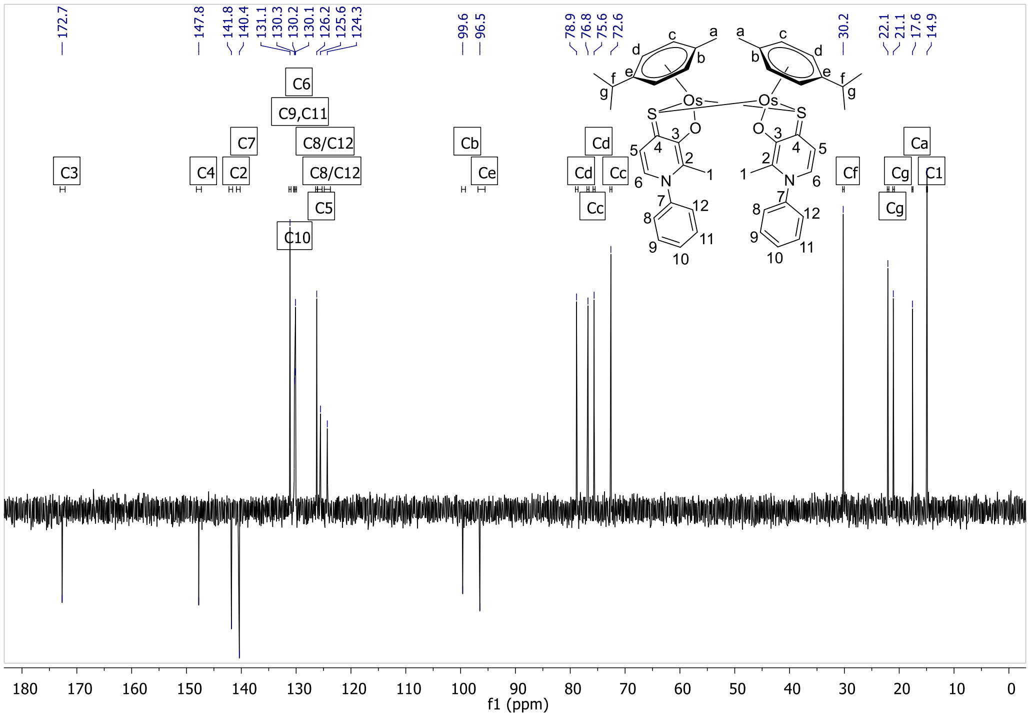

Figure S48. ^{13}C NMR of complex **4b*** (125.75 MHz, D_2O, 25 °C).

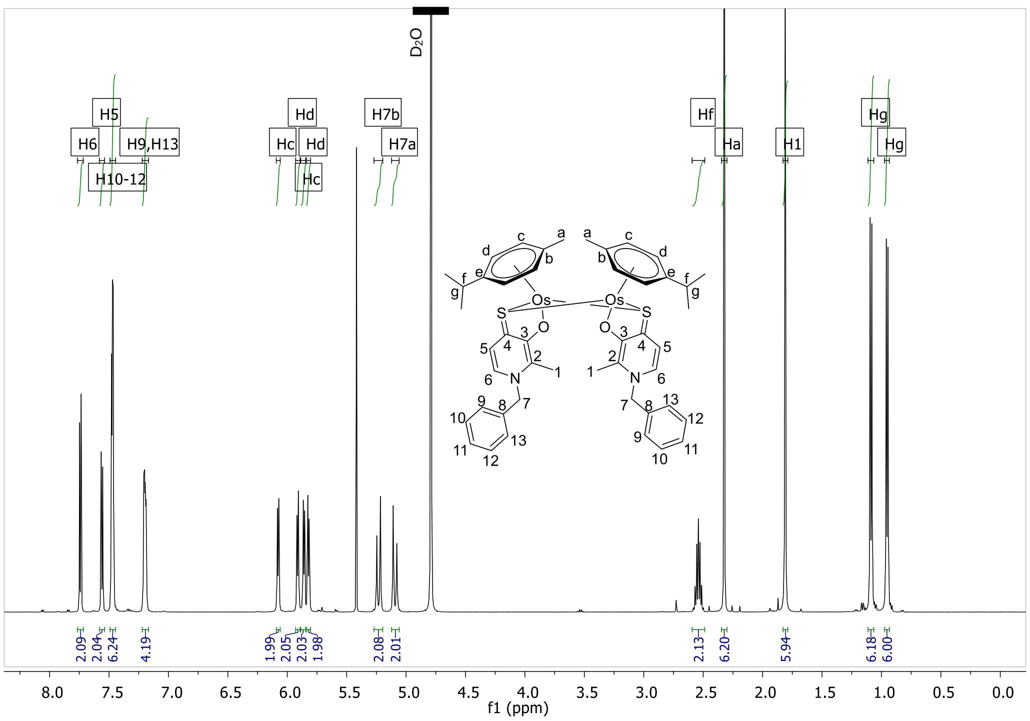

Figure S49. ^{1}H NMR of complex **4c*** (500.10 MHz, D_2O, 25 °C).

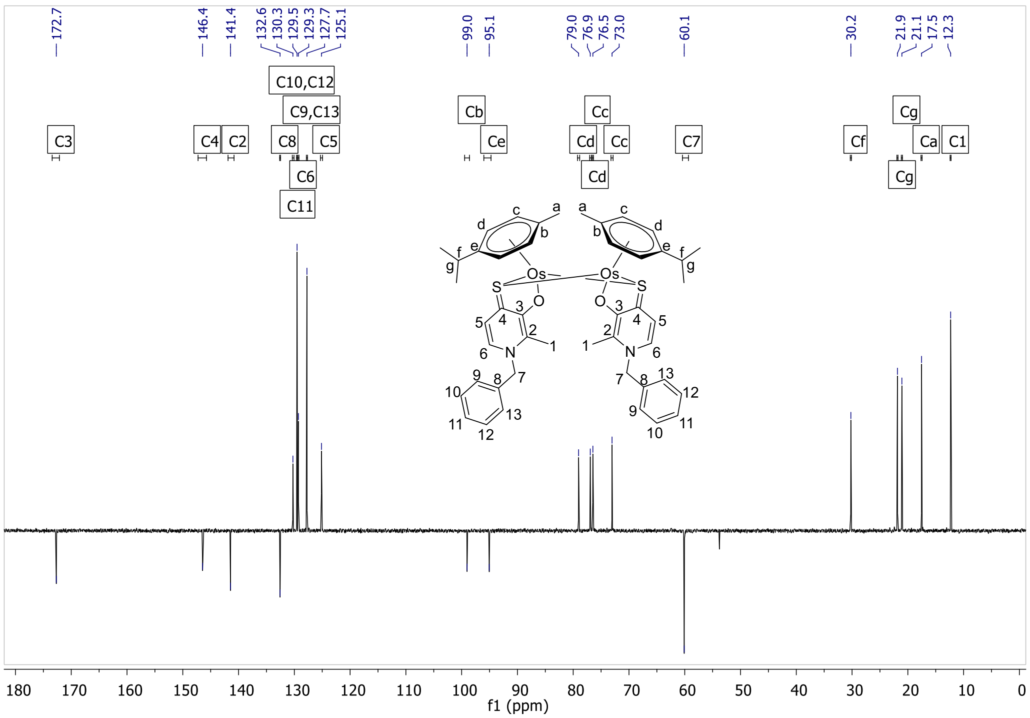

Figure S50. ^{13}C NMR of complex **4c*** (125.75 MHz, D_2O, 25 °C).

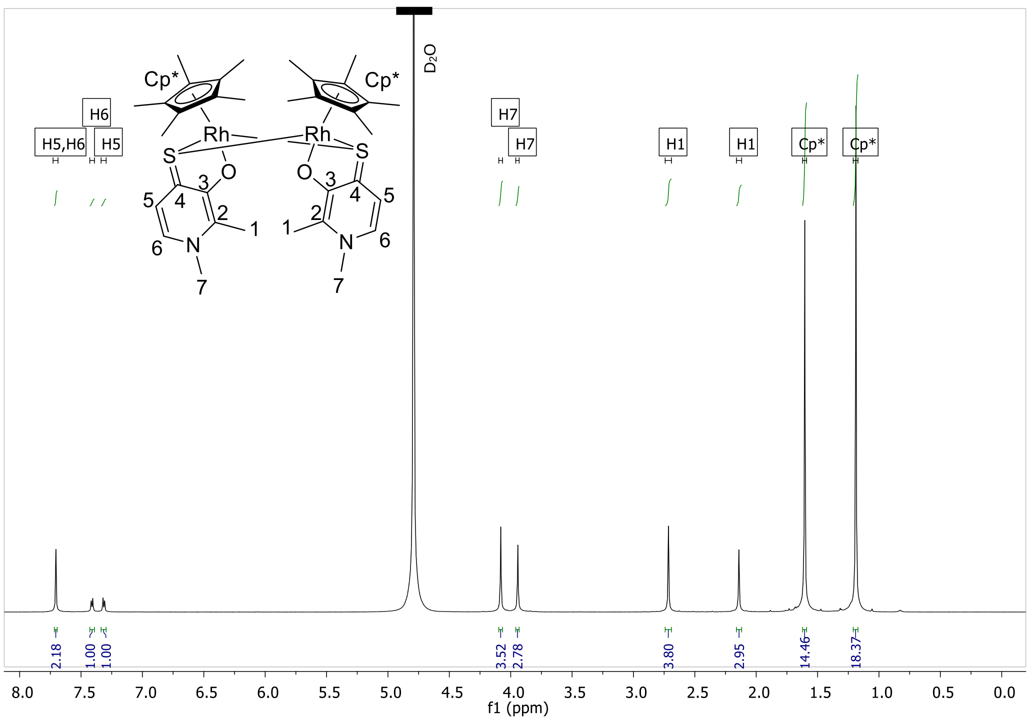

Figure S51. ^{1}H NMR of complex **5a*** (500.10 MHz, D_2O, 25 °C).

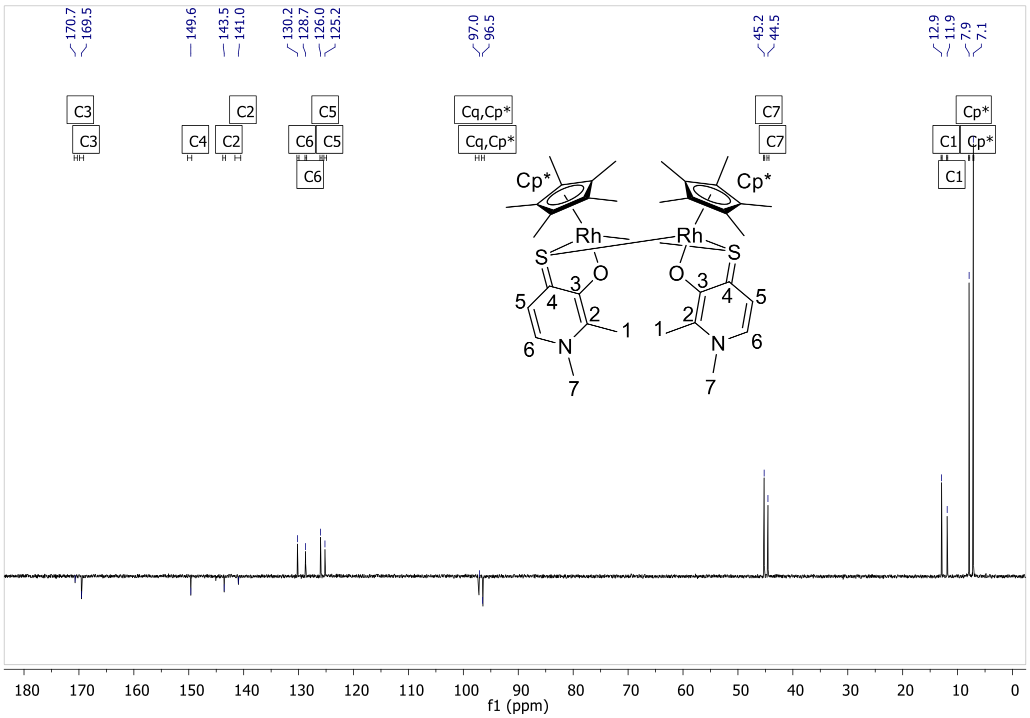

Figure S52. ^{13}C NMR of complex **5a*** (125.75 MHz, D_2O, 25 °C).

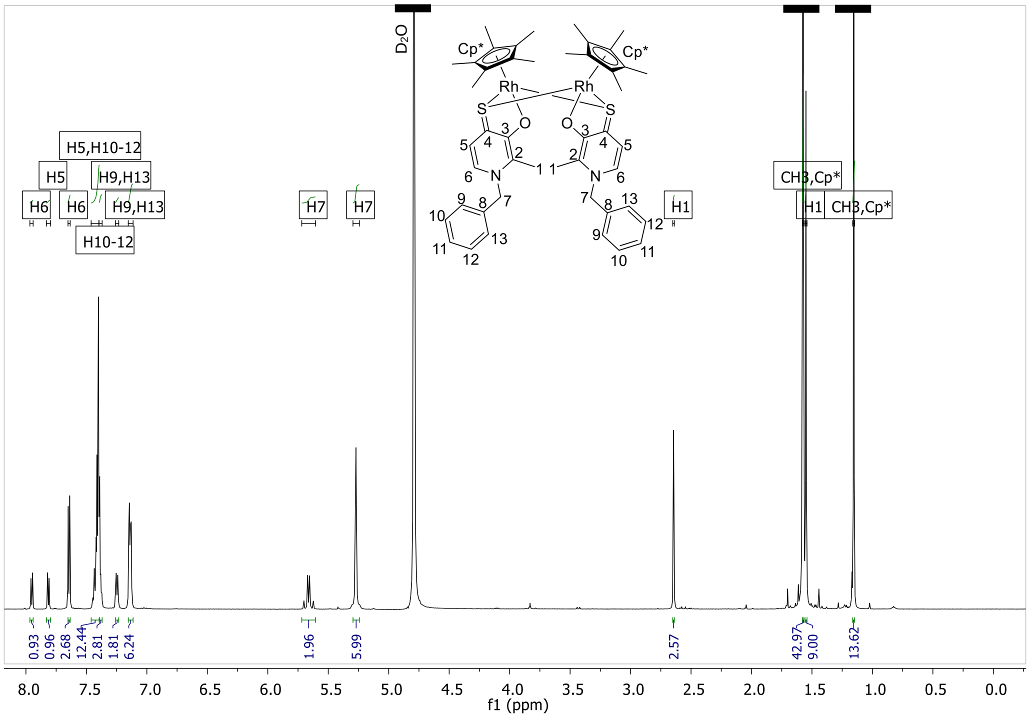

Figure S53. ^{1}H NMR of complex **5c*** (500.10 MHz, D_2O, 25 °C).

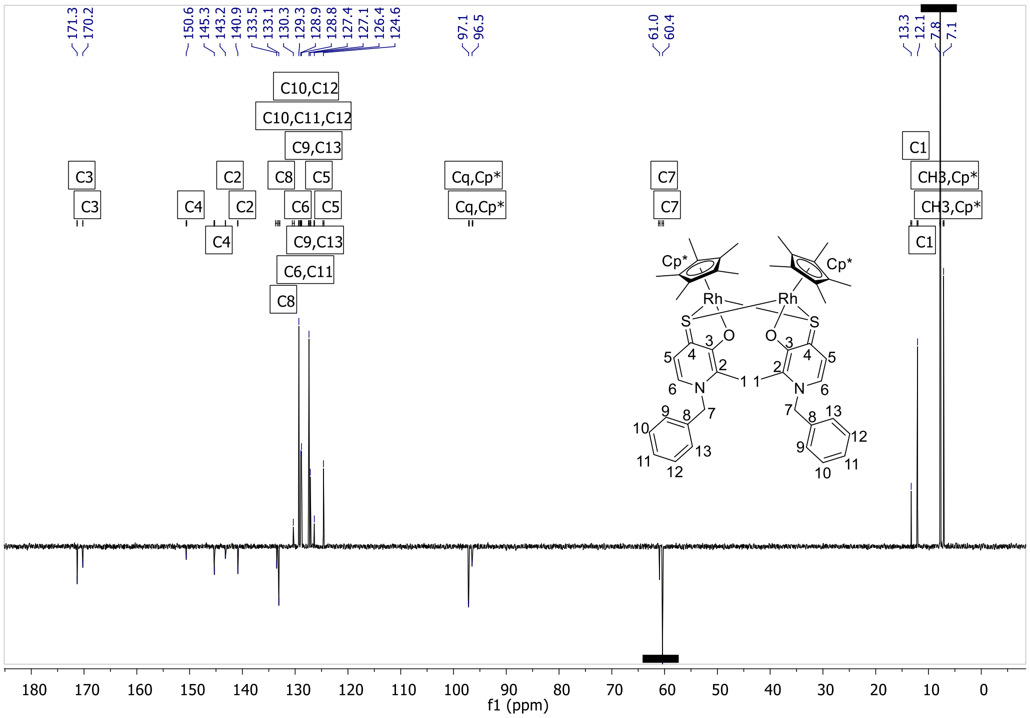

Figure S54. ^{13}C NMR of complex **5c*** (125.75 MHz, D_2O, 25 °C).

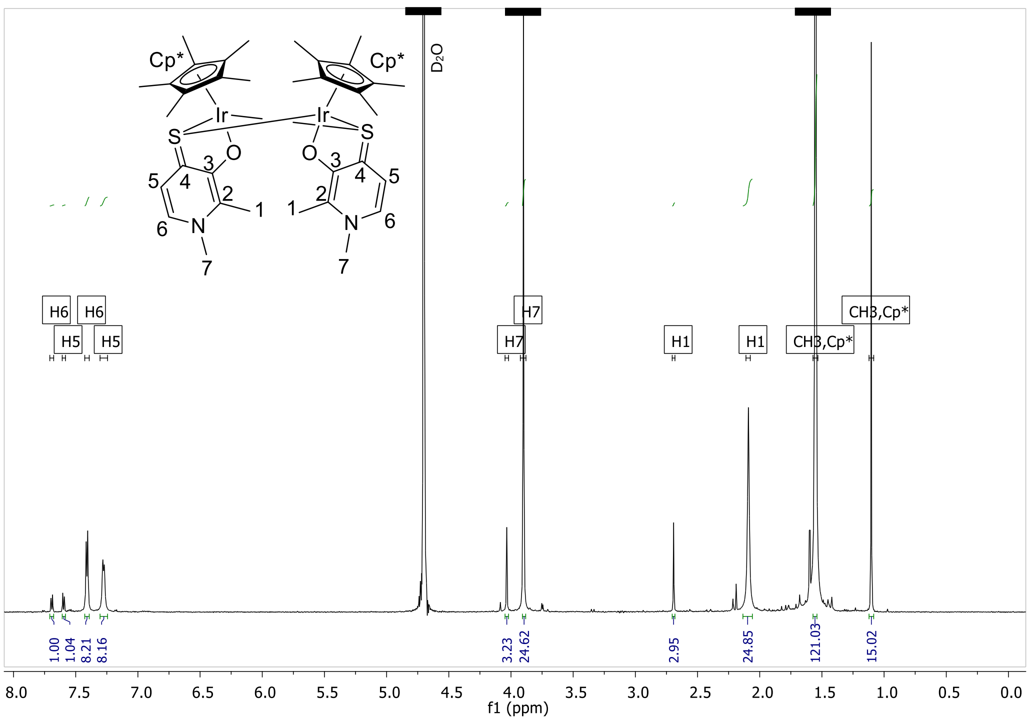

Figure S55. ^{1}H NMR of complex **6a*** (500.10 MHz, D_2O, 25 °C).

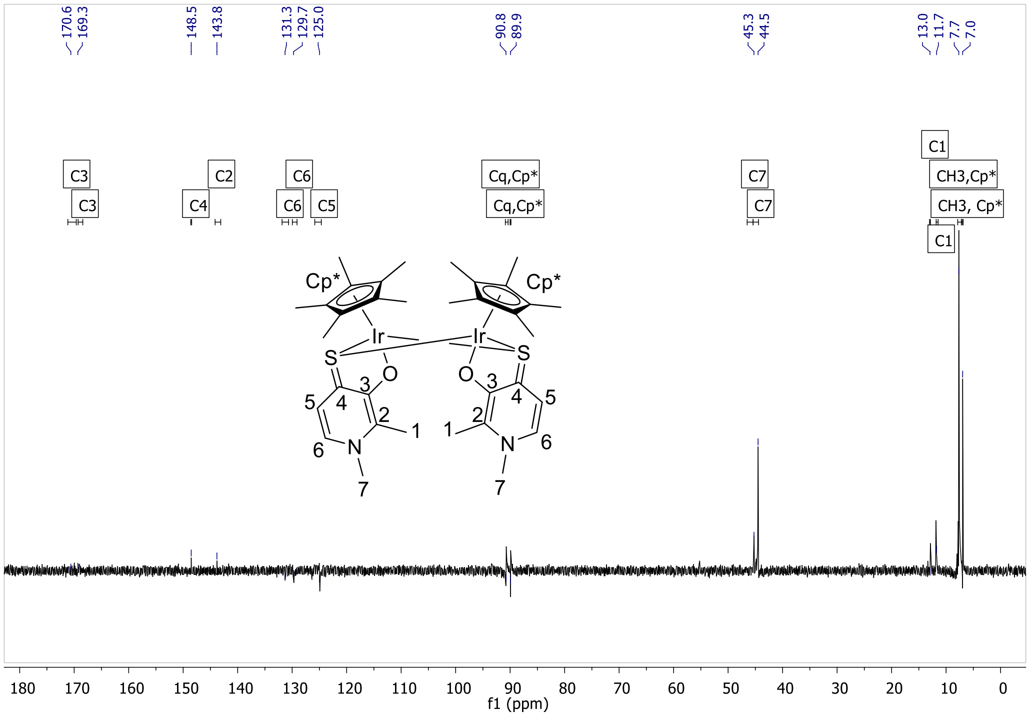

Figure S56. ^{13}C NMR of complex **6a*** (125.75 MHz, D_2O, 25 °C).

X-ray diffraction analysis

Table S1. Experimental parameter and CCDC-Codes.

Compound	Machine	Source	Temp.	Detector Distance	Time/ Frame	#Frames	Frame width	CCDC
	Bruker		[K]	[mm]	[s]		[°]	
**2b**	X8	Mo	130	35	5	851	0.500	1959932
**2c**	X8	Mo	100	35	10	4277	0.500	1959933
**2d**	X8	Mo	100	35	10	1067	0.500	1959938
**3a***	D8	Mo	100	30	100	521	0.500	1959930
**3b**	X8	Mo	130	35	5	850	0500	1959934
**3b***	X8	Mo	130	35	3	1108	0.500	1959936
**3c**	X8	Mo	130	35	4	961	0.500	1959935
**3c***	X8	Mo	130	35	7	362	0.500	1959943
**3d**	X8	Mo	100	35	3	1109	0.500	1959940
**4a**	D8	Mo	100	30	20	496	0.500	1959931
**4a***	D8	Mo	100	30	2	2034	0.500	1959927
**4b**	X8	Mo	100	35	10	1067	0.500	1959937
**4c**	X8	Mo	130	35	3	558	0.500	1959939
**4d**	X8	Mo	100	35	3	1185	0.500	1959941
**5b***	X8	Mo	100	35	16	739	0.500	1959942
**6b***	D8	Mo	100	30	20	665	0.500	1959929
**6d**	D8	Mo	150	35	12	802	0.500	1959928

UV-Vis

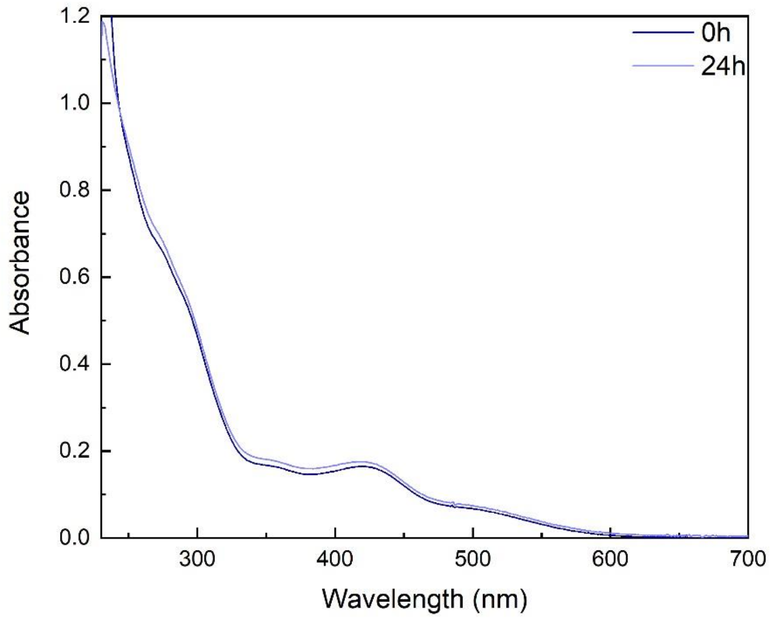

Figure S57. UV/Vis stability measurements of complex **3a** in H_2O over 24h at 25 °C.

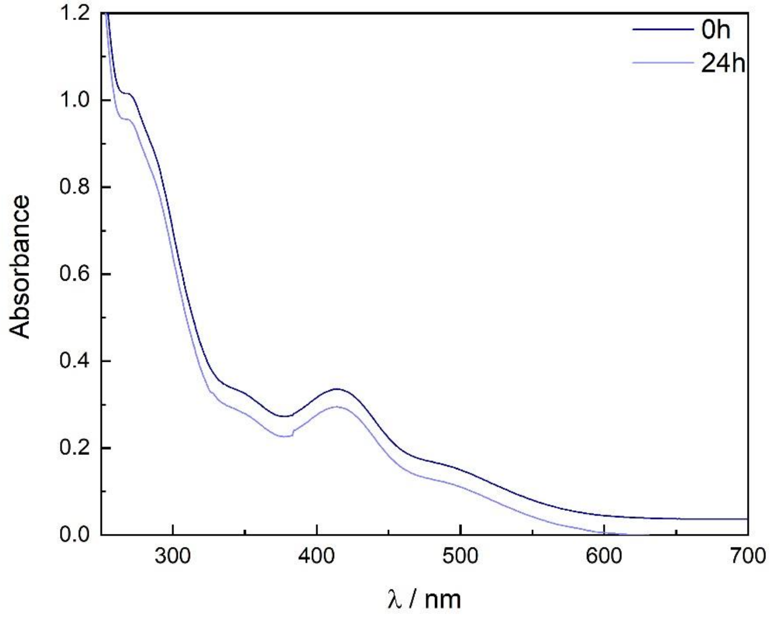

Figure S58. UV/Vis stability measurements of complex **3b** in H_2O over 24h at 25 °C.

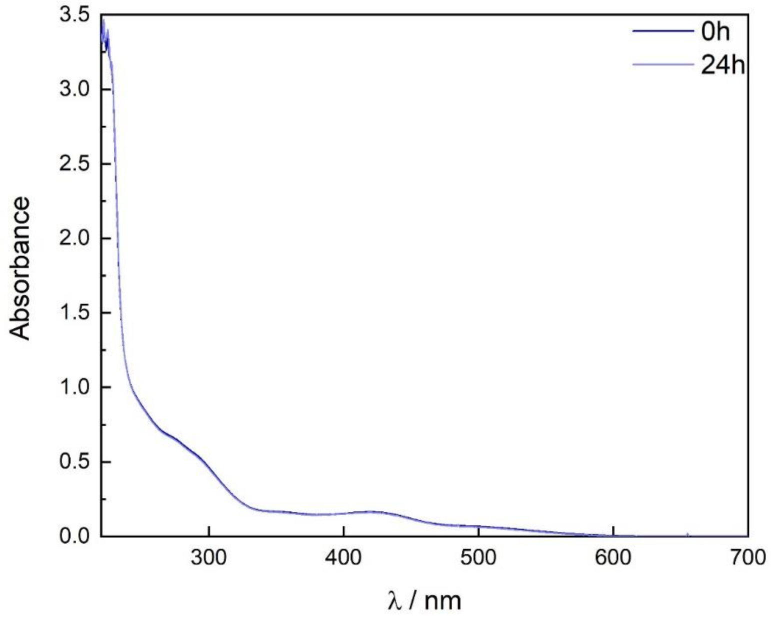

Figure S59. UV/Vis stability measurements of complex **3c** in H_2O over 24h at 25 °C.

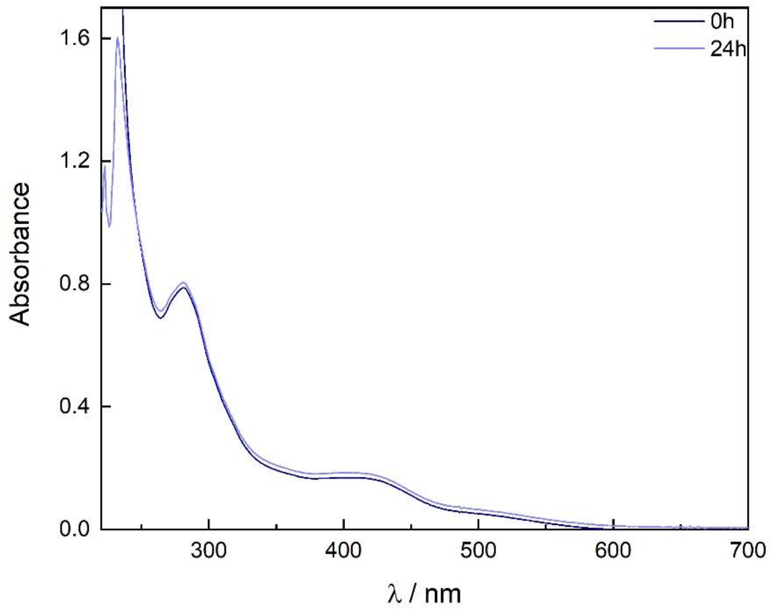

Figure S60. UV/Vis stability measurements of complex **3d** in H_2O over 24h at 25 °C.

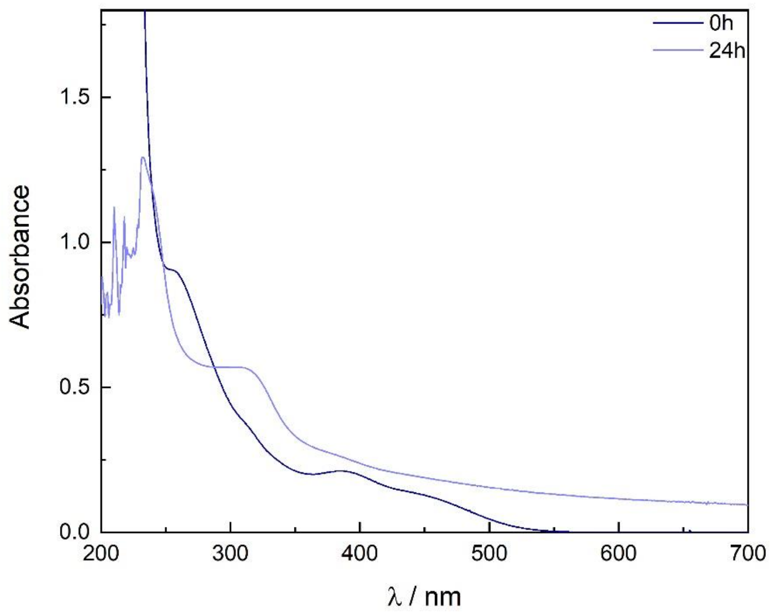

Figure S61. UV/Vis stability measurements of complex **4a** in H_2O over 24h at 25 °C.

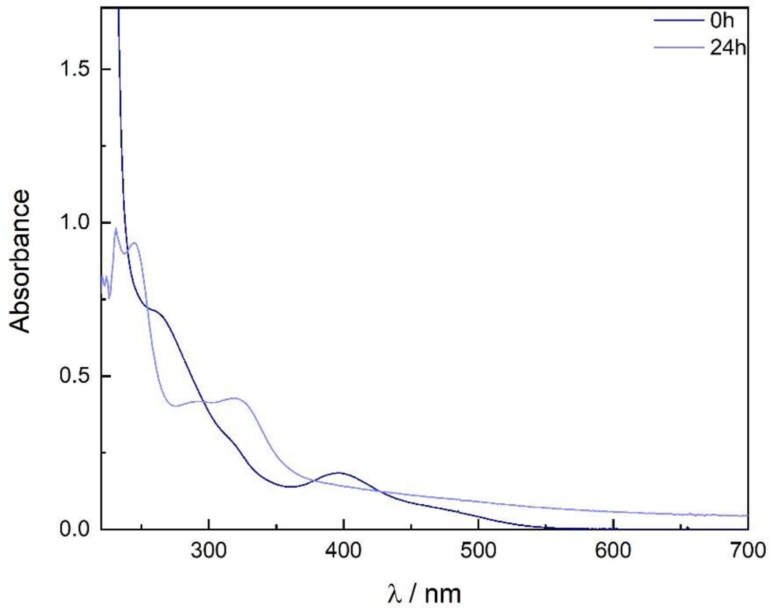

Figure S62. UV/Vis stability measurements of complex **4b** in H_2O over 24h at 25 °C.

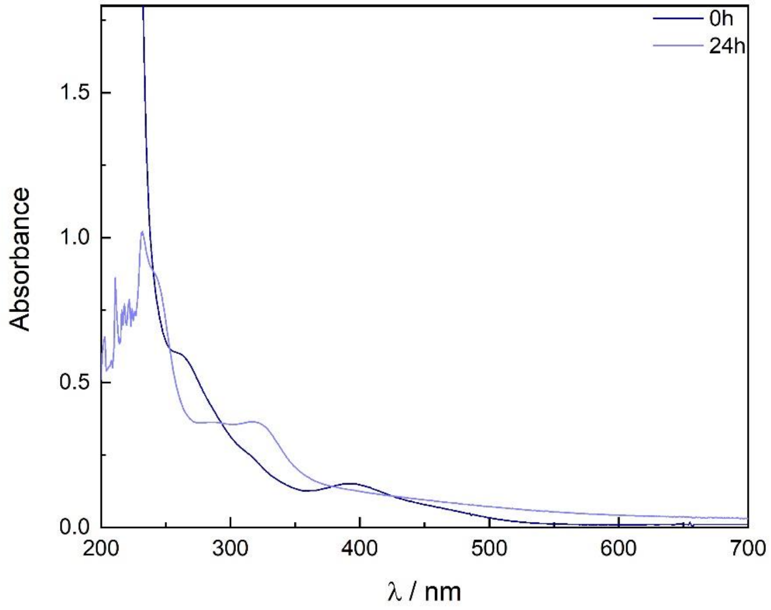

Figure S63. UV/Vis stability measurements of complex **4c** in H_2O over 24h at 25 °C.

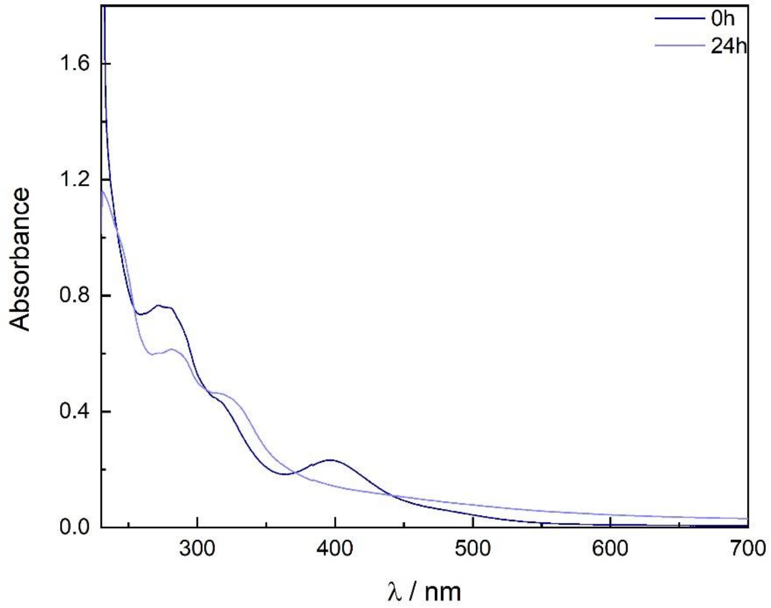

Figure S64. UV/Vis stability measurements of complex **4d** in H_2O over 24h at 25 °C.

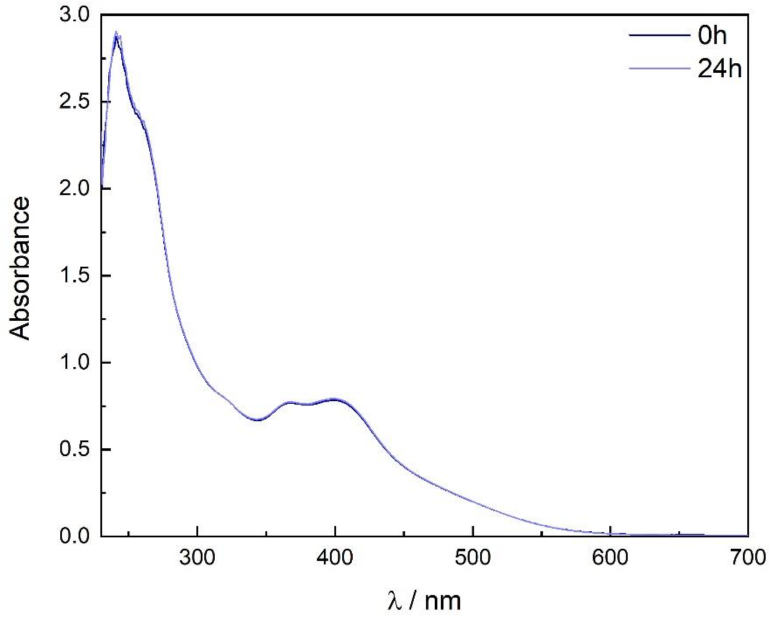

Figure S65. UV/Vis stability measurements of complex **5a** in H_2O over 24h at 25 °C.

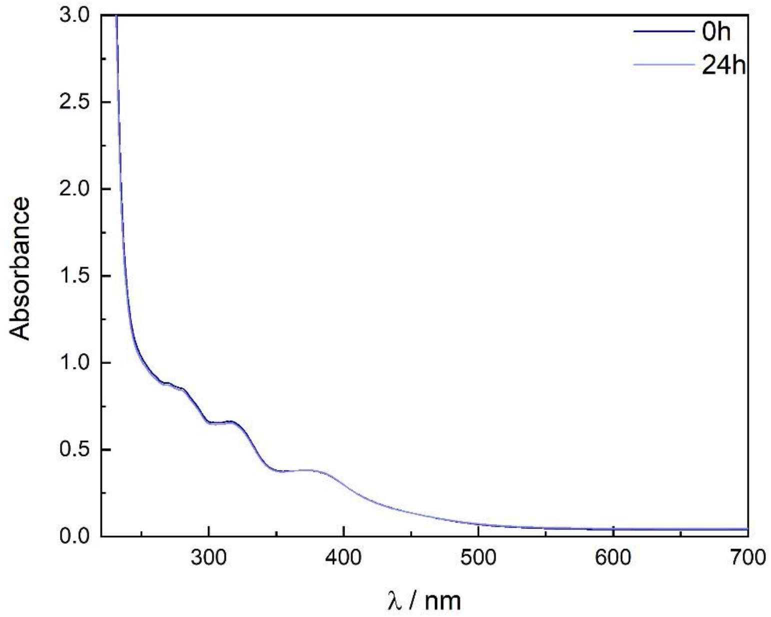

Figure S66. UV/Vis stability measurements of complex **5b** in H_2O over 24h at 25 °C.

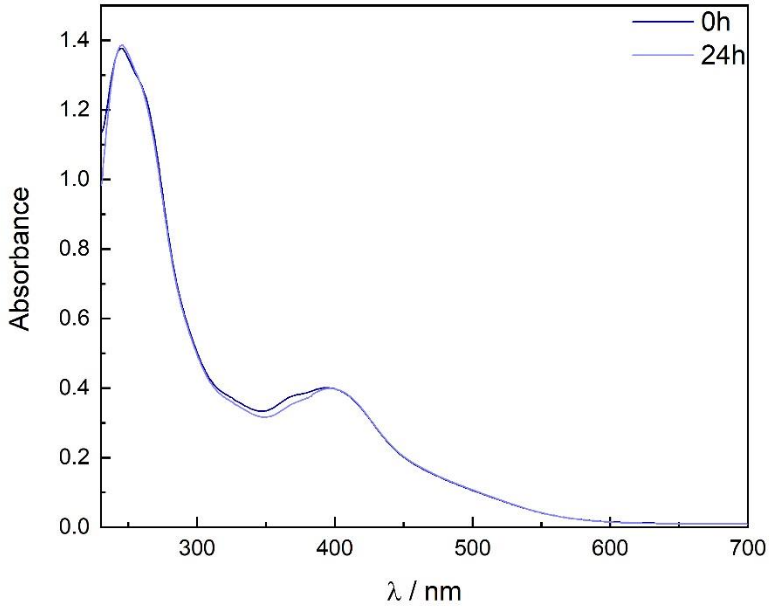

Figure S67. UV/Vis stability measurements of complex **5c** in H_2O over 24h at 25 °C.

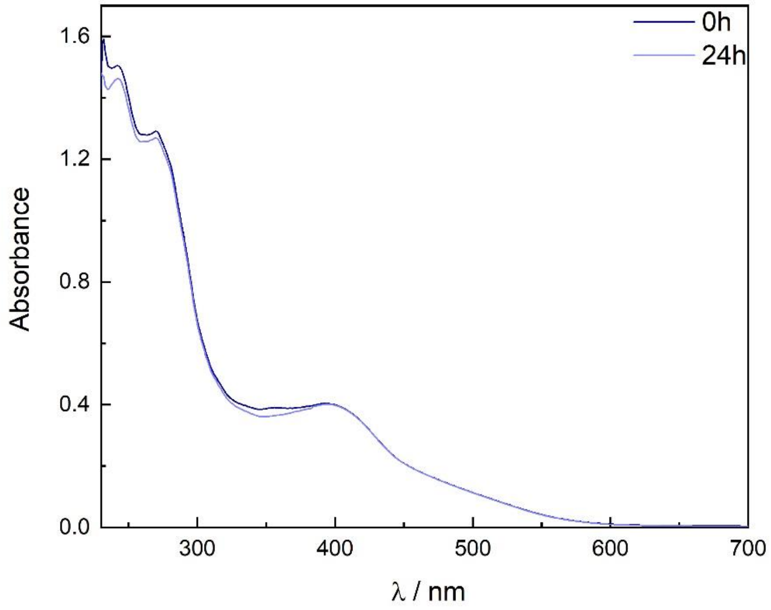

Figure S68. UV/Vis stability measurements of complex **5d** in H_2O over 24h at 25 °C.

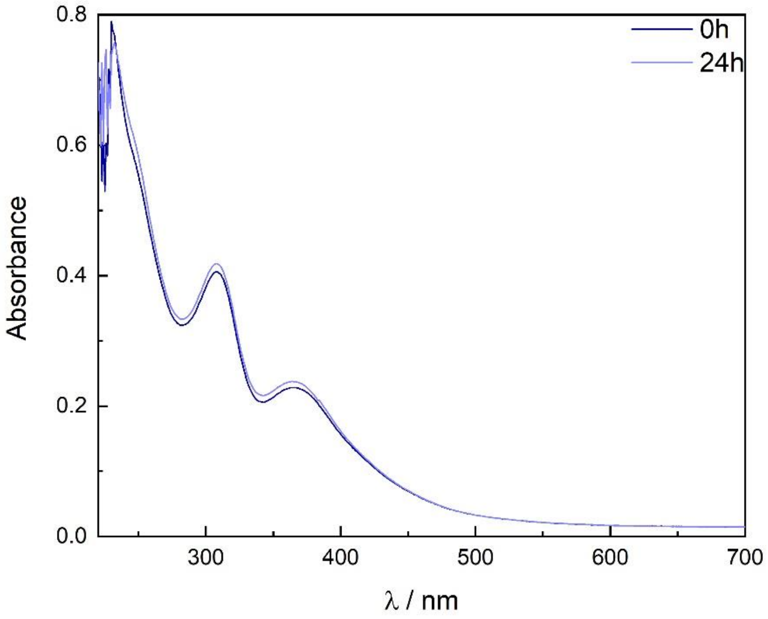

Figure S69. UV/Vis stability measurements of complex **6a** in H_2O over 24h at 25 °C.

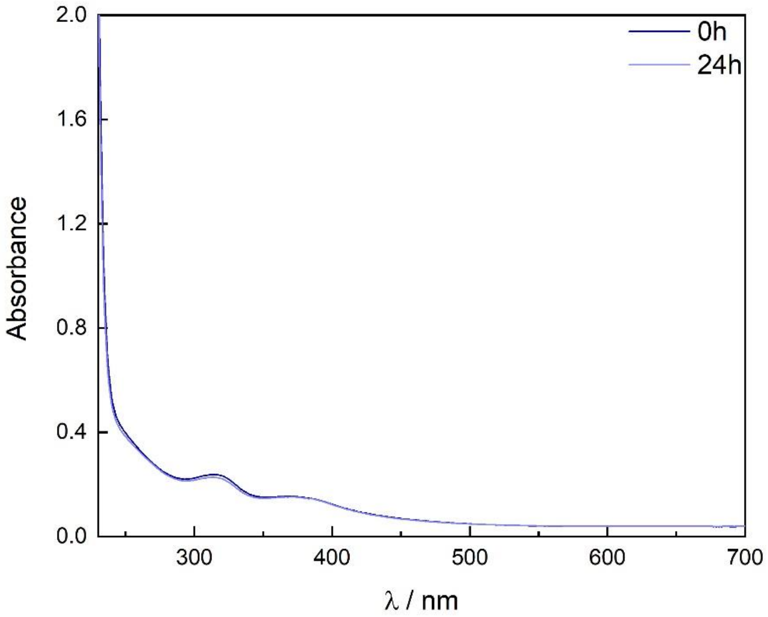

Figure S70. UV/Vis stability measurements of complex **6b** in H_2O over 24h at 25 °C.

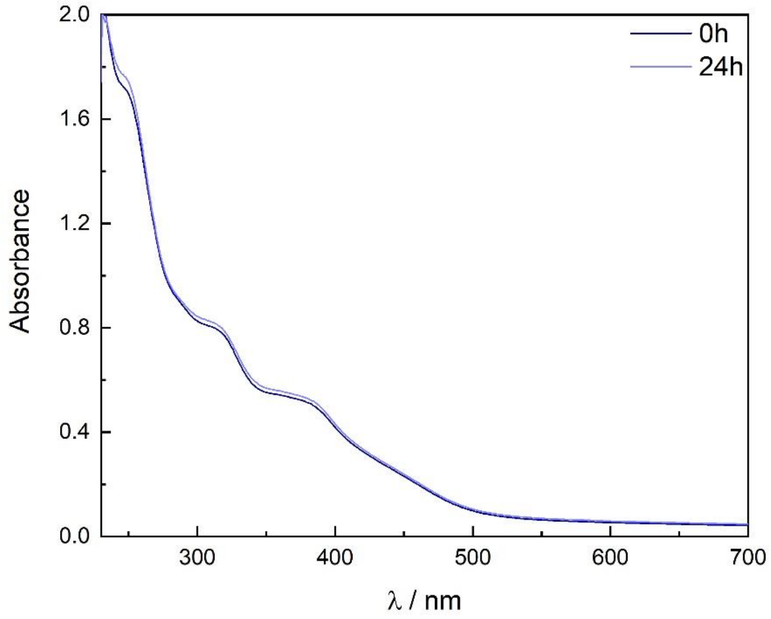

Figure S71. UV/Vis stability measurements of complex **6c** in H_2O over 24h at 25 °C.

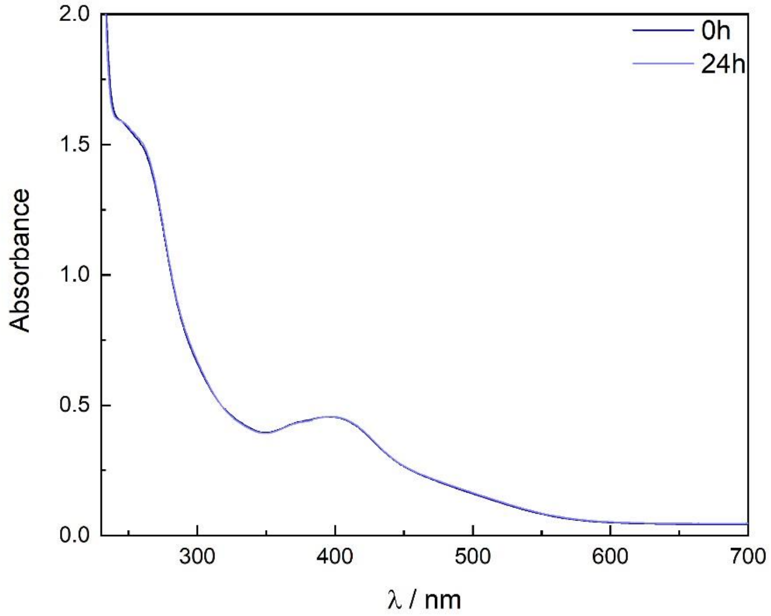

Figure S72. UV/Vis stability measurements of complex **6d** in H_2O over 24h at 25 °C.

HPLC stabilities

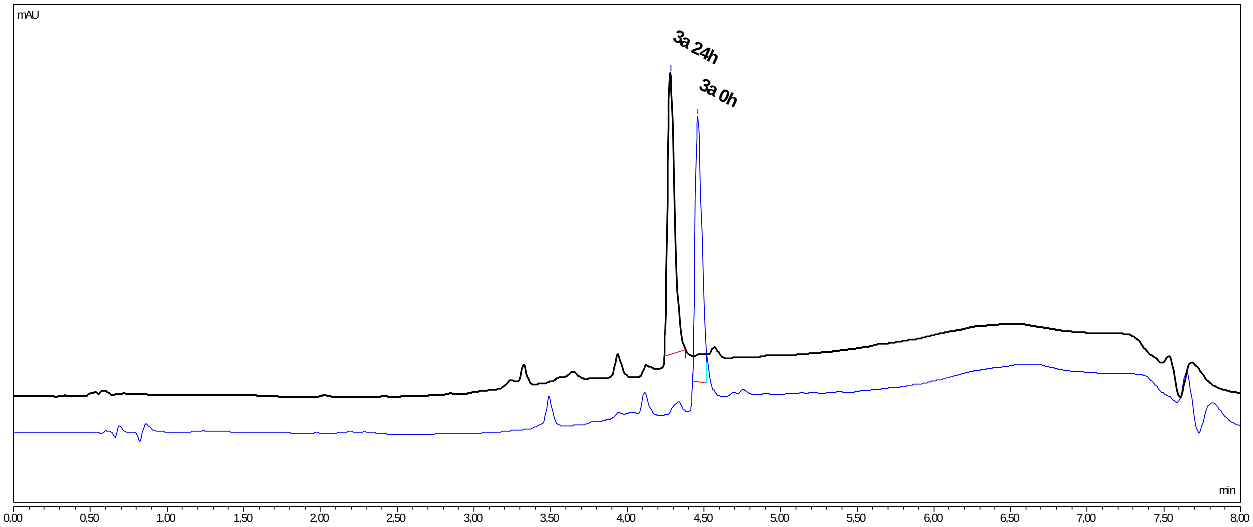

Figure S73. Stability of complex **3a** in 1% DMSO/phosphate buffer (20 mM, pH 5.8) over 24 h.

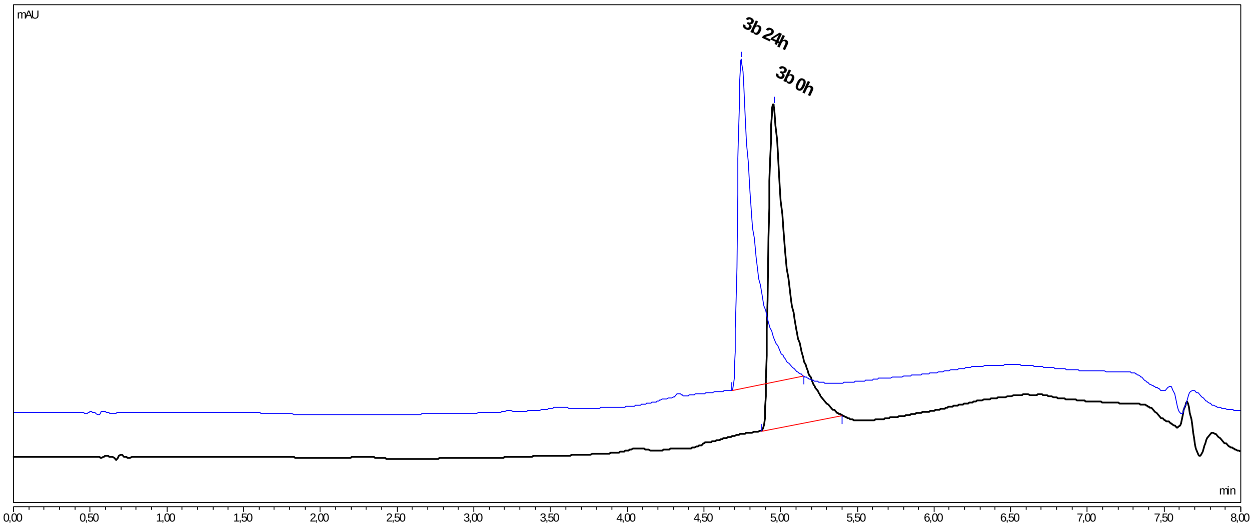

Figure S74. Stability of complex **3b** in 1% DMSO/phosphate buffer (20 mM, pH 5.8) over 24 h.

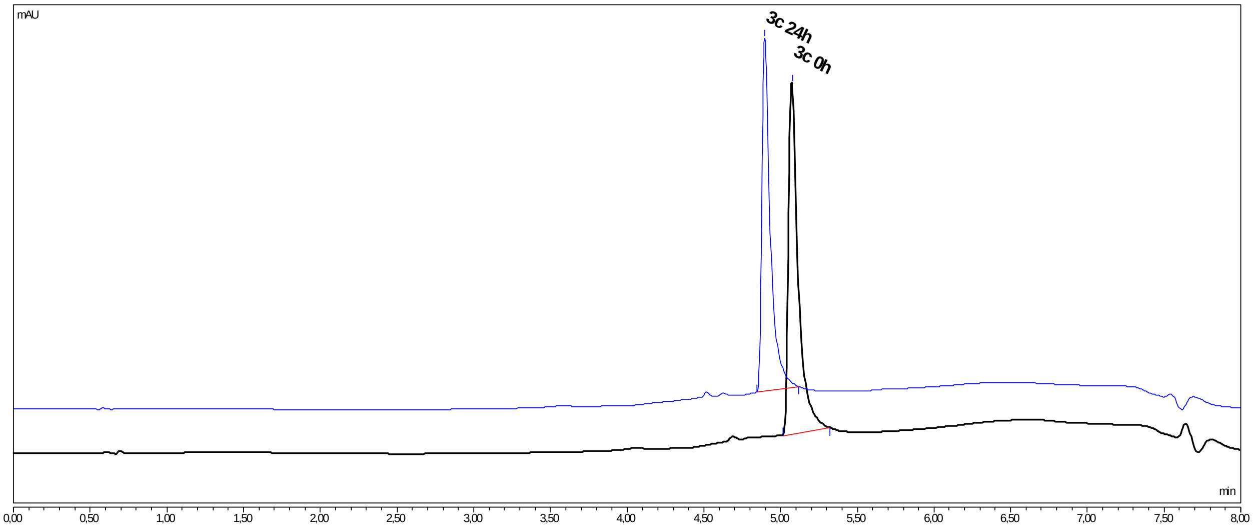

Figure S75. Stability of complex **3c** in 1% DMSO/phosphate buffer (20 mM, pH 5.8) over 24 h.

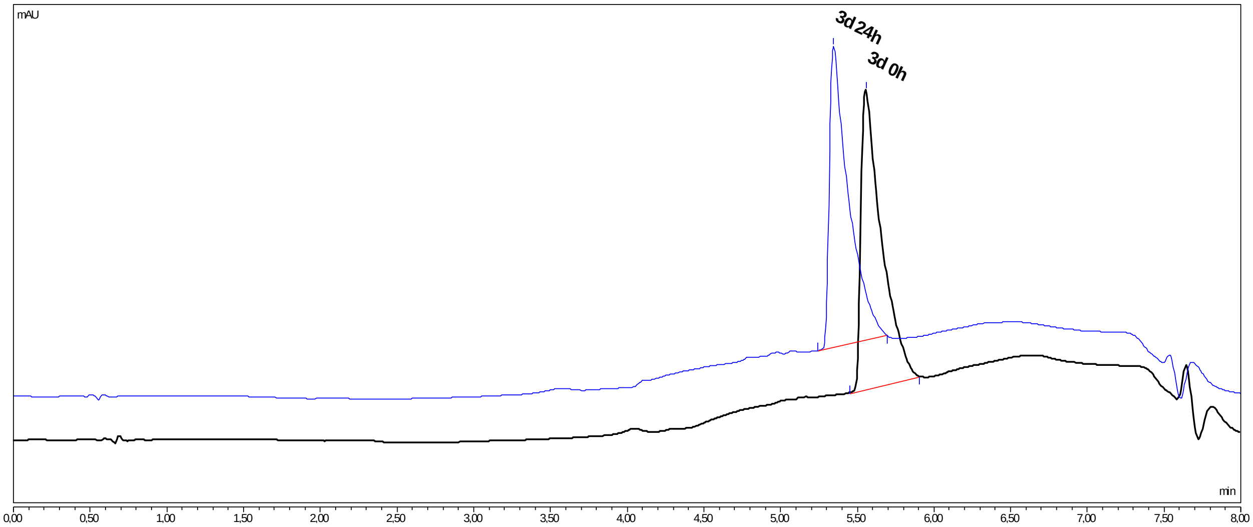

Figure S76. Stability of complex **3d** in 1% DMSO/phosphate buffer (20 mM, pH 5.8) over 24 h.

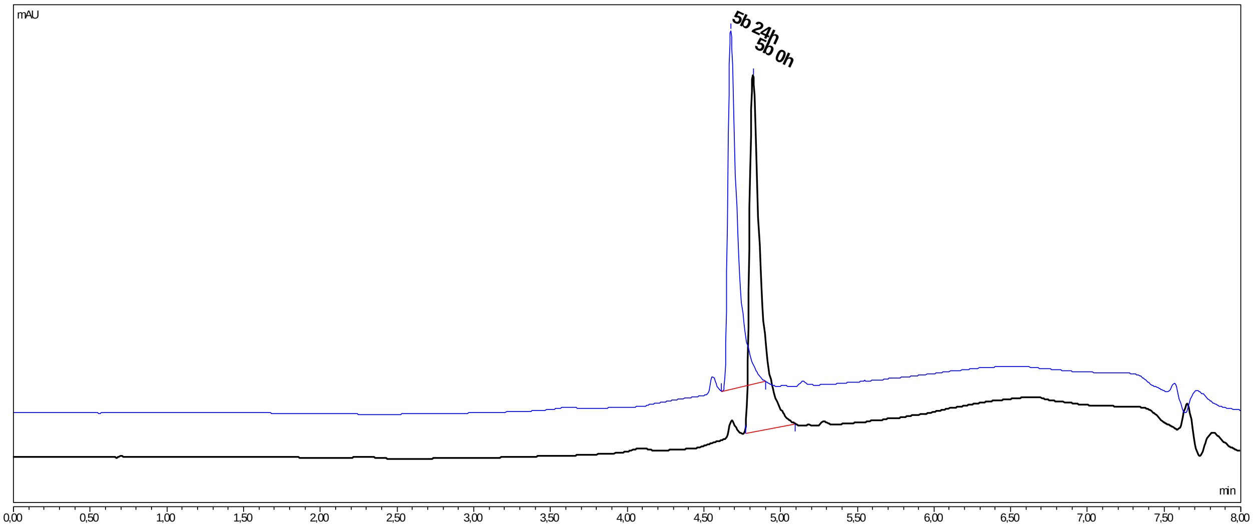

Figure S 77. Stability of complex **5b** in 1% DMSO/phosphate buffer (20 mM, pH 5.8) over 24 h.

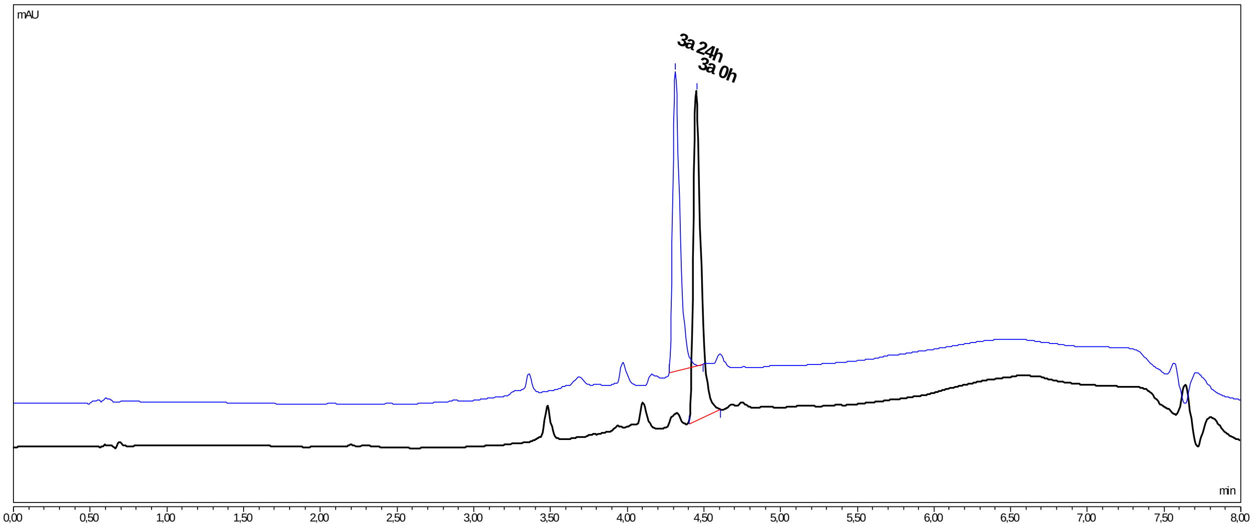

Figure S 78. Stability of complex **3a** in 1% DMSO/phosphate buffer (20 mM, pH 6.2) over 24 h.

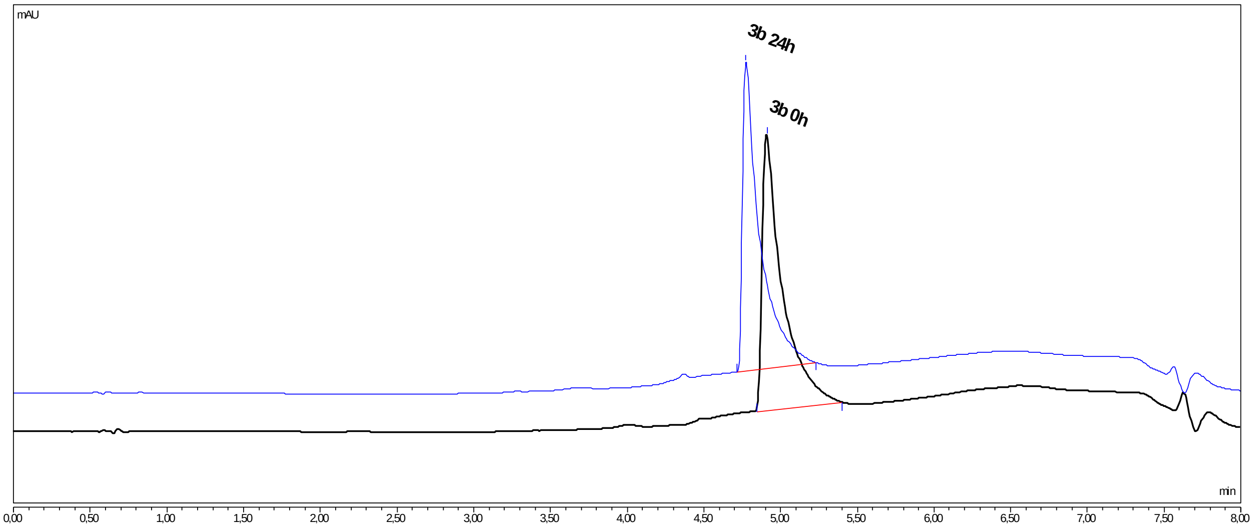

Figure S79. Stability of complex **3b** in 1% DMSO/phosphate buffer (20 mM, pH 6.2) over 24 h.

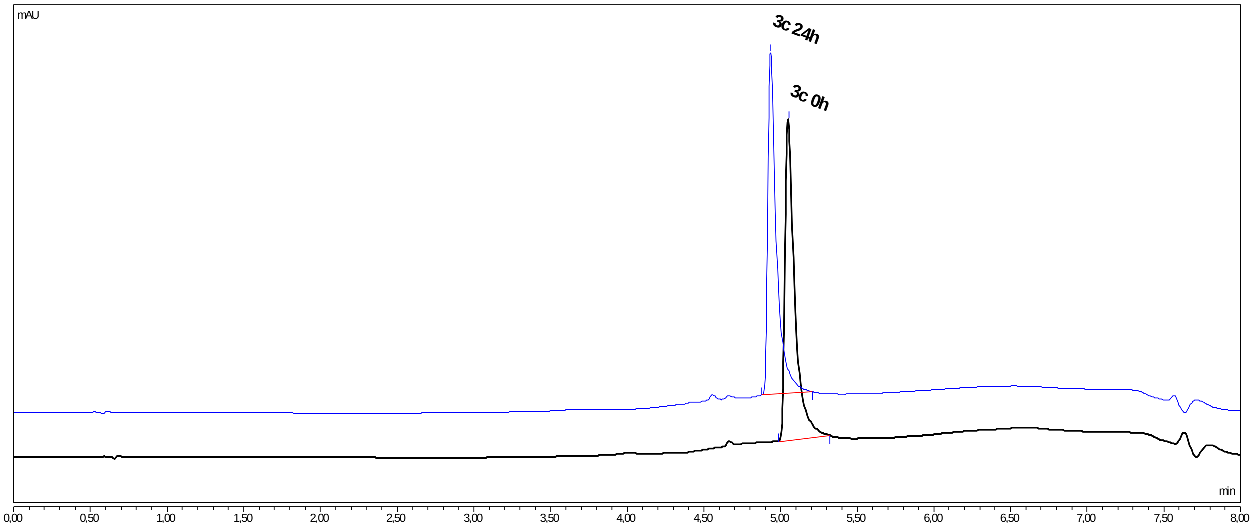

Figure S80. Stability of complex **3c** in 1% DMSO/phosphate buffer (20 mM, pH 6.2) over 24 h.

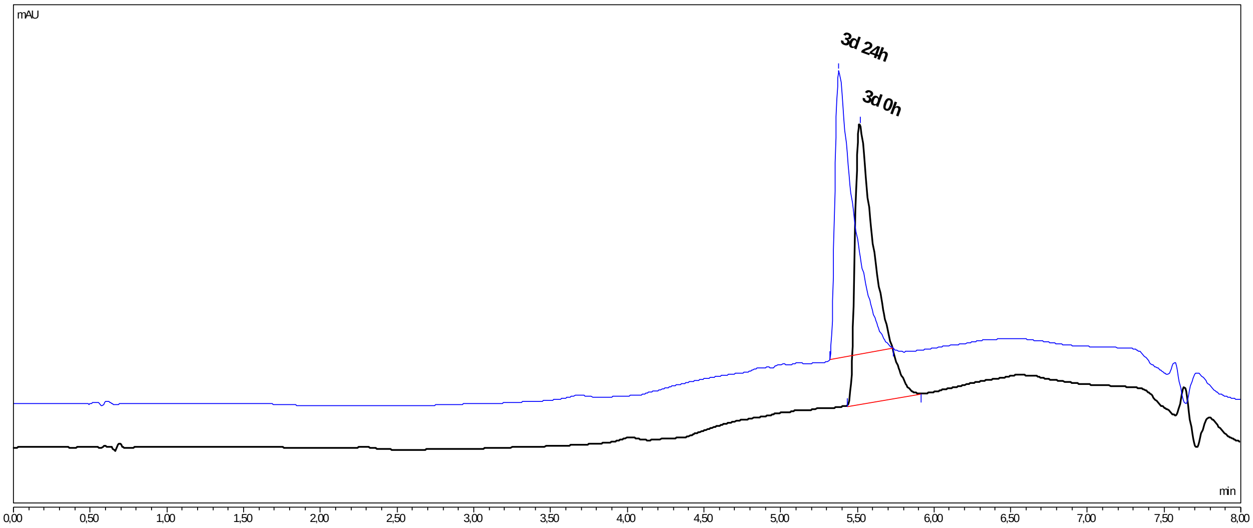

Figure S81. Stability of complex **3d** in 1% DMSO/phosphate buffer (20 mM, pH 6.2) over 24 h.

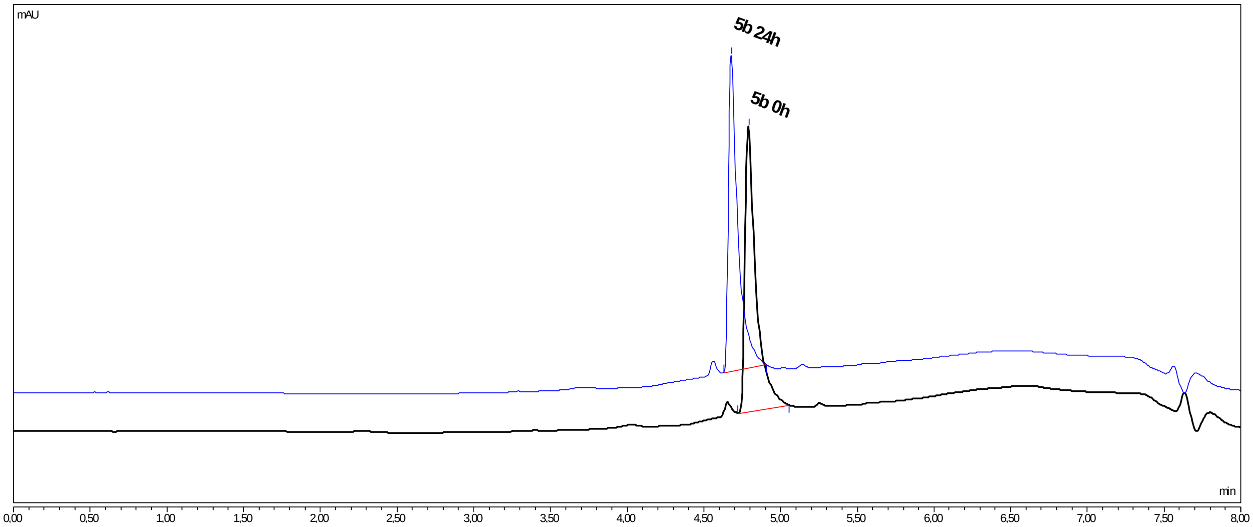

Figure S82. Stability of complex **5b** in 1% DMSO/phosphate buffer (20 mM, pH 6.2) over 24 h.

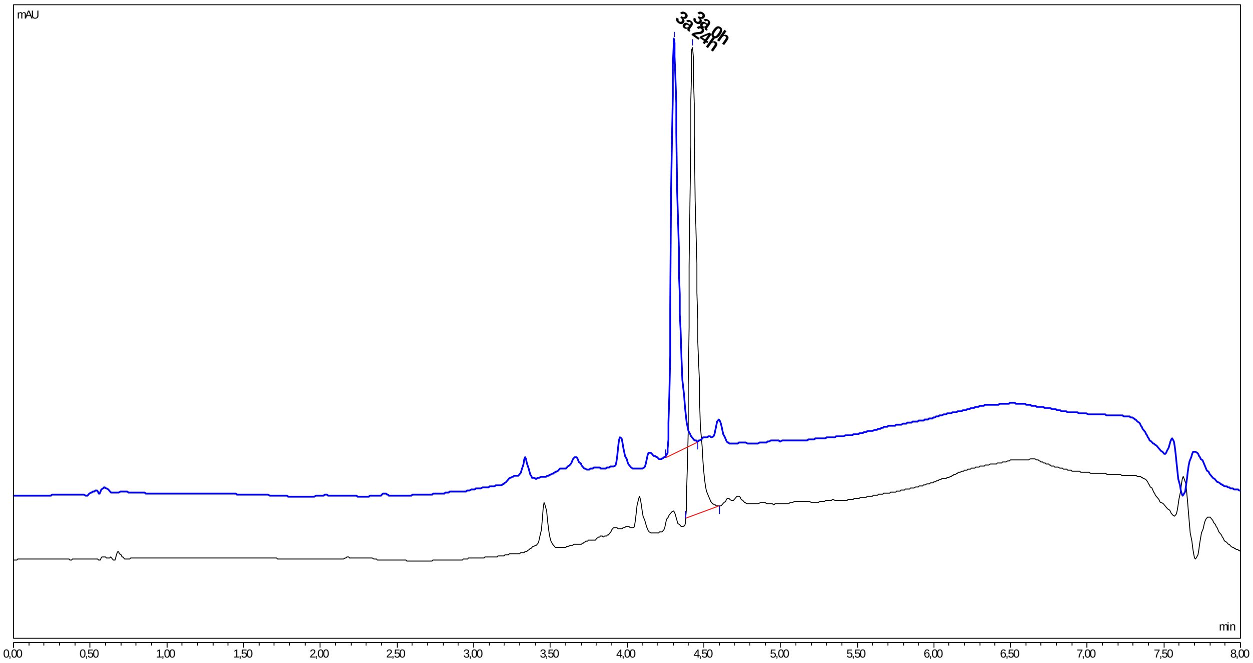

Figure S83. Stability of complex **3a** in 1% DMSO/phosphate buffer (20 mM, pH 6.7) over 24 h.

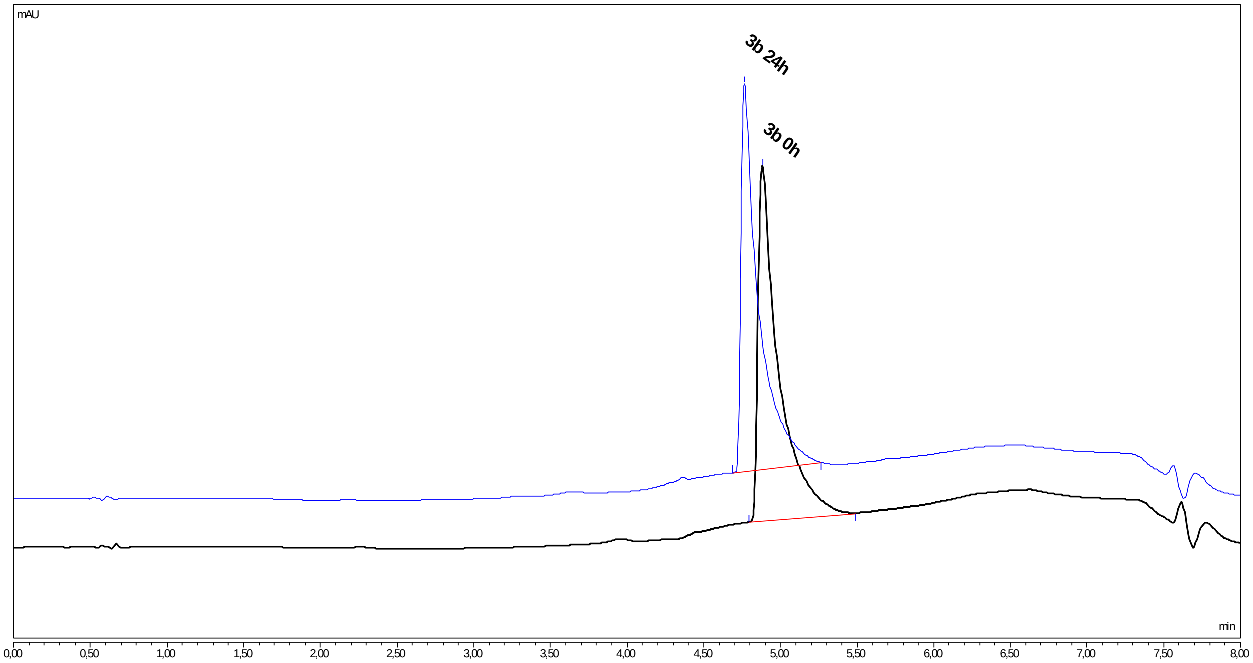

Figure S84. Stability of complex **3b** in 1% DMSO/phosphate buffer (20 mM, pH 6.7) over 24 h.

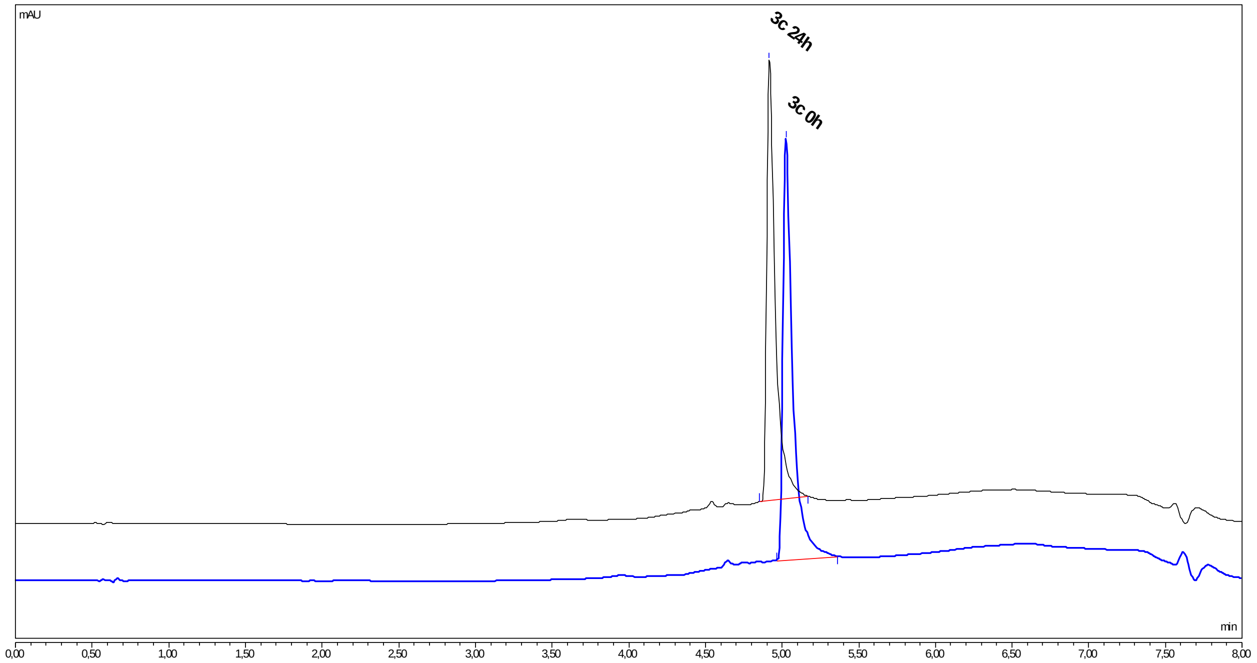

Figure S85. Stability of complex **3c** in 1% DMSO/phosphate buffer (20 mM, pH 6.7) over 24 h.

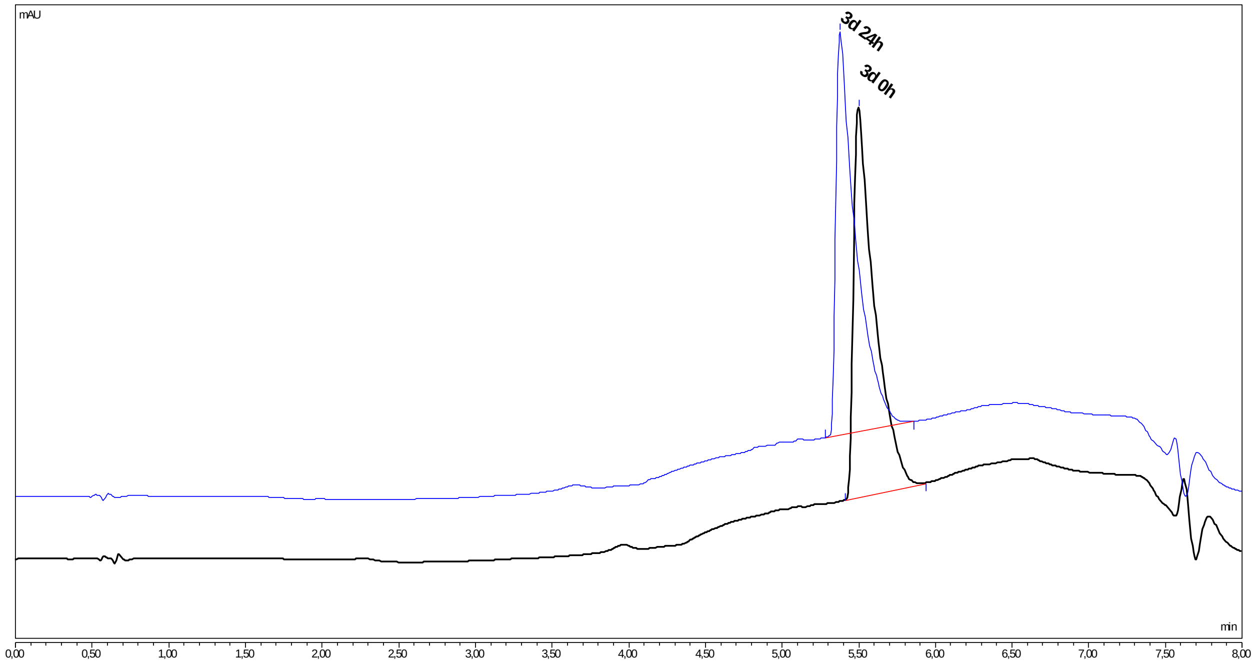

Figure S86. Stability of complex **3d** in 1% DMSO/phosphate buffer (20 mM, pH 6.7) over 24 h.

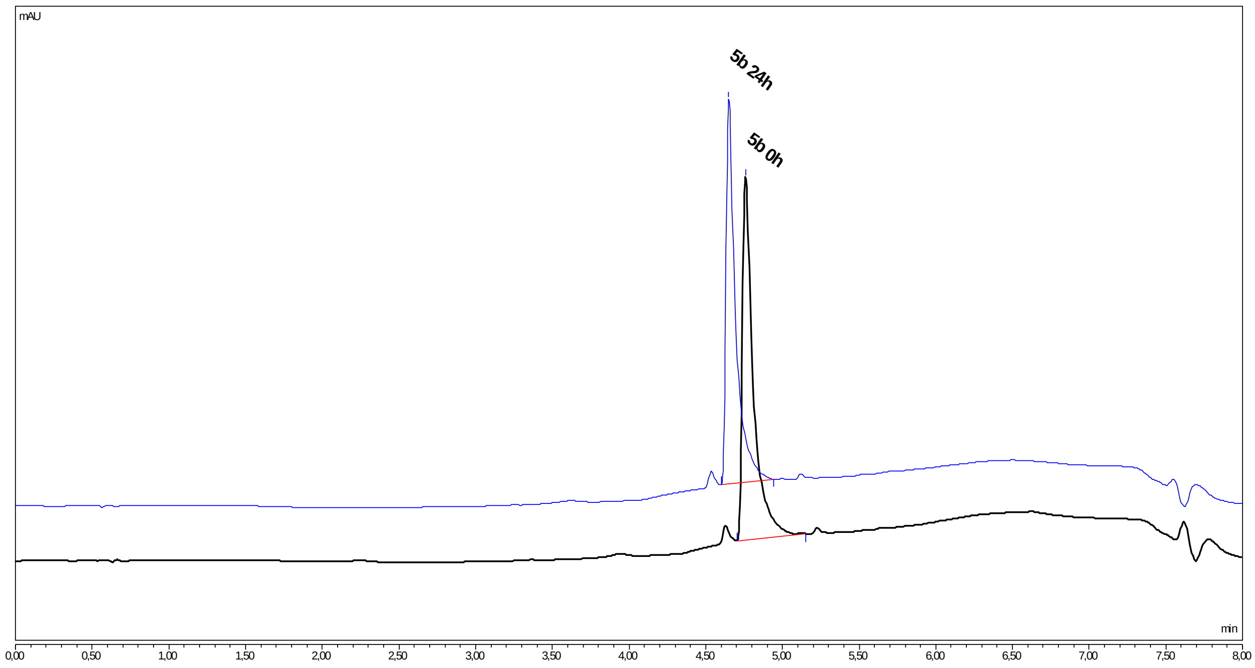

Figure S87. Stability of complex **5b** in 1% DMSO/phosphate buffer (20 mM, pH 6.7) over 24 h.

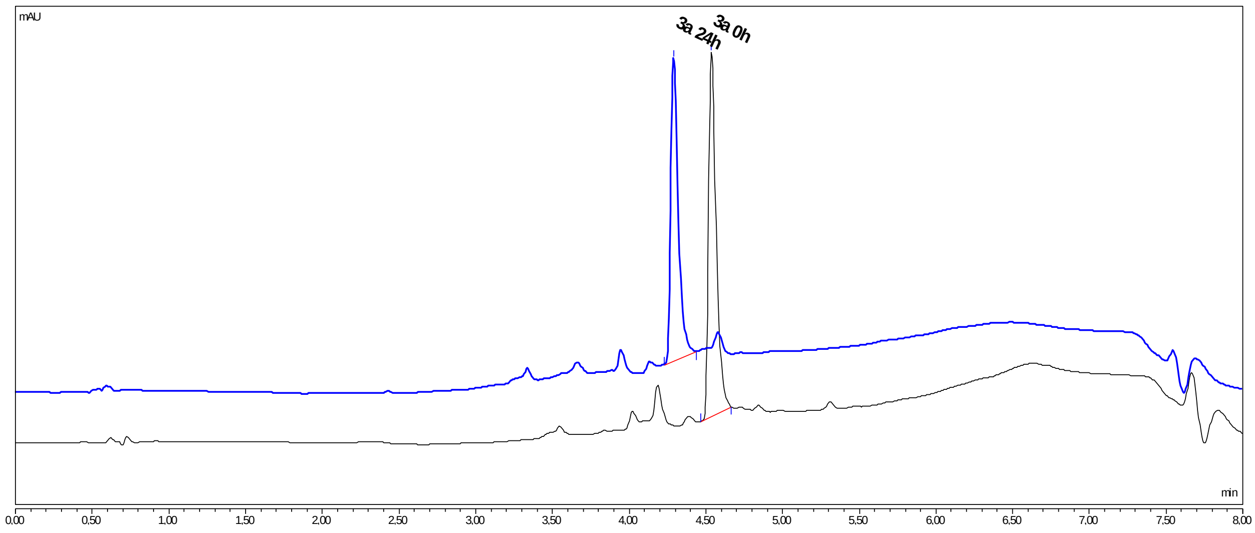

Figure S88. Stability of complex **3a** in 1% DMSO/phosphate buffer (20 mM, pH 7.2) over 24 h.

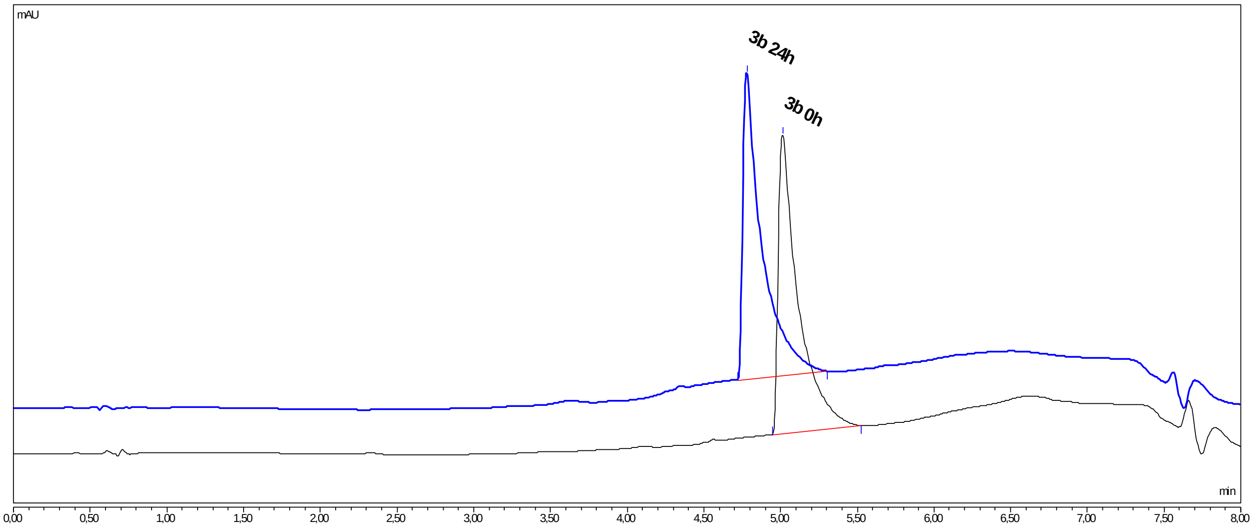

Figure S89. Stability of complex **3b** in 1% DMSO/phosphate buffer (20 mM, pH 7.2) over 24 h.

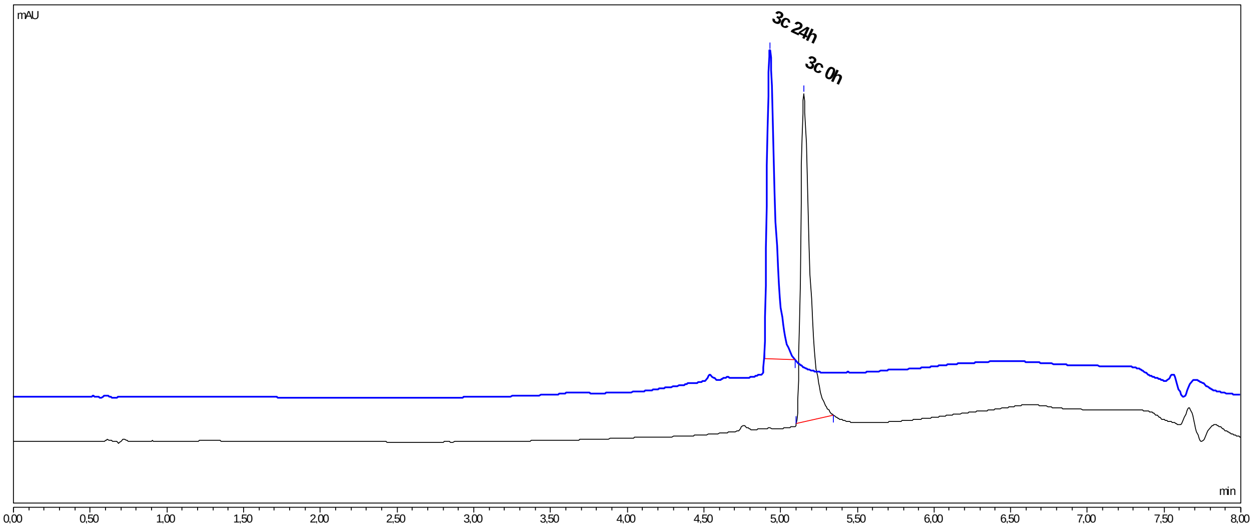

Figure S90. Stability of complex **3c** in 1% DMSO/phosphate buffer (20 mM, pH 7.2) over 24 h.

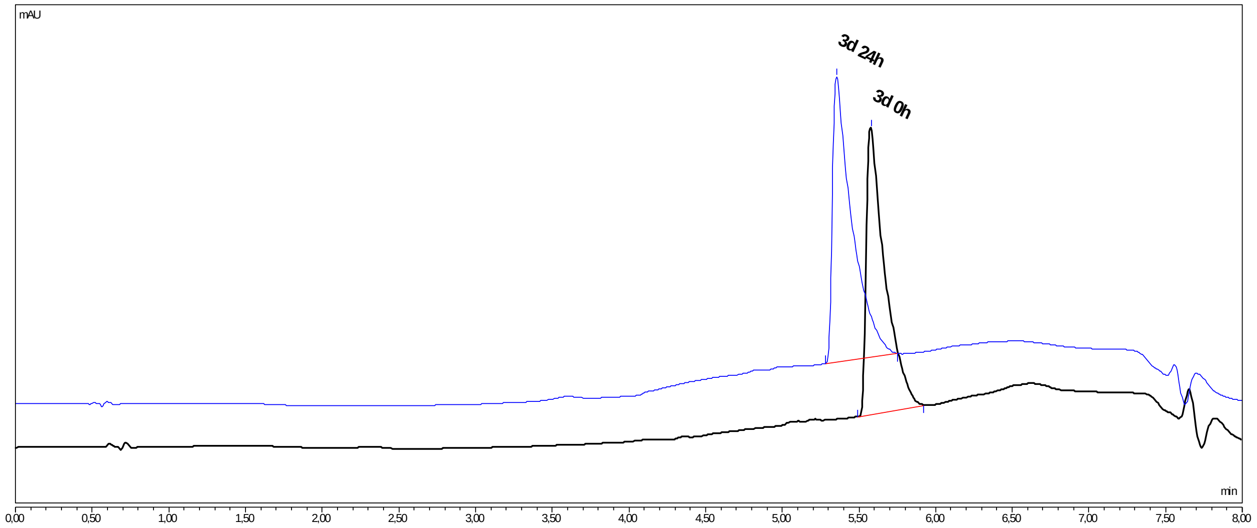

Figure S91. Stability of complex **3d** in 1% DMSO/phosphate buffer (20 mM, pH 7.2) over 24 h.

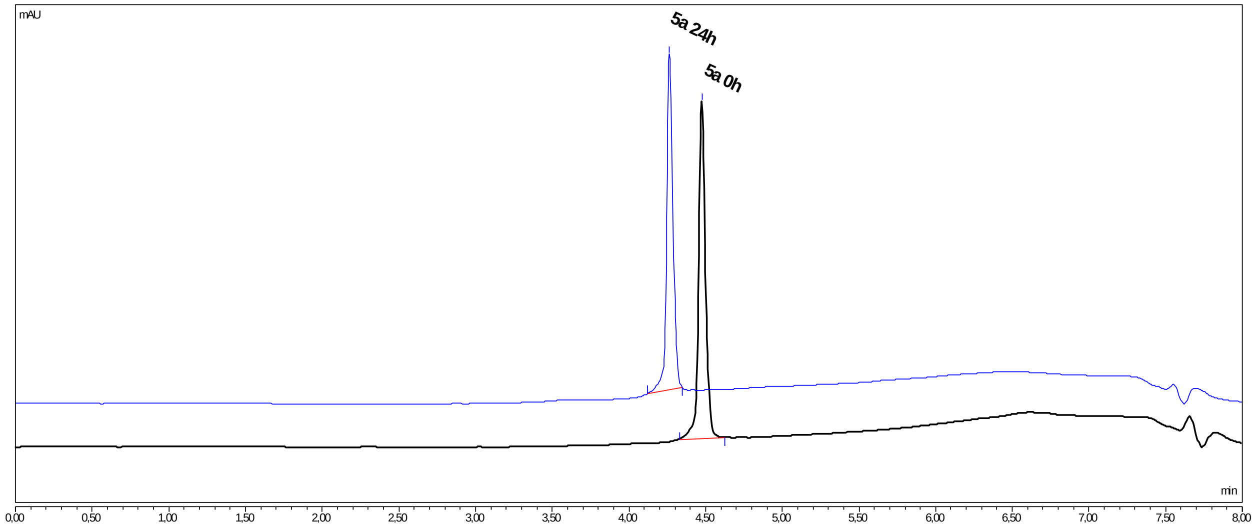

Figure S92. Stability of complex **5a** in 1% DMSO/phosphate buffer (20 mM, pH 7.2) over 24 h.

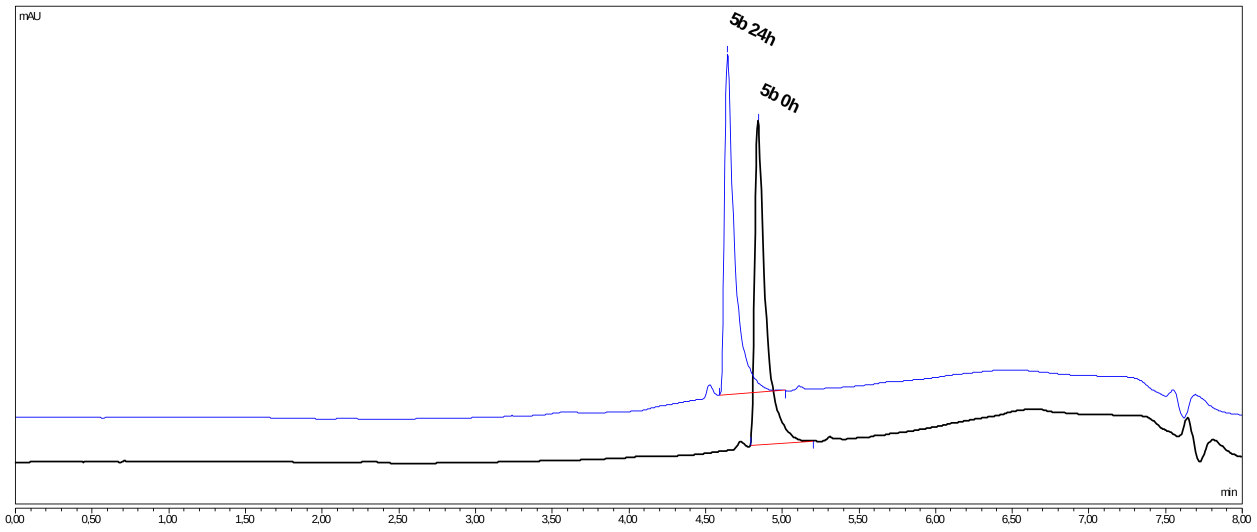

Figure S93. Stability of complex **5b** in 1% DMSO/phosphate buffer (20 mM, pH 7.2) over 24 h.

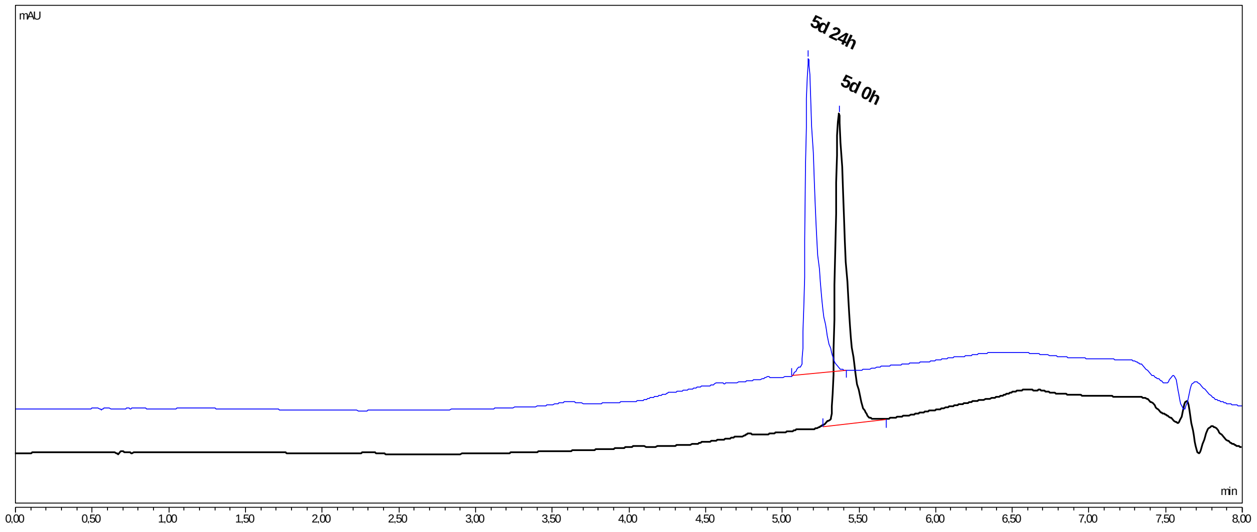

Figure S94. Stability of complex **5d** in 1% DMSO/phosphate buffer (20 mM, pH 7.2) over 24 h.

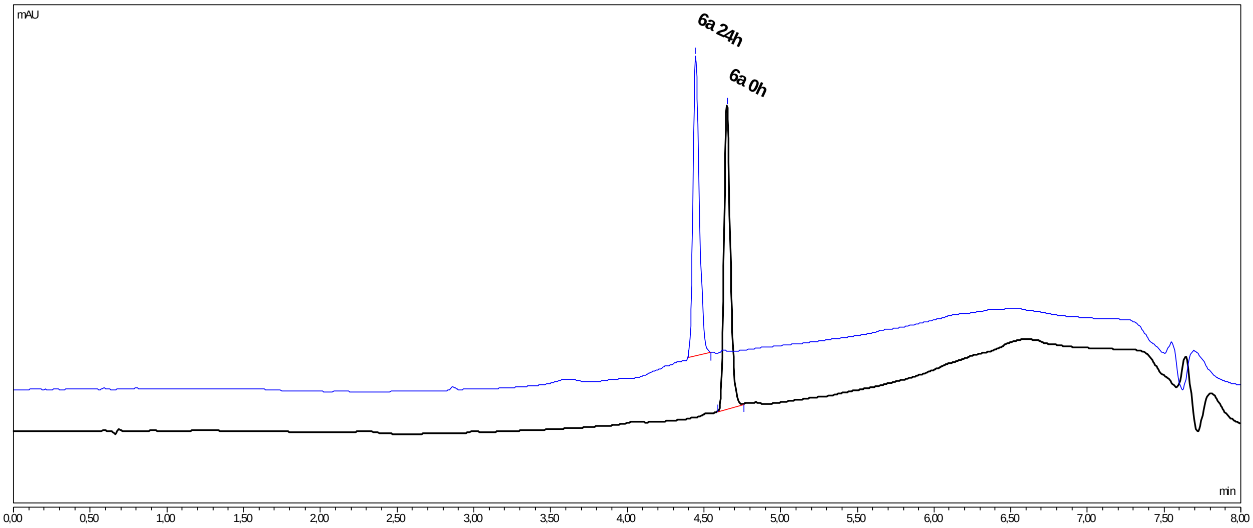

Figure S95. Stability of complex **6a** in 1% DMSO/phosphate buffer (20 mM, pH 7.2) over 24 h.

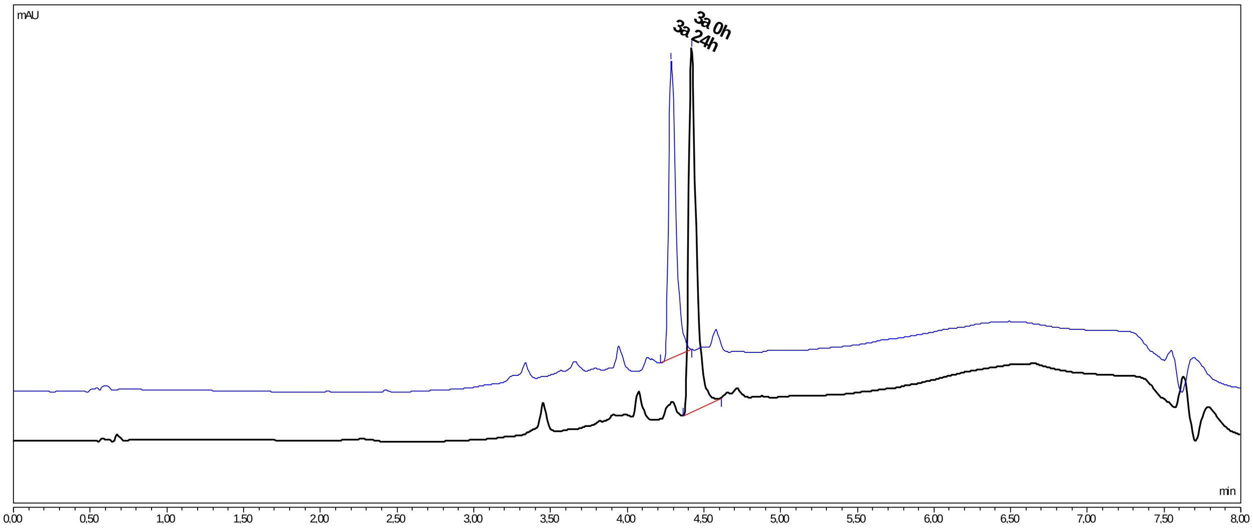

Figure S96. Stability of complex **3a** in 1% DMSO/phosphate buffer (20 mM, pH 7.9) over 24 h.

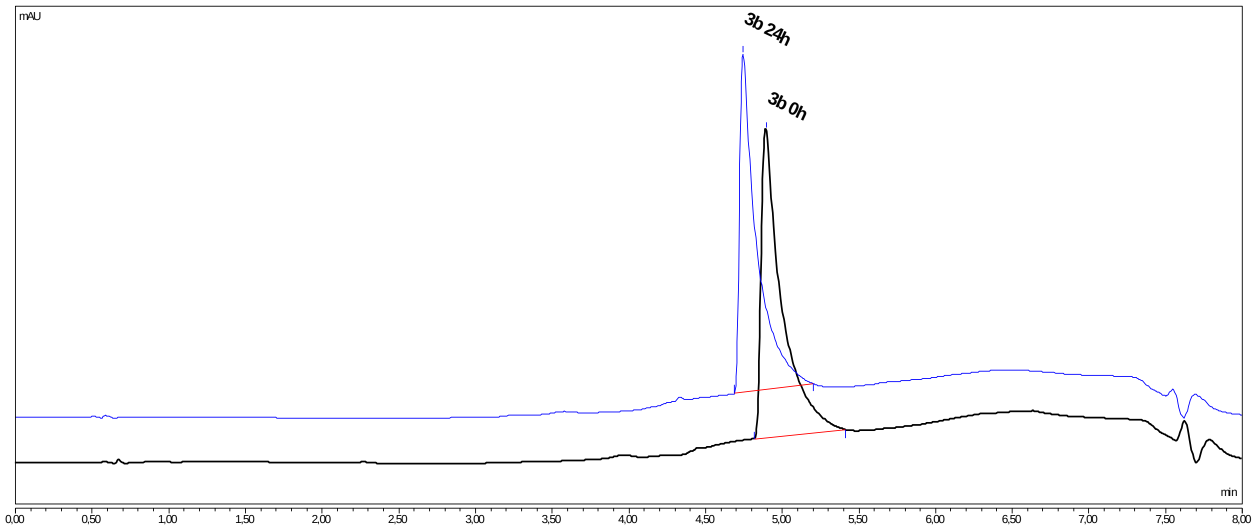

Figure S97. Stability of complex **3b** in 1% DMSO/phosphate buffer (20 mM, pH 7.9) over 24 h.

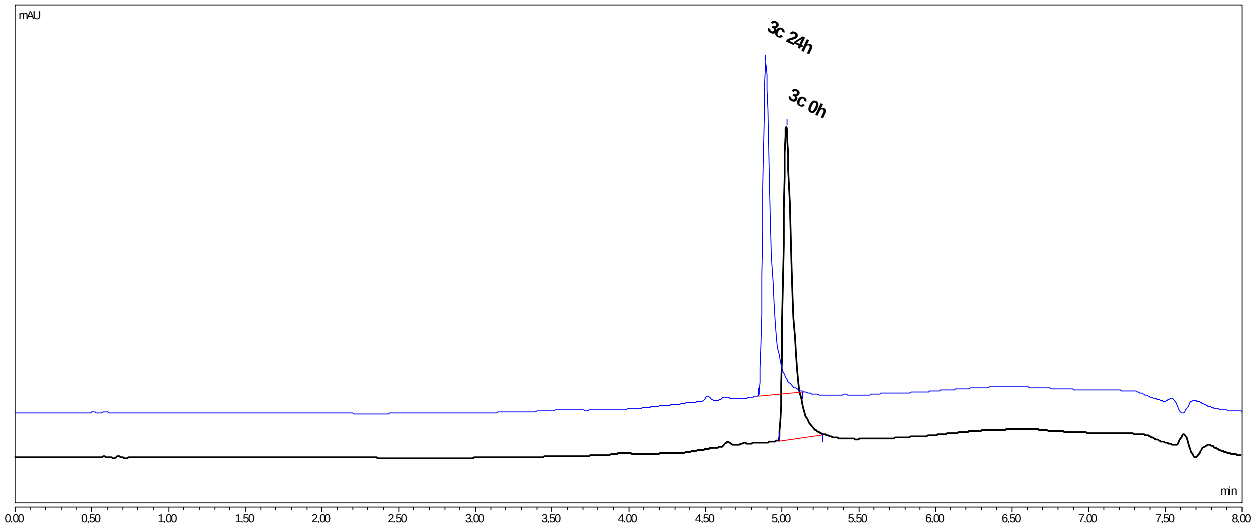

Figure S98. Stability of complex **3c** in 1% DMSO/phosphate buffer (20 mM, pH 7.9) over 24 h.

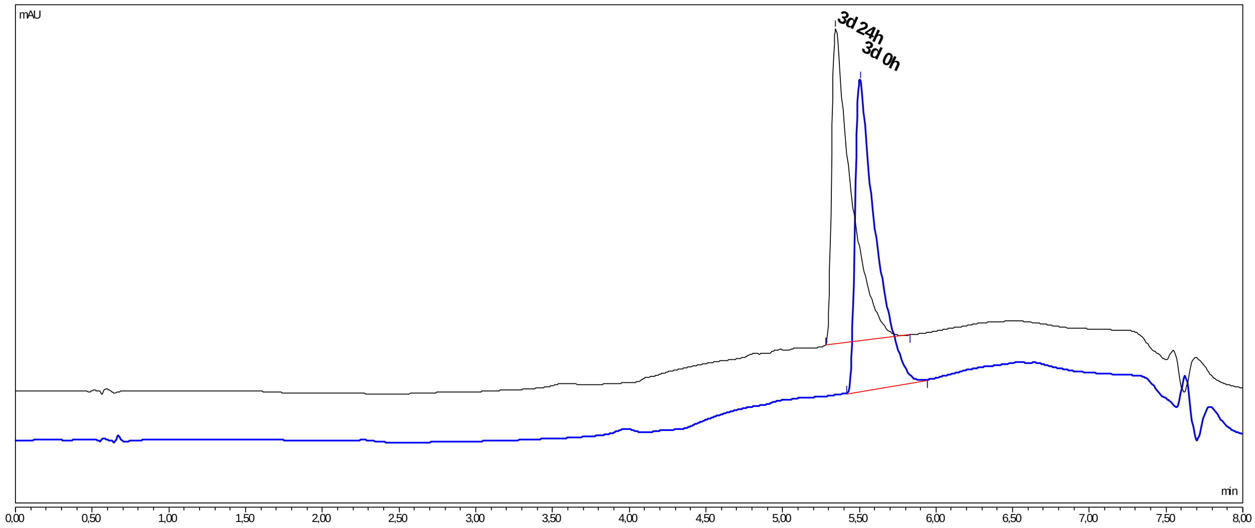

Figure S99. Stability of complex **3d** in 1% DMSO/phosphate buffer (20 mM, pH 7.9) over 24 h.

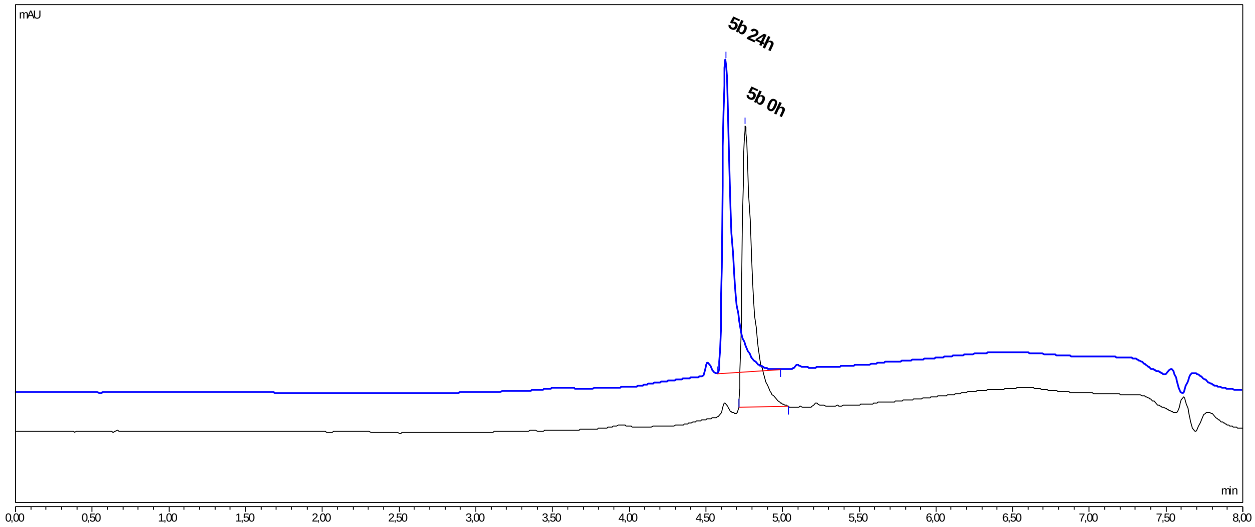

Figure S100. Stability of complex **5b** in 1% DMSO/phosphate buffer (20 mM, pH 7.9) over 24 h.

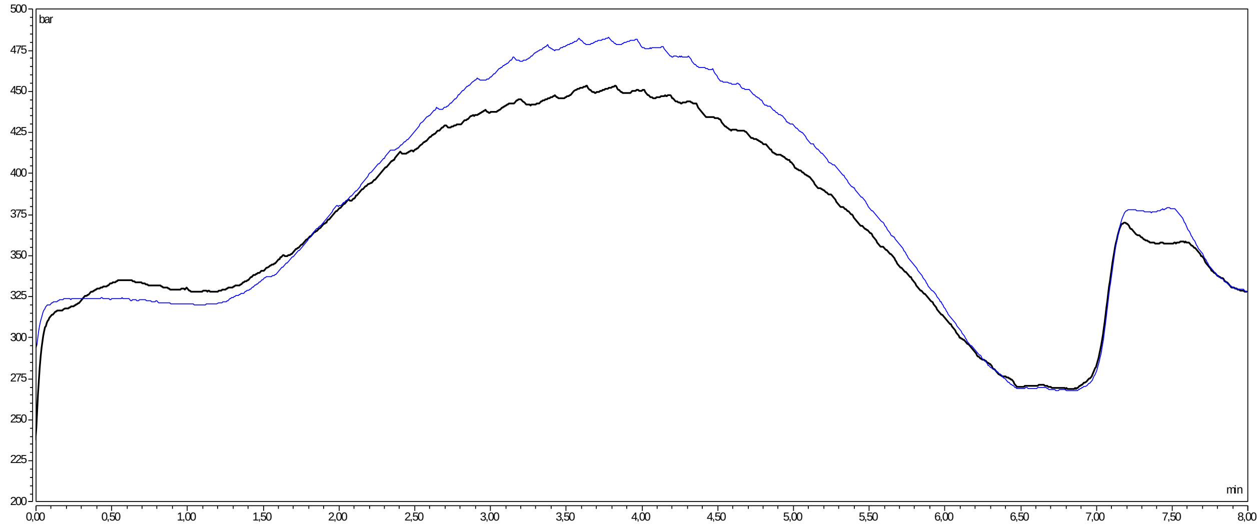

Figure S101. Overlay of pump pressure fluctuations of UPLC runs at 0 h and 24 h.

HPLC incubation

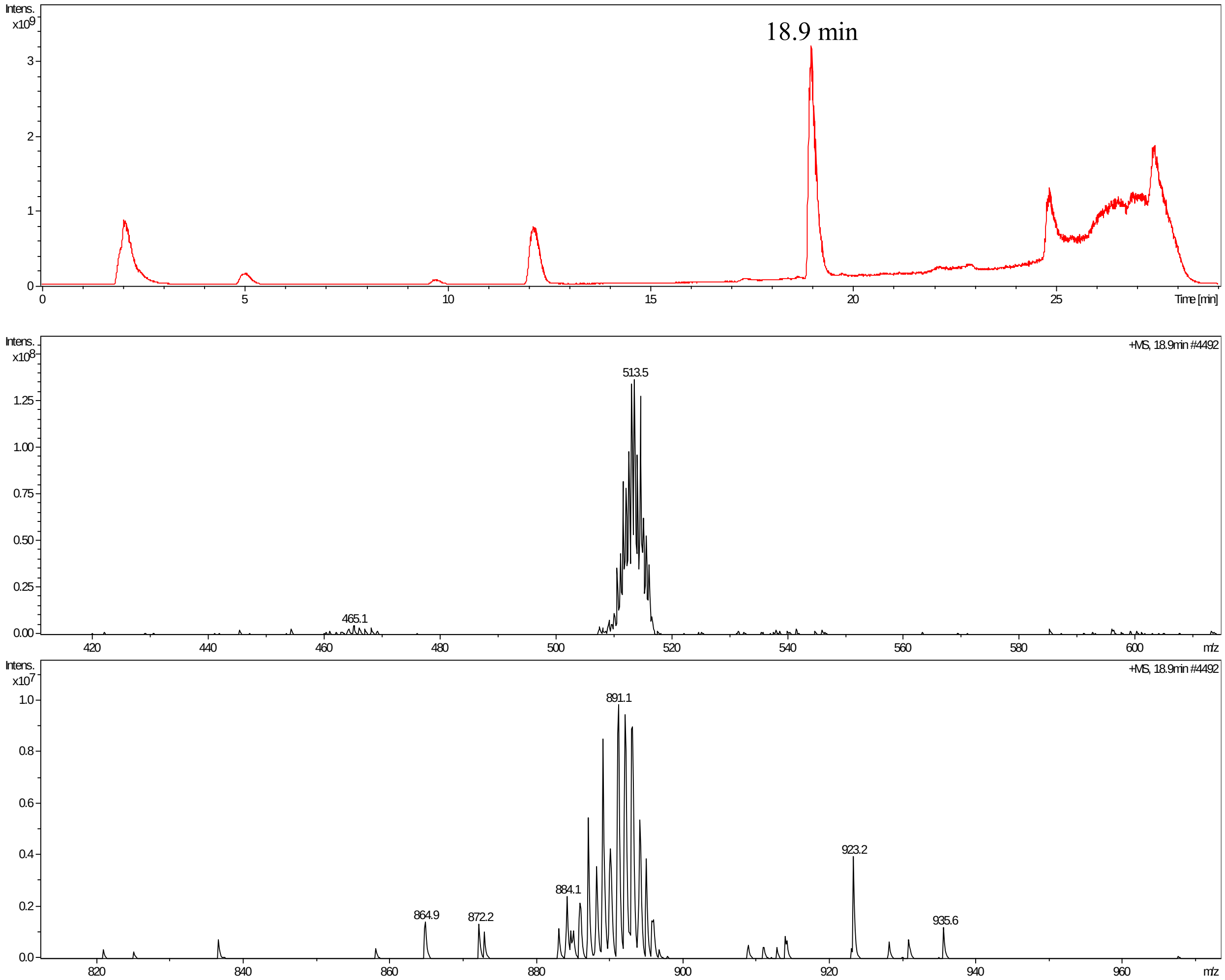

Figure S102. HPLC run of Ru-Bz complex **3c** (20 µM in 500 µM phosphate buffer, pH 7.2) after 0h (above); extracted MS compound spectrum at 18.9 min showing *m/z* according to $[((p\text{-cym})Ru)_2L(N\text{-Ac-Cys})_2]^{2+}$ (*m/z* 513.5, m_{ex} = 513.7) (middle); extracted MS compound spectrum at 18.9 min showing *m/z* according to $[((p\text{-cym})Ru)_2L(N\text{-Ac-Met})]^{+}$ (*m/z* 891.1, m_{ex} = 892.1) (below).

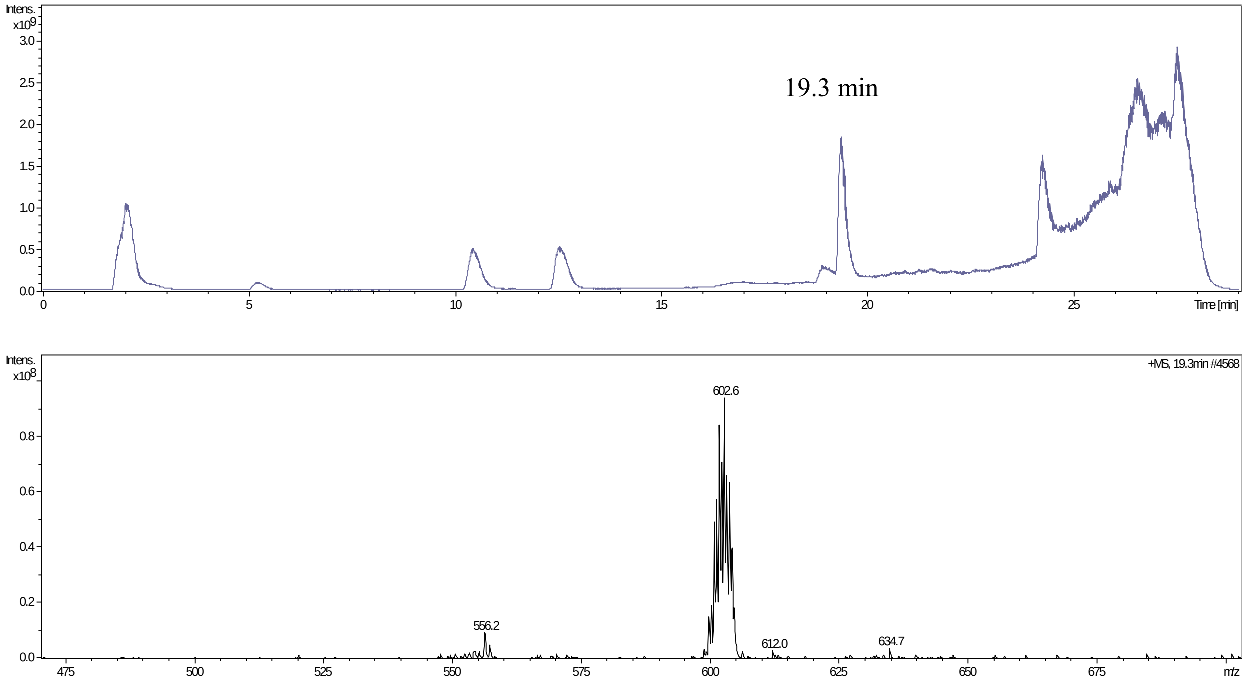

Figure S103. HPLC run of Os-Bz complex **4c** (20 µM in 500 µM phosphate buffer, pH 7.2) after 0h (above); extracted MS compound spectrum at 19.3 min showing *m/z* according to [((*p*-cym)Os)$_2$L(*N*-Ac-Cys)$_2$]$^+$ (*m/z* 601.6, m$_{ex}$ = 601.8) (below).

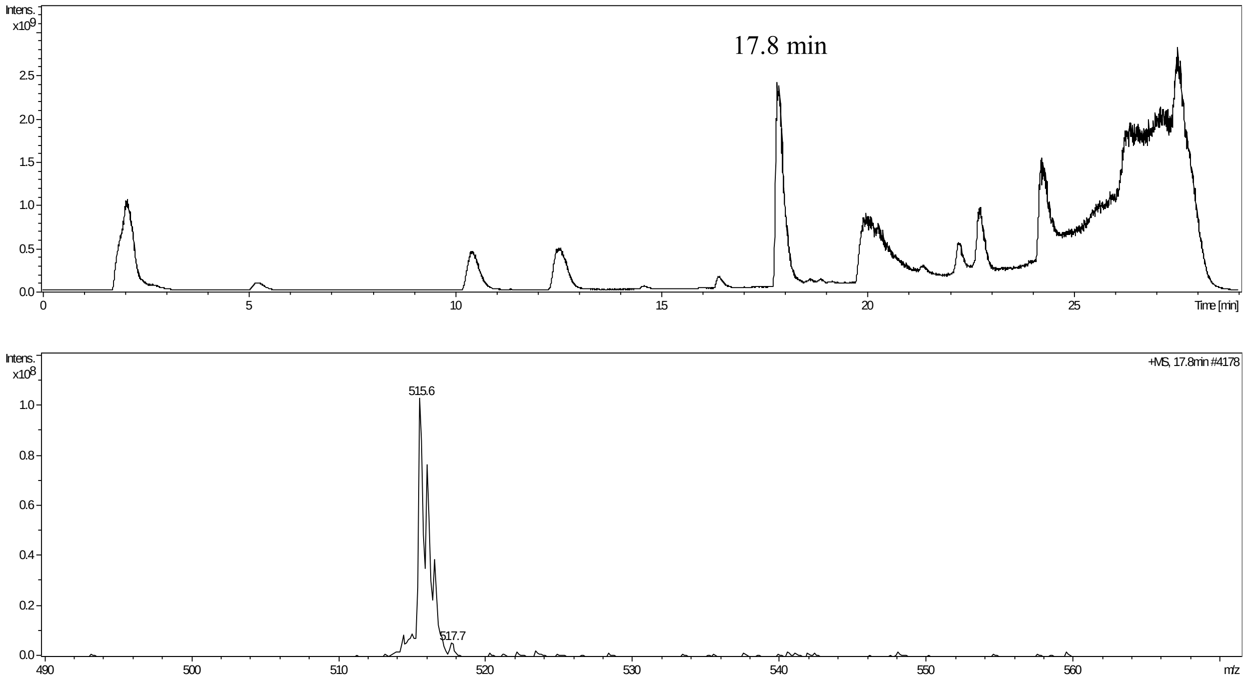

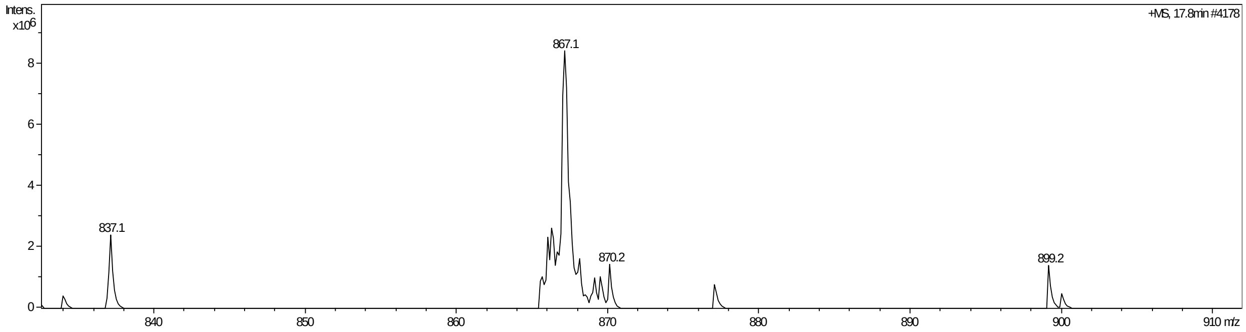

Figure S104. HPLC run of Rh-Bz complex **5c** (20 µM in 500 µM phosphate buffer, pH 7.2) after 0h (above); extracted MS compound spectrum at 17.8 min showing *m/z* according to $[((Cp^*)Rh)_2L(N\text{-Ac-Cys})_2]^{2+}$ (*m/z* 515.6, m_{ex} = 514.9) (middle); extracted MS compound spectrum at 17.8 min showing *m/z* according to $[((Cp^*)Rh)_2L(N\text{-Ac-Cys})]^{+}$ (*m/z* 867.1, m_{ex} = 869.8) (below).

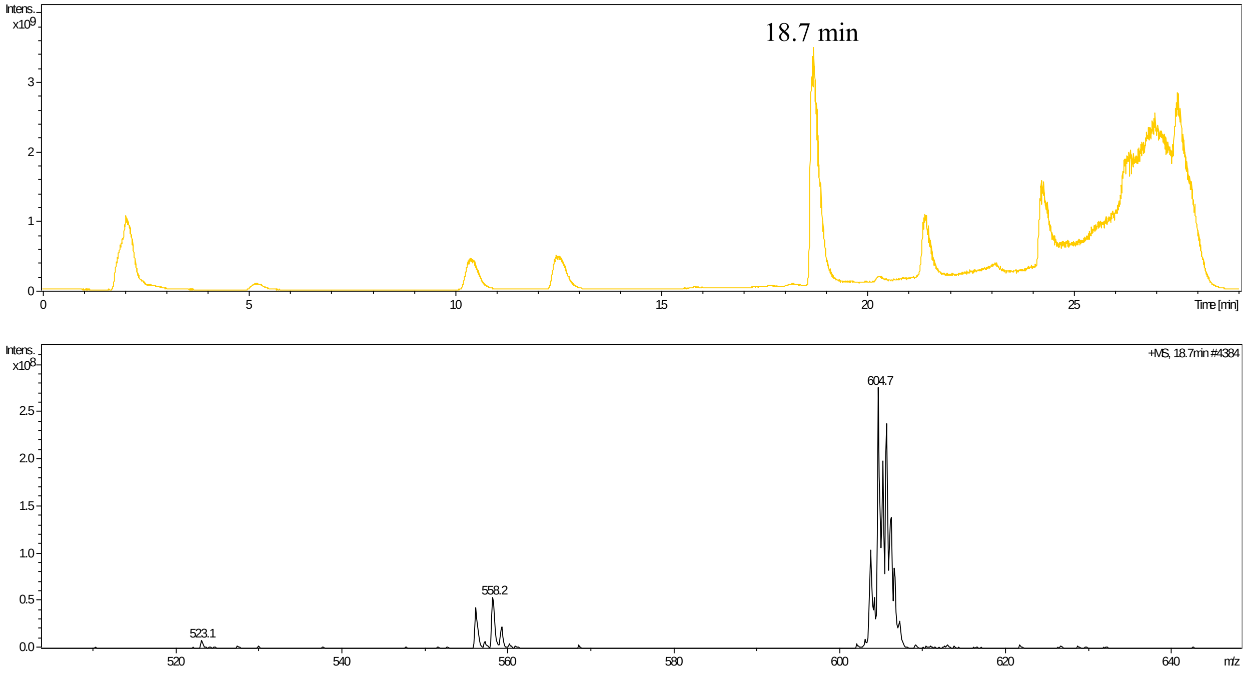

Figure S105. HPLC run of Ir-Bz complex **6c** (20 µM in 500 µM phosphate buffer, pH 7.2) after 0h (above); extracted MS compound spectrum at 18.7 min showing *m/z* according to $[((Cp^*)Ir)_2L(N\text{-Ac-Cys})]^{2+}$ (*m/z* 523.1, m_{ex} = 523.2) and $[((Cp^*)Ir)_2L(N\text{-Ac-Cys})_2]^{2+}$ (*m/z* 604.7, m_{ex} = 604.8) (below).

Chromatographic lipophilicity index φ_0

Table S2. Lipophilicity indices for all Ru, Rh, and Ir complexes.

	Ru (**3**)	Rh (**5**)	Ir (**6**)
Me (**a**)	5.51	5.91	5.75
An (**b**)	6.07	6.05	5.86
Bz (**c**)	6.04	6.28[1]	6.22
Np (**d**)	6.31	6.60	6.24

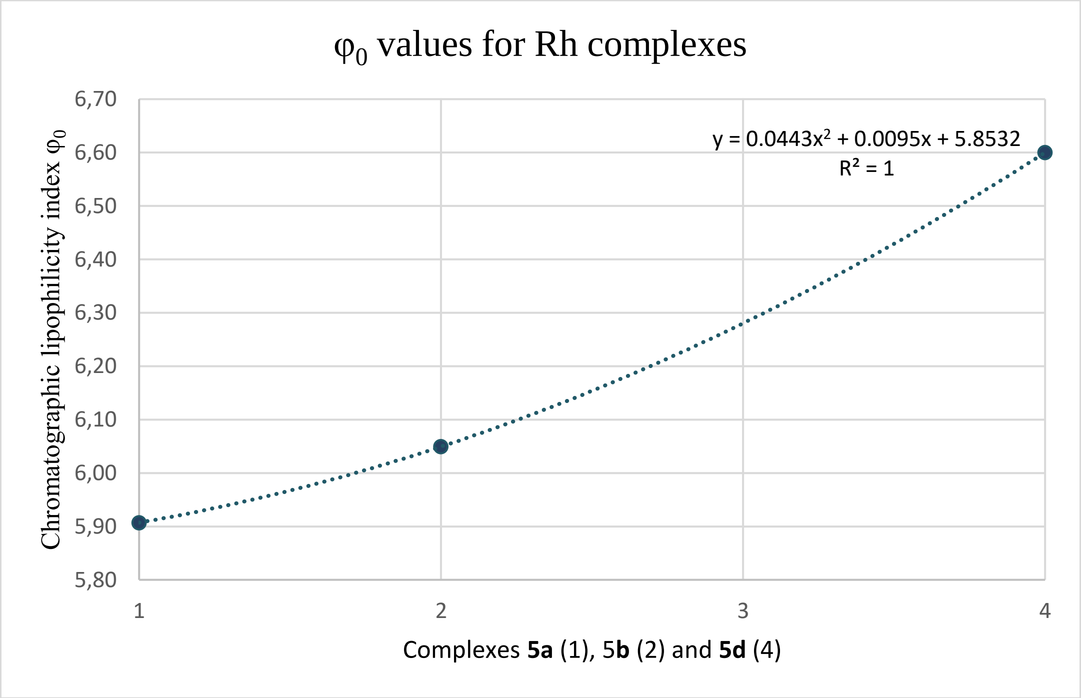

Chart S1. Extrapolation curve for the calculation of φ_0 for **5c**.

[1] φ_0 for Rh Bz complex **5c** was extrapolated from the experimentally determined values for all other Rh compounds. This was necessary due to technical problems with the HPLC system. The extrapolation curve can be seen in **Chart S1**.

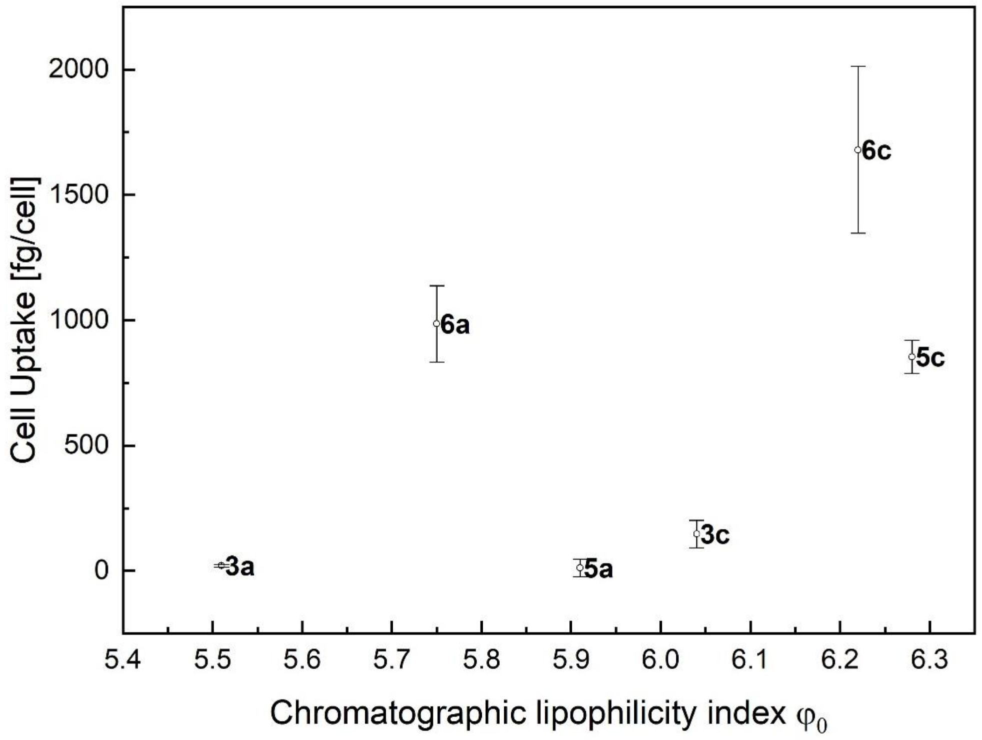

Figure S106. Scatter plot of chromatographic lipophilicity index *vs* cellular uptake in SW480 cells.

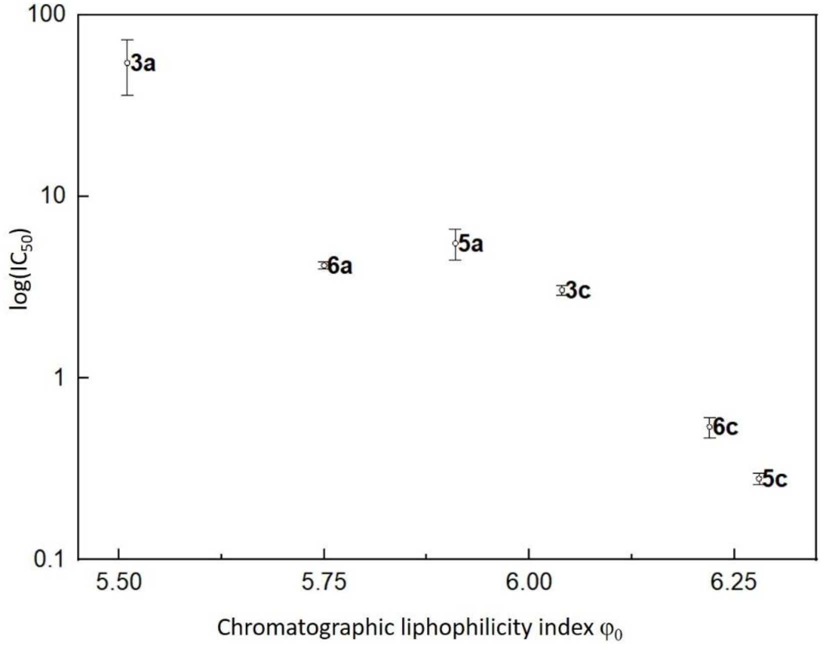

Figure S107. Scatter plot of chromatographic lipophilicity index *vs* log(IC_{50}) values in SW480 cells.

Cytotoxicity in cancer cell lines (monolayer cultures)

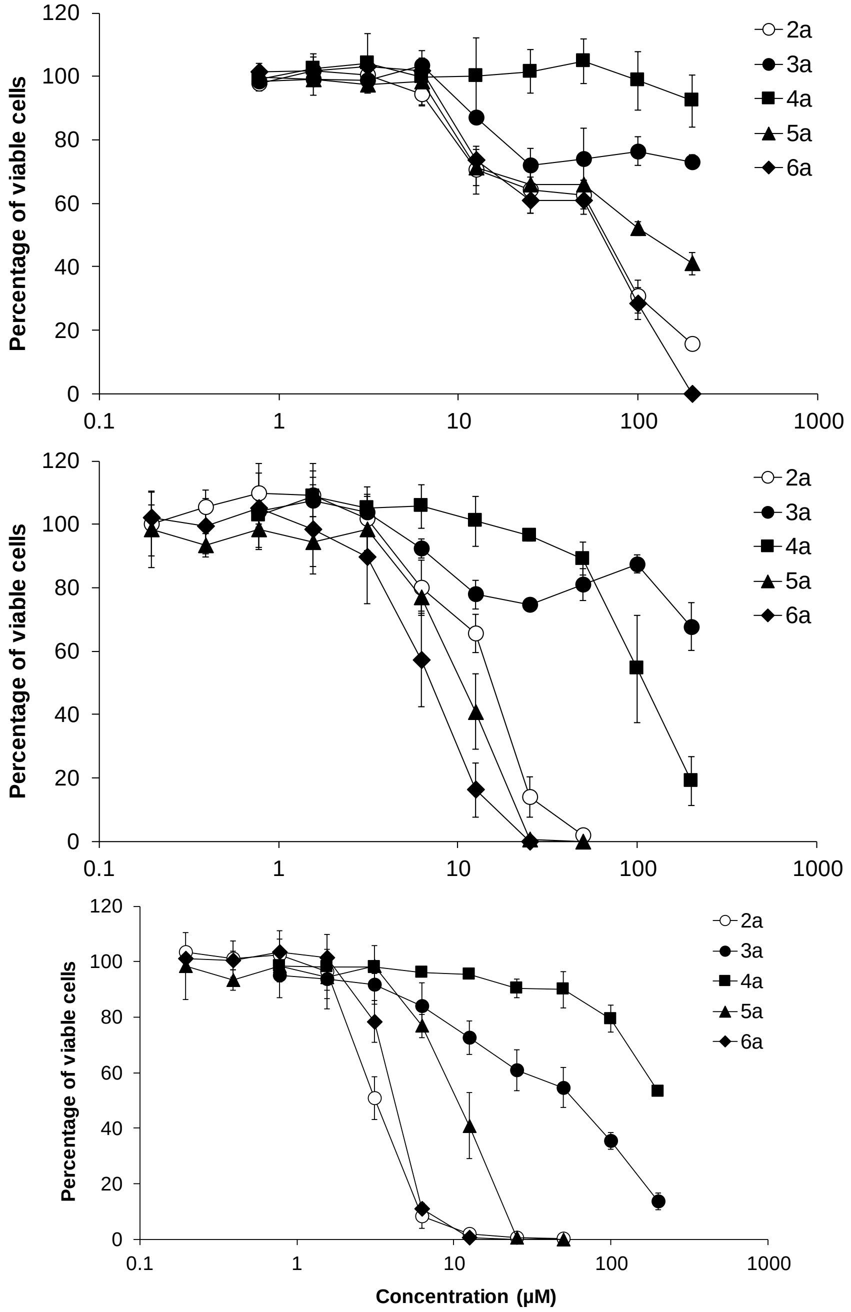

Figure S108. Concentration–effect curves of compounds **2a–6a** in monolayer cultures of A549 (top), CH1/PA-1 (middle) and SW480 (bottom) cells, obtained by the MTT assay (96 h exposure).

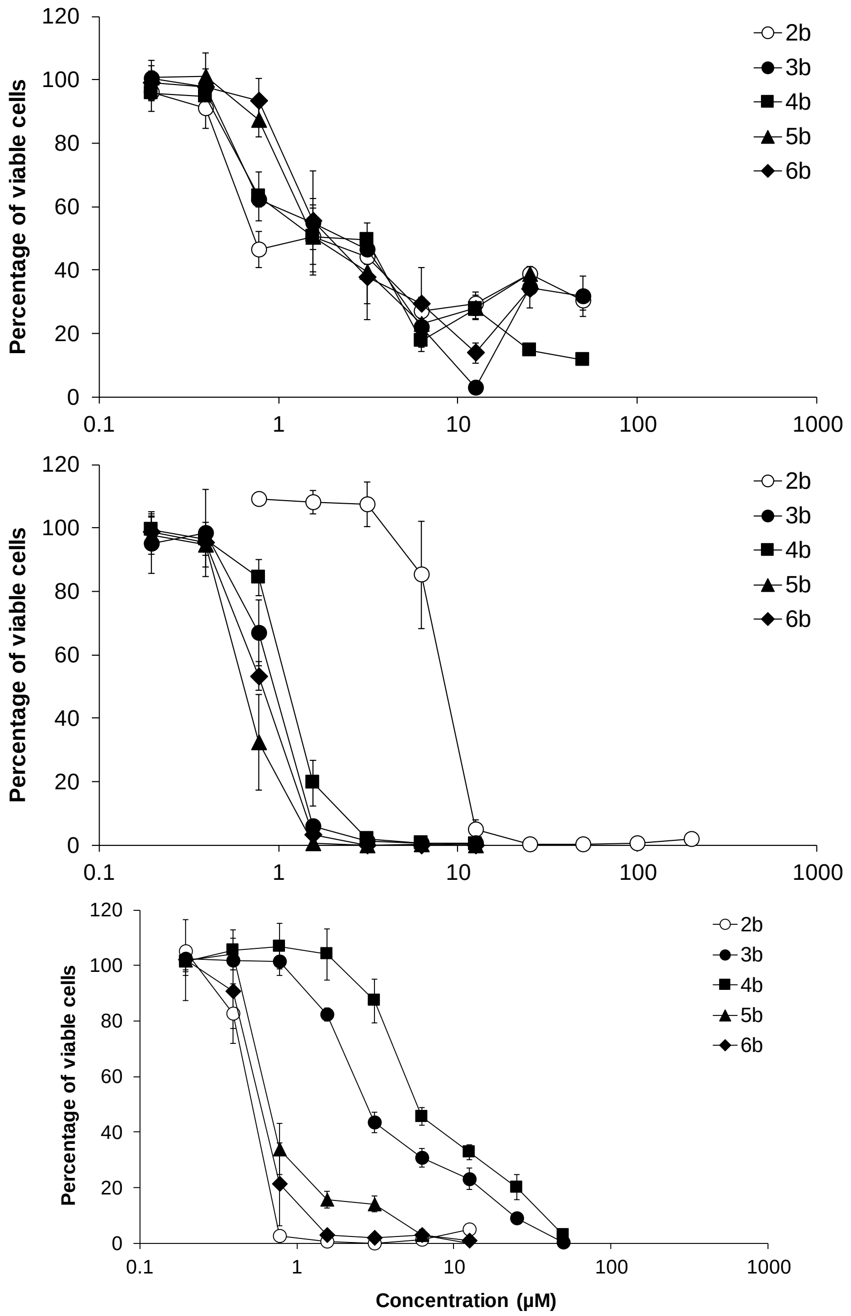

Figure S109. Concentration–effect curves of compounds **2b**–**6b** in monolayer cultures of A549 (top), CH1/PA-1 (middle) and SW480 (bottom) cells, obtained by the MTT assay (96 h exposure).

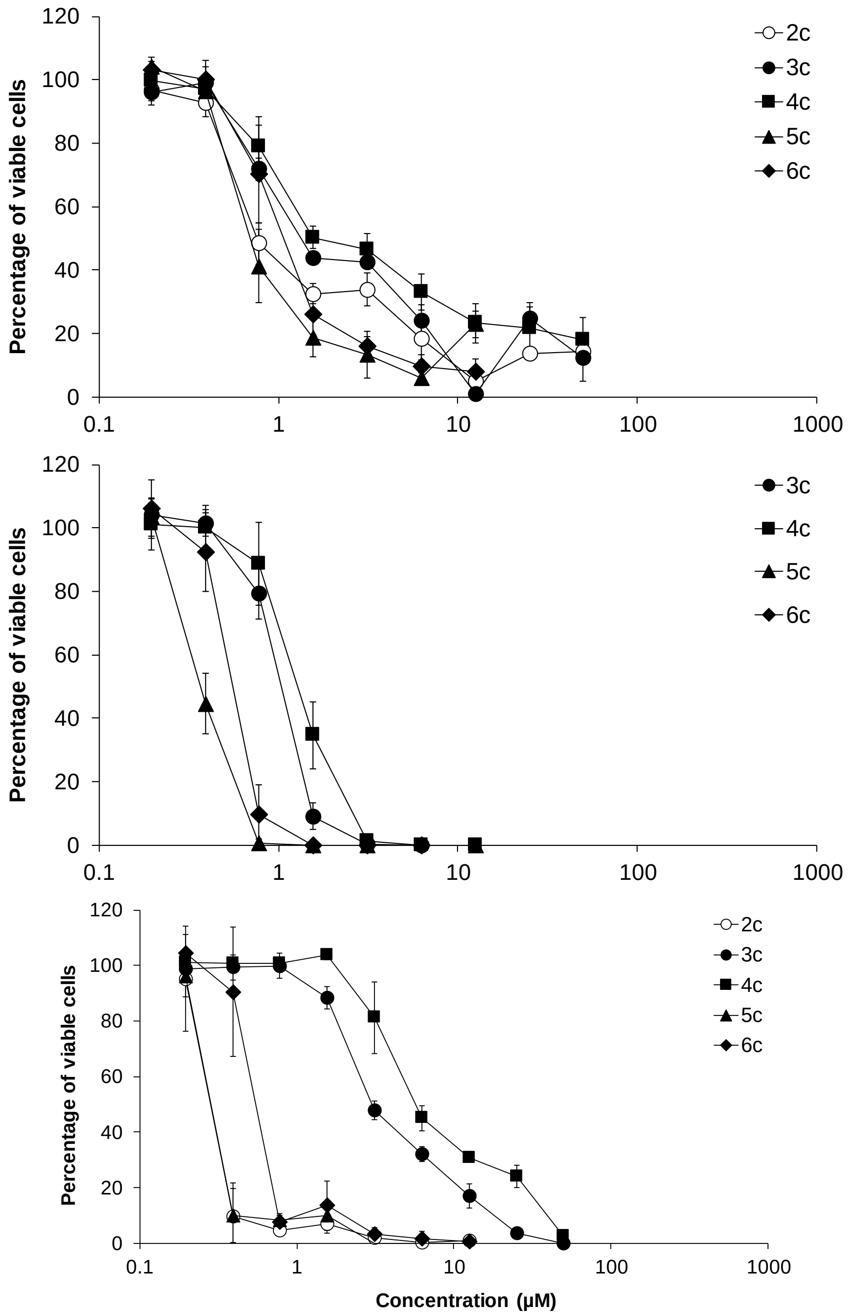

Figure S110. Concentration–effect curves of compounds **2c**–**6c** in monolayer cultures of A549 (top), CH1/PA-1 (middle) and SW480 (bottom) cells, obtained by the MTT assay (96 h exposure).

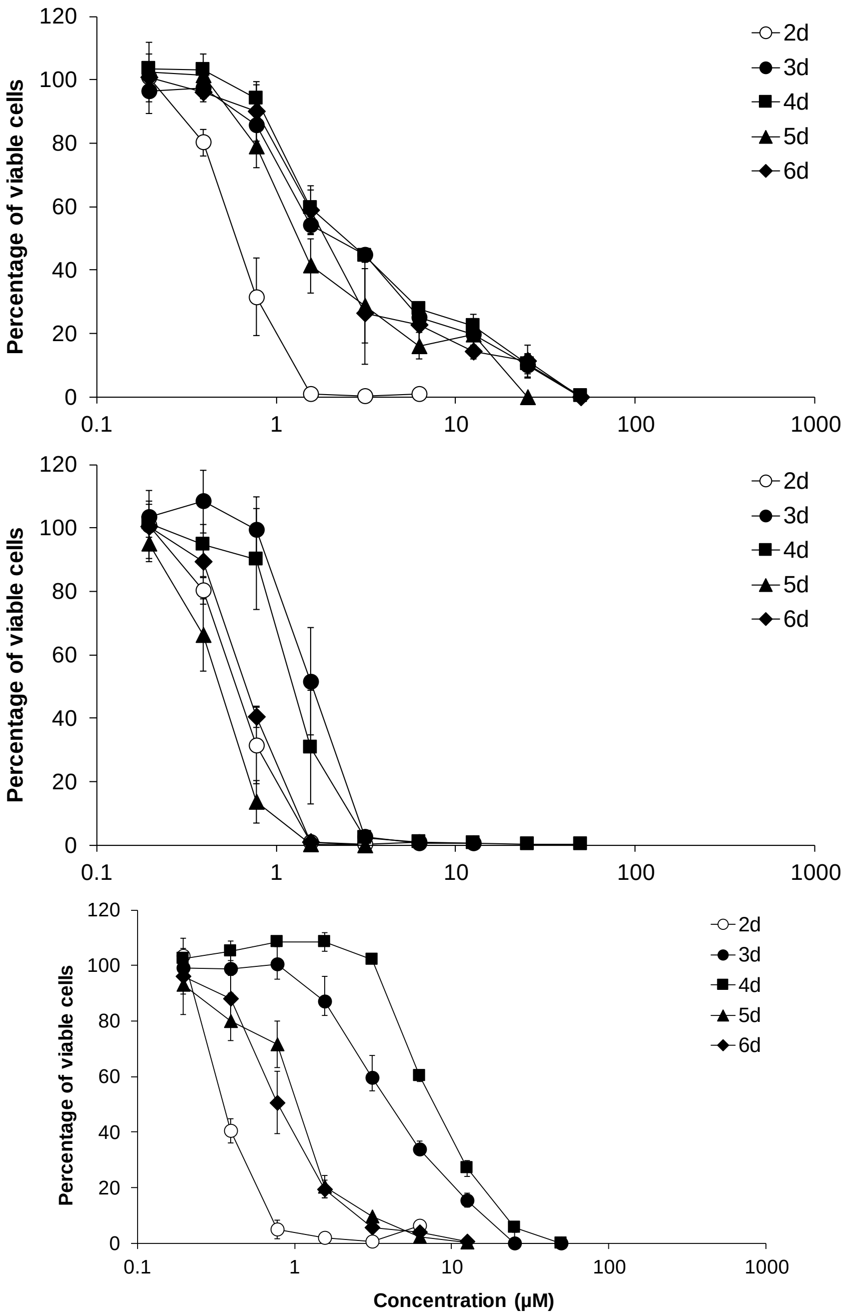

Figure S111. Concentration–effect curves of compounds **2d**–**6d** in monolayer cultures of A549 (top), CH1/PA-1 (middle) and SW480 (bottom) cells, obtained by the MTT assay (96 h exposure).

Cell cycle studies

Table S3. Impact of the tested compounds on the cell cycle distribution of CH1/PA-1 and SW480 cells upon 24 h incubation (mean ± SD). Etoposide was used as a positive control.

Cell cycle distribution (%)								
Compound	[C], µM	CH1/PA-1			[C], µM	SW480		
		G1, %	S, %	G2/M, %		G1, %	S, %	G2/M, %
Control (negative)	0	40 ± 2	36 ± 2	24 ± 4	0	48 ± 1	33 ± 2	19 ± 1
Benzyl subgroup: **2c** [ligand], **3c** [Ru], **5c** [Rh], **6c** [Ir]								
**2c** (DMSO[2])	2.5	43 ± 16	37 ± 5	19 ± 9	5	30 ± 1	39 ± 2	31 ± 3
	5	31 ± 5	37 ± 2	32 ± 4	10	27 ± 3	45 ± 2	27 ± 3
	10	31 ± 6	43 ± 8	26 ± 10	20	34 ± 5	49 ± 1	17 ± 3
**3c** (DMSO)	2.5	30 ± 1	47 ± 8	23 ± 6	2.5	45 ± 3	38 ± 6	17 ± 4
	5	27 ± 3	43 ± 3	30 ± 3	5	44 ± 2	36 ± 0	20 ± 3
	10	24 ± 6	47 ± 4	30 ± 7	10	48 ± 7	32 ± 7	21 ± 4
**5c** (DMSO)	1.25	40 ± 14	38 ± 7	22 ± 7	2.5	44 ± 10	36 ± 1	20 ± 6
	2.5	34 ± 5	42 ± 3	24 ± 4	5	44 ± 5	39 ± 1	17 ± 4
	5	30 ± 10	42 ± 6	28 ± 18	10	50 ± 2	34 ± 5	16 ± 5
**6c** (DMSO)	0.75	30 ± 4	38 ± 3	32 ± 4	0.75	40 ± 5	36 ± 2	24 ± 5
	1.25	30 ± 8	37 ± 6	33 ± 4	1.25	39 ± 13	38 ± 8	22 ± 3
	2.5	27 ± 4	39 ± 4	34 ± 3	2.5	43 ± 2	38 ± 5	19 ± 3

[2] In parentheses the solvent used to prepare the stock solutions of the drugs is stated. In case of DMSO application, the final concentrations on the cells never exceeded 0.5% of DMSO in MEM.

Methyl subgroup: **2a** [ligand], **5a** [Rh], **6a** [Ir],								
**2a** (DMSO)	5	33 ± 4	40 ± 3	27 ± 5	5	45 ± 1	32 ± 2	23 ± 0
	10	33 ± 4	41 ± 8	26 ± 6	10	41 ± 1	35 ± 2	24 ± 1
	20	35 ± 3	37 ± 9	28 ± 4	20	44 ± 3	33 ± 2	22 ± 2
**5a** (MEM)	10	28 ± 1	44 ± 5	28 ± 5	5	44 ± 1	30 ± 4	26 ± 2
	20	27 ± 5	41 ± 5	32 ± 4	10	38 ± 4	42 ± 1	20 ± 4
	40	26 ± 6	42 ± 1	31 ± 2	20	46 ± 3	42 ± 2	12 ± 1
**6a** (MEM)	10	29 ± 3	45 ± 8	26 ± 4	2.5	48 ± 2	32 ± 1	19 ± 3
	20	33 ± 4	41 ± 10	26 ± 9	5	41 ± 4	33 ± 9	26 ± 5
	40	28 ± 6	45 ± 5	26 ± 4	10	42 ± 4	42 ± 3	17 ± 3
Rhodium subgroup: **5b** (Rh-aniline), **5d** (Rh-naphthyl)								
**5b** (MEM)	2.5	22 ± 2	43 ± 9	35 ± 10	2.5	37 ± 17	40 ± 11	23 ± 7
	5	22 ± 6	37 ± 5	41 ± 4	5	26 ± 13	48 ± 7	27 ± 9
	10	19 ± 7	42 ± 1	39 ± 5	10	34 ± 10	49 ± 8	17 ± 6
**5d** (DMSO)	1.25	40 ± 10	44 ± 3	16 ± 9	2.5	38 ± 12	40 ± 3	22 ± 8
	2.5	38 ± 5	36 ± 7	27 ± 8	5	43 ± 10	37 ± 8	20 ± 3
	5	39 ± 6	40 ± 2	22 ± 5	10	48 ± 10	34 ± 9	18 ± 1
Positive control								
Etoposide (DMSO)	0.25	8 ± 5	15 ± 4	77 ± 10	0.25	9 ± 2	26 ± 4	65 ± 10
	0.5	4 ± 1	10 ± 3	85 ± 5	0.5	3 ± 1	19 ± 7	78 ± 6
	1	3 ± 3	10 ± 5	87 ± 6	1	6 ± 4	30 ± 15	65 ± 10

Table S4. Relative to negative control change in cell cycle distribution upon treatment with the tested compounds. The differences to values in the untreated control are calculated based on the means given in **Table S3**.

Relative change in cell cycle distribution (%)								
Compound	[C], µM	CH1/PA-1			[C], µM	SW480		
		G1, %	S, %	G2/M, %		G1, %	S, %	G2/M, %
Benzyl subgroup: **2c** (ligand), **3c** (Ru), **5c** (Rh), **6c** (Ir)								
**2c**	2.5	3	1	-5	5	-18	6	12
	5	-9	1	8	10	-21	12	8
	10	-9	7	2	20	-14	16	-2
**3c**	2.5	-10	11	-1	2.5	-3	5	-2
	5	-13	7	6	5	-4	3	1
	10	-16	11	6	10	0	-1	2
**5c**	1.25	0	2	-2	2.5	-4	3	1
	2.5	-6	6	0	5	-4	6	-2
	5	-10	6	4	10	2	1	-3
**6c**	0.75	-10	2	8	0.75	-8	3	5
	1.25	-10	1	9	1.25	-9	5	3
	2.5	-13	3	10	2.5	-5	5	0
Methyl subgroup: **2a** (ligand), **5a** (Rh), **6a** (Ir),								
**2a**	5	-7	4	3	5	-3	-1	4
	10	-7	5	2	10	-7	2	5
	20	-5	1	4	20	-4	0	3
**5a**	10	-12	8	4	5	-4	-3	7
	20	-13	5	8	10	-10	9	1

	40	-14	6	7	20	-2	9	-7
**6a**	10	-11	9	2	2.5	0	-1	0
	20	-7	5	2	5	-7	0	7
	40	-12	9	2	10	-6	9	-2
Rhodium subgroup: **5b** (Rh-aniline), **5d** (Rh-naphthyl)								
**5b**	2.5	-18	7	11	2.5	-11	7	4
	5	-18	1	17	5	-22	15	8
	10	-21	6	15	10	-14	16	-2
**5d**	1.25	0	8	-8	2.5	-10	7	3
	2.5	-2	0	3	5	-5	4	1
	5	-1	4	-2	10	0	1	-1
Positive control								
Etoposide	0.25	-32	-21	53	0.25	-39	-7	46
	0.5	-36	-26	61	0.5	-45	-14	59
	1	-37	-26	63	1	-42	-3	46
